# Anatomical Network Comparison of Human Upper and Lower, Newborn and Adult, and Normal and Abnormal Limbs, with Notes on Development, Pathology and Limb Serial Homology *vs*. Homoplasy

**DOI:** 10.1371/journal.pone.0140030

**Published:** 2015-10-09

**Authors:** Rui Diogo, Borja Esteve-Altava, Christopher Smith, Julia C. Boughner, Diego Rasskin-Gutman

**Affiliations:** 1 Department of Anatomy, Howard University College of Medicine, Washington, DC, United States of America; 2 Structure & Motion Laboratory, Department of Comparative Biomedical Sciences, Royal Veterinary College, London, United Kingdom; 3 Theoretical Biology Research Group, Cavanilles Institute of Biodiversity and Evolutionary Biology, University of Valencia, Valencia, Spain; 4 Department of Anatomy and Cell Biology, University of Saskatchewan, Saskatoon, Saskatchewan, Canada; The University of Tennessee Health Science Center, UNITED STATES

## Abstract

How do the various anatomical parts (modules) of the animal body evolve into very different integrated forms (integration) yet still function properly without decreasing the individual’s survival? This long-standing question remains unanswered for multiple reasons, including lack of consensus about conceptual definitions and approaches, as well as a reasonable bias toward the study of hard tissues over soft tissues. A major difficulty concerns the non-trivial technical hurdles of addressing this problem, specifically the lack of quantitative tools to quantify and compare variation across multiple disparate anatomical parts and tissue types. In this paper we apply for the first time a powerful new quantitative tool, Anatomical Network Analysis (AnNA), to examine and compare in detail the musculoskeletal modularity and integration of normal and abnormal human upper and lower limbs. In contrast to other morphological methods, the strength of AnNA is that it allows efficient and direct empirical comparisons among body parts with even vastly different architectures (e.g. upper and lower limbs) and diverse or complex tissue composition (e.g. bones, cartilages and muscles), by quantifying the spatial organization of these parts—their topological patterns relative to each other—using tools borrowed from network theory. Our results reveal similarities between the skeletal networks of the normal newborn/adult upper limb *vs*. lower limb, with exception to the shoulder *vs*. pelvis. However, when muscles are included, the overall musculoskeletal network organization of the upper limb is strikingly different from that of the lower limb, particularly that of the more proximal structures of each limb. Importantly, the obtained data provide further evidence to be added to the vast amount of paleontological, gross anatomical, developmental, molecular and embryological data recently obtained that contradicts the long-standing dogma that the upper and lower limbs are serial homologues. In addition, the AnNA of the limbs of a trisomy 18 human fetus strongly supports Pere Alberch's ill-named "logic of monsters" hypothesis, and contradicts the commonly accepted idea that birth defects often lead to lower integration (i.e. more parcellation) of anatomical structures.

## Introduction

A central question in evolutionary biology and biological anthropology is how various anatomical parts of the animal body evolved into very different forms such that all parts still fit together and function properly [1–5]. Ever since Bateson’s [6] and Olson & Miller’s [7] seminal works on these concepts, the idea of an animal’s body as a set of nested parts within parts (modularity) that maintain a level of autonomy to change while still growing and adapting in coordinated ways (integration) continues to gain support as a central mechanism of evolution [8–10]. These concepts are also tightly linked to questions about complexity and evolvability (the ability to respond to selective pressure). For instance, some authors argue that modularity enables flexibility because the direction and magnitude of evolutionary change among and within parts can vary without sacrificing function [9,11–17], while others argue that a higher integration (less parcellation) within an anatomical system, such as the head, may increase evolvability [2]. These issues are particularly crucial to understand the evolution of human limbs, which is notable among tetrapods and primates for the magnitude of morphological shifts in the musculoskeletal system, including the pervasive changes in the limbs associated with the acquisition of bipedalism [18–27].

However, paradoxically, our knowledge of morphological modularity, integration, complexity and evolvability remains limited even for the musculoskeletal system of our own species, because of the difficulty of studying the myriad interactions among the body's hard and soft-tissues [28,29]. Moreover, in no small part due to the challenge of managing and making sense of complex datasets, most studies have concentrated on a single body region. For instance, most primate modularity and integration studies focus on the head [3,29–42]; furthermore, the few studies on the modularity, integration and/or evolvability of the primate/human limbs have focused almost exclusively on the skeleton [20,21,24–27,43]. However, functional and morphological changes in human evolution involved the reorganization and evolution of both hard and soft tissues, and of traits that are not amenable to the type of measurements that are often done in such studies, such as the presence/absence of muscles and their attachments to bones. Consequently, a wide gap persists in our understanding of human musculoskeletal system as a whole. New approaches are thus needed to identify and compare patterns of organization, integration, modularity, evolvability and complexity between the muscles and bones of the limbs to have a more comprehensive and integrative view of the evolutionary history, as well as on the functional morphology, development and pathology, of the human body in the context of habitual bipedalism.

Anatomical network analysis (AnNA) of connectivity patterns (e.g. bone-bone, bone-muscle and muscle-muscle connections) is a powerful new tool to study these subjects. In contrast to evolutionary quantitative genetics and morphometric methods, a unique strength of AnNA is its direct comparisons among different tissues (e.g. bones, muscles) and body parts (e.g. head and upper and lower limbs). Specifically, AnNA evaluates connectivity patterns using tools and statistics borrowed from network theory, formalizing bones, muscles and their physical contacts as the nodes and links of a network model to assess the morphological organization of, and identify patterns of integration and modularity among, muscles and bones [44]. Importantly, AnNA is a formal framework to study morphological organization free of *a priori* assumptions about developmental, functional, and phylogenetic relationships among structures. We recently used AnNA to provide new insights on the musculoskeletal organization of the head of human adults, newborns, and fetuses with and without birth defects, as well as some preliminary comparisons between the head and upper limbs [29,45,46].

This present paper provides the first application of AnNA to examine and compare in detail the musculoskeletal modularity and integration of the upper and lower limbs (ULs, LLs) in the normal human adult and newborn phenotype and in a trisomy 18 (T18) human fetus. T18 (or Edward's syndrome) is a condition caused by the presence of an extra chromosome 18 and usually results in slow embryological growth and low birth weight. Phenotypic abnormalities often include overlapping fingers with clenched fists, problems with organ morphogenesis and a small head [47,48]. Many of T18 individuals die before birth and less than ten percent survive past their first year [47,48]. Importantly, T18 individuals usually have musculoskeletal anomalies, including the presence of supernumerary muscles and the absence or change of configuration/attachments of various muscles (for a recent review, see [49]). By using AnNA, we can therefore investigate in an original and more comprehensive quantitative way which anatomical integration/modularity patterns result from a known perturbed genetic condition that usually causes severe phenotypic malformations [49]. For instance, any changes in musculoskeletal integration and modularity resulting from phenotypic malformations found in a particular T18 individual can be studied by comparing the abnormal anatomical networks seen in that individual to those present in the normal phenotype. By including such comparisons, the present work will thus provide new information salient to these and other broader evolutionary and developmental issues that will further clarify the associations and tipping points between normal and abnormal development of the UL and LL, modularity, integration, and anatomical defects.

## Results

In this section we summarize and compare the AnNA of the three conditions studied: normal adult, normal newborn, and T18 fetus. We focus on the results of the quantification of basic network parameters, which are further detailed in *S1 Results* (for entire networks) and *S2 Results* (for proximal vs. distal divisions), with some remarks on the modular organization of each anatomical system. Specific modularity results are discussed extensively in the Discussion section in a broader developmental, functional, pathological and evolutionary context. One of the major goals of the present paper is to compare the musculoskeletal phenotype and network modules of the UL *vs*. the LL (see [50,51], and below). Therefore, results are presented such that we directly compare the ULs and LLs among all three conditions (normal adult, normal newborn, and T18). First, we refer to the normal adult/newborn UL (same phenotype) *vs*. the normal adult and newborn LL; then we compare the left UL vs. left LL of the T18 fetus; and lastly we compare the right UL vs. right LL of the T18 fetus. The descriptions of the network organization of the ULs will be briefer than those of the LLs as some aspects of the UL organization have been briefly described by us [49], contrary to that of the LL, which is provided for the first time here. For the ULs, we will mainly focus on aspects that were not investigated by us in the past [49] and/or that refer to information that is necessary for the subsequent comparisons with the LLs or the overall discussion of all obtained data. To compare how similar is the morphological organization of different networks we have calculated the % of similarity of their modular organization (*S3 Results*). The % of similarity is calculated according to the number of the same bones and muscles grouped in a same module in both networks (see Methods). Finally, we complement the comparative analysis by calculating the similarity between the modular organization identified using AnNA and different hypothesis of functional and developmental groups (*S4 Results*).

### The network organization of the normal newborn/adult ULs

**Fig 1:**
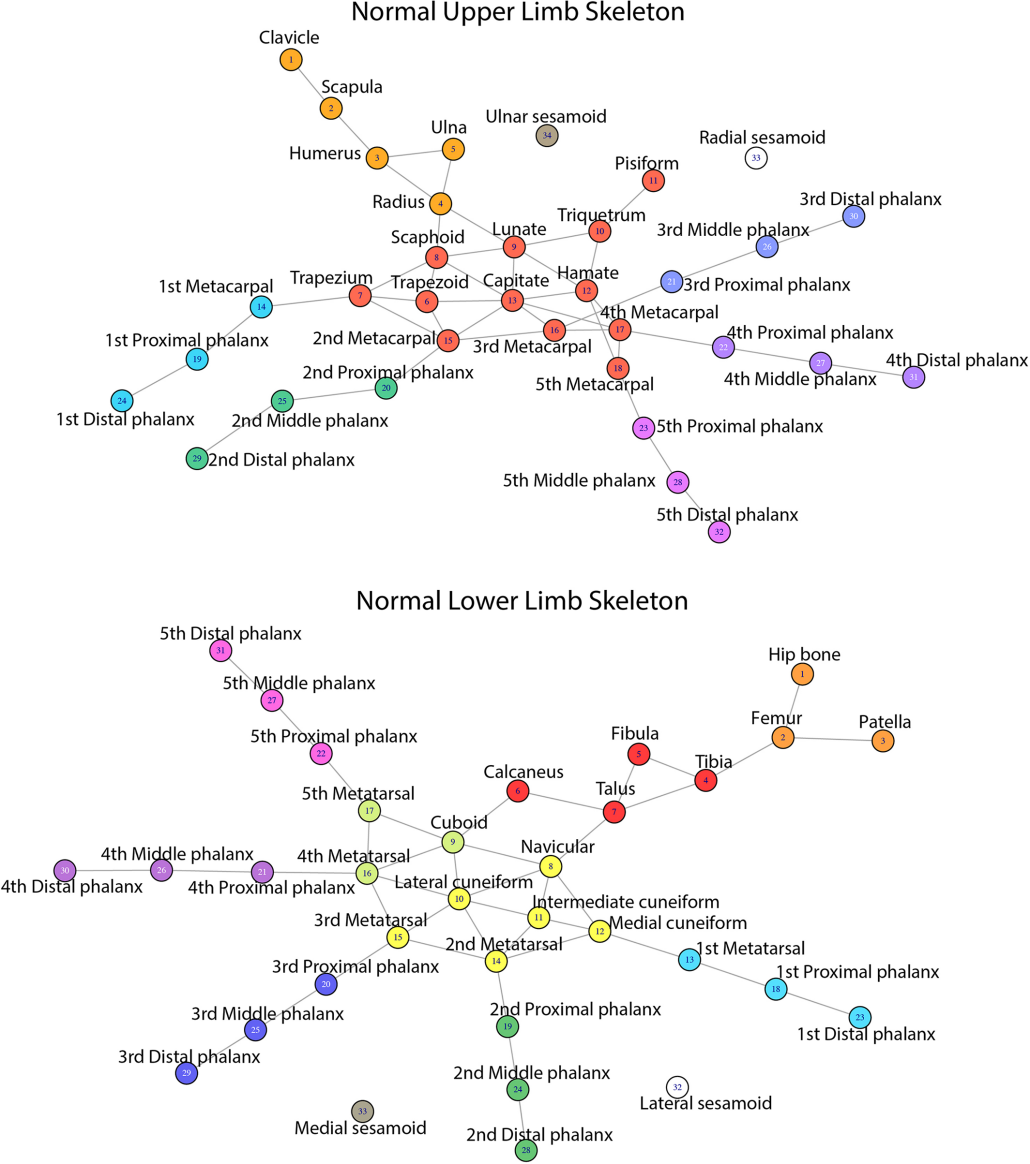
Network plots of normal adult left UL and left LL identified using AnNA. To see similar network plots for all the other skeletal, as well all the muscular and musculoskeletal, systems of all limbs, see S1–S24 Figs (see S1 Methods for labels).

**Fig 2:**
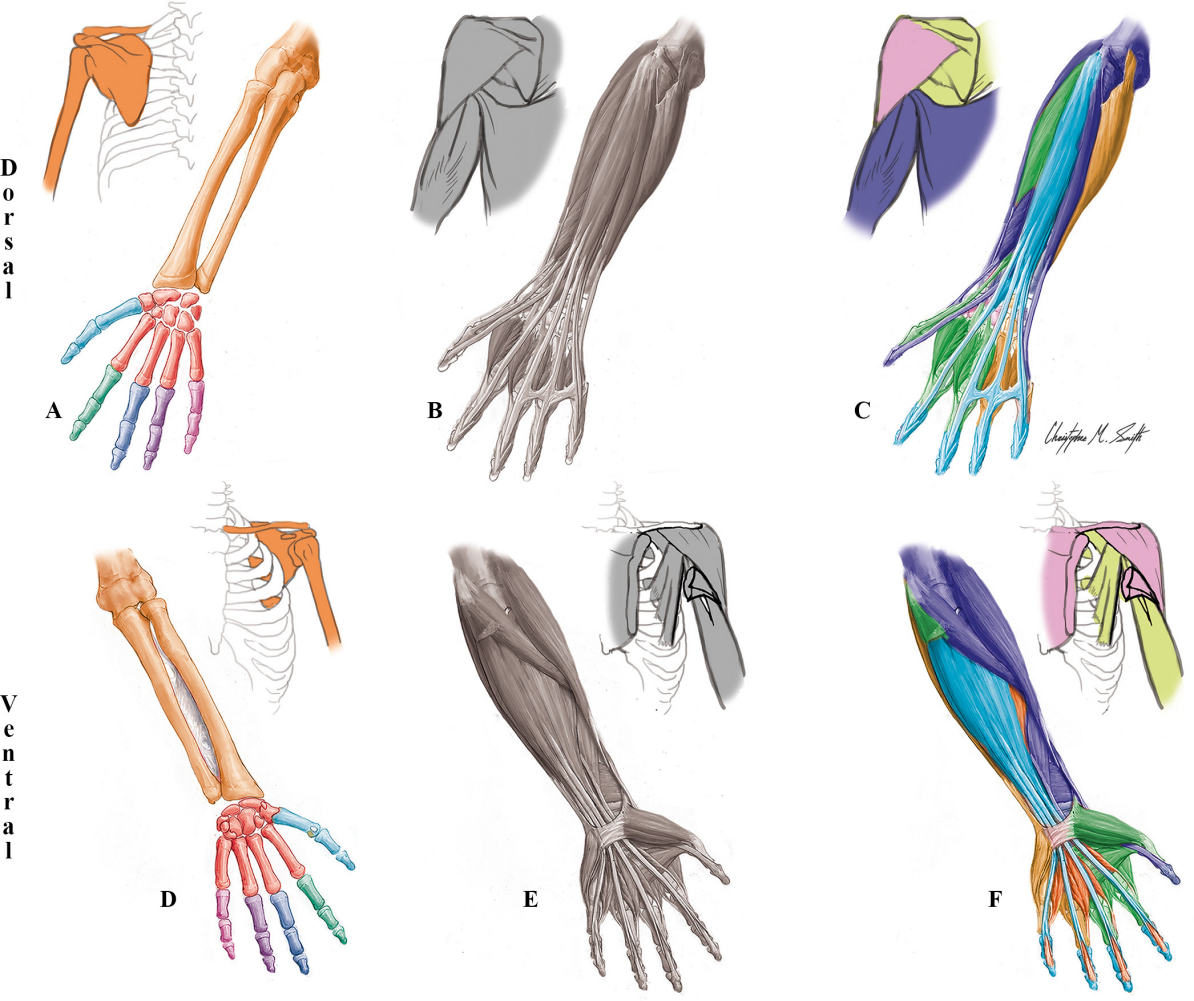
Modules of the normal newborn/adult left UL identified using AnNA. **A** to **C** dorsal (extensor) view; **D** to **F** ventral (flexor) view. It should be noted that the skeletal, muscular and musculoskeletal networks and modules of this left normal newborn UL are similar to the right one, and to both the left and right normal adult ULs). **A, D)** Skeletal network modules: in *red*, the wrist/metacarpals 2–5 module; in *turquoise*, the digit 1/metacarpal 1 module; in *dark green*, the digit 2 module; in *dark blue*, the digit 3 module; in *purple*, the digit 4 module; in *pink*, the digit 5 module; in *orange*, the girdle/arm/forearm module; in *white*, the radial sesamoid and ulnar sesamoid modules. **B, E)** Muscle network modules: no muscles create any modules in this network. **C, F)** Musculoskeletal network modules: in *turquoise*, the superficial flexor/extensor module; in *purple*, the arm-forearm-thumb movement module; in *dark green*, the digits 1-2-3 movement module; in *orange*, the profundus/lumbrical module; in *yellow*, the digits 4–5 movement module; in *light green*, the scapular module; and in *pink*, the clavicle movement module.

The skeletal, muscular and musculoskeletal networks and modules of the left and right ULs of the normal newborn are similar to each other, and similar to those of the adult, comprising 34 bones sparsely connected by 44 articulations, while the muscular system comprises 57 muscles connected by only four contacts and the musculoskeletal system comprises 91 bones and muscles connected by 184 contacts (Fig 1; Fig 2; Table 1). The skeletal system shows a tree-like, non-hierarchical organization characterized by a low density of connections and a few number of triangular loops (i.e. low clustering coefficient), which mainly occur among the less connected bones (i.e. negative C(*k*) exponent; see Methods). Muscles of the UL are barely connected with each other, but, when analyzed together with bones, the musculoskeletal system has a high number of triangular relations among bones and muscles, indicating that these parts are highly clustered, and hence they have a hierarchical, small-world organization.

**Table 1:** Phenotypic modules of the normal newborn/adult upper limb identified using AnNA.

***Musculoskeletal Network***			
**#**	**Bones/Cartilages**	**Muscles**	**Complex**
**1**	Hamate, Lunate, Pisiform, Triquetrum, 4th Metacarpal, 4th Proximal phalanx, 5th Metacarpal, 5th Proximal phalanx	Abductor digiti minimi, Flexor carpi ulnaris, Flexor digiti minimi brevis, Opponens digiti minimi, 2nd Palmar interossei, 3rd Palmar interossei, 4th Dorsal interossei	*Digits 4–5 movement*
**2**	Humerus, Radius, Ulna, 1st Distal phalanx	Abductor pollicis longus, Anconeus, Brachialis, Brachioradialis, Extensor carpi ulnaris, Extensor pollicis longus, Flexor pollicis longus, Latissimus dorsi, Palmaris longus, Pronator quadratus, Pronator teres, Supinator, Triceps brachii	*Arm-forearm-thumb movement*
**3**	Ulnar sesamoid, 2nd Metacarpal, 2nd Proximal phalanx, 3rd Metacarpal, 3rd Proximal phalanx, Capitate, Radial sesamoid, Scaphoid Trapezium, Trapezoid, 1st Metacarpal, 1st Proximal phalanx	Adductor pollicis, Extensor carpi radialis brevis, Extensor carpi radialis longus, Flexor carpi radialis, 1st Dorsal interossei, 1st Palmar interossei, 2nd Dorsal interossei, 3rd Dorsal interossei, Abductor pollicis brevis, Extensor pollicis brevis, Flexor pollicis brevis, Opponens pollicis, Adductor pollicis accessorius	*Digits 1-2-3 movement*
**4**	2nd Distal phalanx, 2nd Middle phalanx, 3rd Distal phalanx, 3rd Middle phalanx, 4th Distal phalanx, 4th Middle phalanx, 5th Distal phalanx, 5th Middle phalanx	Extensor digiti minimi, Extensor digitorum, Extensor indicis, Flexor digitorum superficialis	*Superficial flexor/extensor*
**5**	Scapula	Biceps brachii, Coracobrachialis, Infraspinatus, Levator scapulae, Pectoralis minor, Rhomboid major, Rhomboid minor, Serratus anterior, Subscapularis, Supraspinatus, Teres major, Teres minor	*Scapular*
**6**		Flexor digitorum profundus, 1st Lumbrical, 2nd Lumbrical, 3rd Lumbrical, 4th Lumbrical	*Profundus/lumbrical*
**7**	Clavicle	Pectoralis major, Deltoid, Subclavius	*Clavicle movement*
***Skeletal Network***			
**#**	**Bones/Cartilages**		**Complex**
**1**	5th Distal phalanx, 5th Middle phalanx, 5th Proximal phalanx		*Digit 5*
**2**	1st Distal phalanx, 1st Metacarpal, 1st Proximal phalanx		*Digit 1/metacarpal 1*
**3**	2nd Distal phalanx, 2nd Middle phalanx, 2nd Proximal phalanx		*Digit 2*
**4**	4th Distal phalanx, 4th Middle phalanx, 4th Proximal phalanx		*Digit 4*
**5**	3rd Distal phalanx, 3rd Middle phalanx, 3rd Proximal phalanx		*Digit 3*
**6**	Clavicle, Scapula, Humerus, Radius, Ulna		*Girdle/arm/forearm*
**7**	Radial sesamoid		*Radial sesamoid*
**8**	Ulnar sesamoid		*Ulnar sesamoid*
**9**	Trapezoid, Trapezium, Scaphoid, Capitate, 2nd Metacarpal, Triquetrum, Pisiform, Lunate, Hamate, 3rd Metacarpal, 4th Metacarpal, 5th Metacarpal		*Wrist/metacarpals 2–5*
***Muscular Network***			
**#**	**Muscles**		**Complex**
**1–57**	*Each muscle is a 1-muscle module (for a list of all muscles see SI*.*Labels)*		

### The network organization of the normal adult LLs

**Fig 3:**
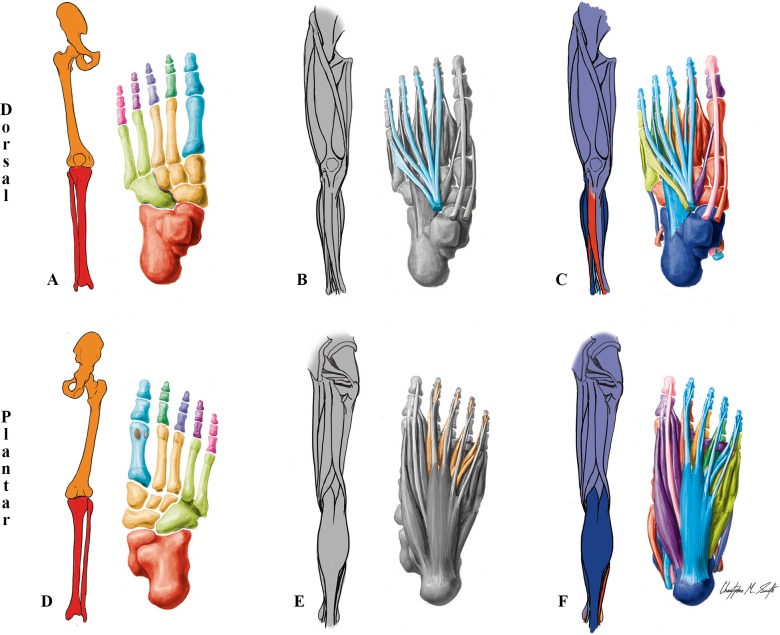
Modules of the normal adult left LL identified using AnNA. **A** to **C** dorsal (extensor) view; **D** to **F** ventral (flexor) view. It should be noted that the skeletal, muscular and musculoskeletal networks and modules of this left normal adult LL are similar to the right one. **A, D)** Skeletal network modules: in *light green*, the cuboid/ metarsals 4–5 module; in *pink*, *purple*, *dark blue* and *dark green*, the digit 5, 4, 3 and 2 modules; in *turquoise*, the digit 1/metatarsal 1 module; in *red*, the leg/proximal ankle module; in *light orange*, the tarsals/metatarsals 2–3 module; in *dark orange*, the girdle/thigh module; in *grey*, the lateral sesamoid and medial sesamoid modules. **B, E)** Muscle network modules: in *turquoise*, the extensor longus/fibularis module; in *orange*, the flexor longus/plantae/lumbrical module. **C, F)** Musculoskeletal network modules: in *turquoise*, the long flexor/extensor toes module; in *red*, the ankle/digit 2 movement module; in *magenta*, the hip-thigh-leg movement module; in *dark blue*, the ankle movement module; in *purple*, the big toe movement module; in *orange*, the digit 3 movement module; in *dark green*, the digit 4 movement module; in *light green*, the digit 5 movement module; in *pink*, the distal phalanx digit 1 movement module.

The networks and modules of the left and right LLs of the normal adult are similar to each other, but are different to those of the newborn. They include 33 bones connected by 41 articulations, 57 muscles connected by 6 contacts and a musculoskeletal system with 90 bones and muscles connected by 197 contacts. In terms of number of triangular loops (i.e. clustering coefficient) and density, the adult LL is in general somewhat similar to the adult/newborn UL (Fig 3; Table 2). However, the skeletal system of the adult LL is hierarchical, contrary to that of the adult/newborn UL (*S1 Results*), while the opposite pattern is interestingly seen in the musculoskeletal systems: both have a small-world organization, but that of the LL is non-hierarchical and that of the UL is hierarchical. As in the UL, muscles of the LL are barely connected to each other, but in contrast to the UL they do form two muscular modules including more than one muscle: a flexor *longus/plantae/lumbrical* module, and an *extensor longus/fibularis* module. Importantly, the quantitative comparative AnNA of the whole modular organization of the normal newborn/adult UL *vs*. the normal newborn/adult LL reveals a 93% similarity between their skeletal systems, but a profound difference (only 27% similarity) between their whole musculoskeletal systems (*S3 Results*). This indicates that the differences in morphological organization in the UL and LL are mainly related to the different muscular system and the way muscles attach to bones, thus creating a different overall musculoskeletal structural organization despite similarities between the skeleton of the UL and LL, specifically between the zeugopod (forearm/leg) and autopod (hand/foot) regions.

**Table 2:** Phenotypic modules in the lower limb of the normal adult.

***Musculoskeletal Network***			
**#**	**Bones/Cartilages**	**Muscles**	**Complex**
**1**	Cuboid, Intermediate cuneiform, Lateral cuneiform, Medial cuneiform, Navicular, 1st Metatarsal, 2nd Metatarsal, 2nd Proximal phalanx	Fibularis longus, Tibialis anterior, Tiblialis posterior, 1st Dorsal interossei, 1st Lumbrical, 2nd Dorsal interossei	*Ankle/digit 2 movement*
**2**	Femur, Patella, Hip bone, Tibia	Adductor brevis, Adductor longus, Adductor magnus, Biceps femoris, Gemellus inferior, Gemellus superior, Gluteus maximus, Gluteus medius, Gluteus minimus, Gracilis, Iliopsoas, Obturator externus, Obturator internus, Pectineus, Piriformis, Popliteus, Quadratus femoris, Rectus femoris, Sartorius, Semimembranosus, Semitendinosus, Tensor fasciae latae, Vastus intermedius, Vastus lateralis, Vastus medialis	*Hip-thigh-leg movement*
**3**	2nd Distal phalanx, 2nd Middle phalanx, 3rd Distal phalanx, 3rd Middle phalanx, 4th Distal phalanx, 4th Middle phalanx, 5th Distal phalanx, 5th Middle phalanx	Extensor digitorum brevis, Extensor digitorum longus, Flexor digitorum longus, Flexor digitorum brevis, Quadratus plantae, 4th Lumbrical	*Long flexor/extensor toes*
**4**	Calcaneus, Fibula, Talus	Fibularis brevis, Gastrocnemius, Plantaris, Soleus	*Ankle movement*
**5**	Lateral sesamoid, Medial sesamoid, 1st Proximal phalanx	Abductor hallucis, Adductor hallucis, Extensor hallucis brevis, Flexor hallucis brevis	*Big toe movement*
**6**	3rd Metatarsal, 3rd Proximal phalanx	1st Plantar interossei, 2nd Lumbrical, 3rd Dorsal interossei	*Digit 3 movement*
**7**	4th Metatarsal, 4th Proximal phalanx	2nd Plantar interossei, 3rd Lumbrical, 4th Dorsal interossei	*Digit 4 movement*
**8**	5th Metatarsal, 5th Proximal phalanx	Abductor digiti minimi, Flexor digiti minimi brevis, 3rd Plantar interossei, Fibularis tertius	*Digit 5 movement*
**9**	1st Distal phalanx	Extensor hallucis longus, Flexor hallucis longus	*Distal phalanx digit 1 movement*
***Skeletal Network***			
**#**	**Bones/Cartilages**		**Complex**
**1**	5th Proximal phalanx, 5th Middle phalanx, 5th Distal phalanx		*Digit 5*
**2**	1st Proximal phalanx, 1st Metatarsal, 1st Distal phalanx		*Digit 1/metatarsal 1*
**3**	2nd Proximal phalanx, 2nd Middle phalanx, 2nd Distal phalanx		*Digit 2*
**4**	4th Distal phalanx, 4th Middle phalanx, 4th Proximal phalanx		*Digit 4*
**5**	3rd Distal phalanx, 3rd Middle phalanx, 3rd Proximal phalanx		*Digit 3*
**6**	Tibia, Fibula, Calcaneus, Talus		*Leg/proximal ankle*
**7**	Navicular, Lateral cuneiform, Intermediate cuneiform, Medial cuneiform, 2nd Metatarsal, 3rd Metatarsal		*Tarsals/metatarsals 2–3*
**8**	Cuboid, 4th Metatarsal, 5th Metatarsal		*Cuboid/ metatarsals 4–5*
**9**	Hip bone, Femur, Patella		*Girdle/thigh*
**10**	Lateral sesamoid		Lateral sesamoid
**11**	Medial sesamoid		Medial sesamoid
***Muscular Network***			
**#**	**Muscles**		**Complex**
**1**	Lumbricals 1, 2, 3 and 4, Flexor digitorium longus, Quadratus plantae		*Flexor longus/plantae/lumbrical*
**2**	Extensor digitorum longus, Fibularis tertius		*Extensor longus/fibularis*
**3–51**	*Each other muscle is a 1-muscle module (for a list of all muscles see SI*.*Labels)*		

### The network organization of the normal newborn LLs

**Fig 4:**
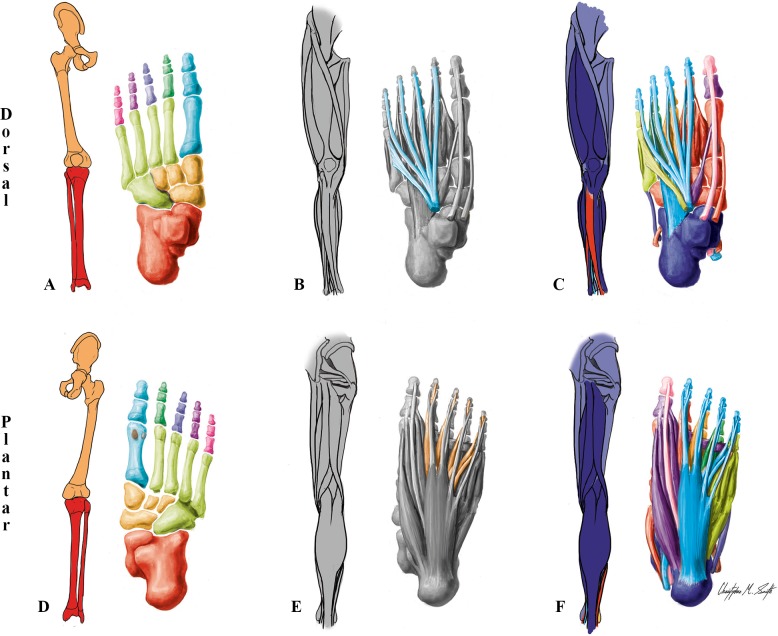
Modules of the normal newborn LL identified using AnNA. **A** to **C** dorsal (extensor) view; **D** to **F** ventral (flexor) view. It should be noted that the skeletal, muscular and musculoskeletal networks and modules of this left normal newborn LL are similar to the right one. **A, D)** Skeletal network modules: in *light green*, the cuboid/metarsals 2-3-4-5 module; in *pink*, *purple*, *dark blue* and *dark green*, the digit 5, 4, 3 and 2 modules; in *turquoise*, the digit 1/metatarsal 1 module; in *red*, the leg/proximal ankle module; in *light orange*, the tarsals module; in *dark orange*, the girdle/thigh module; in *grey*, the lateral sesamoid and medial sesamoid modules. **B, E)** Muscle network modules: as in normal adult (see Fig 2). **C, F)** Musculoskeletal network modules: in *turquoise*, the long flexor/extensor toes module; in *red*, the ankle/digit 2 movement module; in *light magenta*, the hip-thigh-leg movement module; in *dark magenta*, the leg/ankle movement module; in *purple*, the big toe movement module; in *orange*, the digit 3 movement module; in *dark green*, the digit 4 movement module; in *light green*, the digit 5 movement module; in *pink*, the distal phalanx digit 1 movement module.

The network organization and modules of the left and right LLs of the normal newborn are similar to each other, but are different to those of the adult. They include 35 bones connected by 46 articulations (in contrast to the adult LL, the pelvis is still clearly divided into three separate bones: the ischium, pubis and ilium), 57 muscles connected by 6 contacts (as in the adult LL), and a musculoskeletal system with 92 bones and muscles connected by 206 contacts (Fig 4; Table 3); different to adult LL due to the pelvic differences mentioned just above. Interestingly, the only difference between the adult and newborn LL (i.e. due to postnatal fusion of the three pelvic bones) leads to a significant difference in the network organization in the adult: the LL skeletal system acquires a hierarchical organization, being 88% similar to that of the newborn (*S3 Results*). This difference regarding the hierarchical organization is blurred when muscles are added: the musculoskeletal organization of the adult LL remains non-hierarchical during postnatal development, although the overall similarity between the musculoskeletal network of the newborn *vs*. adult LLs is actually slightly lower than that seen in the skeleton system (86%).

**Table 3:** Phenotypic modules in the lower limb of the normal newborn.

***Musculoskeletal Network***			
**#**	**Bones/Cartilages**	**Muscles**	**Complex**
**1**	Cuboid, Intermediate cuneiform, Lateral cuneiform, Medial cuneiform, Navicular, 1st Metatarsal, 2nd Metatarsal, 2nd Proximal phalanx	Fibularis longus, Tibialis anterior, Tiblialis posterior, 1st Dorsal interossei, 1st Lumbrical, 2nd Dorsal interossei	*Ankle/digit 2 movement*
**2**	Femur, Ischium, Pubis, Ilium	Adductor brevis, Adductor longus, Adductor magnus, Gemellus inferior, Gemellus superior, Gluteus maximus, Gluteus medius, Gluteus minimus, Gracilis, Iliopsoas, Obturator externus, Obturator internus, Pectineus, Piriformis, Quadratus femoris, Sartorius, Tensor fasciae latae	*Hip-thigh movement*
**3**	2nd Distal phalanx, 2nd Middle phalanx, 3rd Distal phalanx, 3rd Middle phalanx, 4th Distal phalanx, 4th Middle phalanx, 5th Distal phalanx, 5th Middle phalanx	Extensor digitorum brevis, Extensor digitorum longus, Flexor digitorum longus, Flexor digitorum brevis, Quadratus plantae, 4th Lumbrical	*Long flexor/extensor toes*
**4**	Calcaneus, Fibula, Talus, Tibia, Patella	Fibularis brevis, Gastrocnemius, Plantaris, Soleus, Biceps femoris, Popliteus, Rectus femoris, Semimembranosus, Semitendinosus, Vastus intermedius, Vastus lateralis, Vastus medialis	*Leg/ankle movement*
**5**	Lateral sesamoid, Medial sesamoid, 1st Proximal phalanx	Abductor hallucis, Adductor hallucis, Extensor hallucis brevis, Flexor hallucis brevis	*Big toe movement*
**6**	3rd Metatarsal, 3rd Proximal phalanx	1st Plantar interossei, 2nd Lumbrical, 3rd Dorsal interossei	*Digit 3 movement*
**7**	4th Metatarsal, 4th Proximal phalanx	2nd Plantar interossei, 3rd Lumbrical, 4th Dorsal interossei	*Digit 4 movement*
**8**	5th Metatarsal, 5th Proximal phalanx	Abductor digiti minimi, Flexor digiti minimi brevis, 3rd Plantar interossei, Fibularis tertius	*Digit 5 movement*
**9**	1st Distal phalanx	Extensor hallucis longus, Flexor hallucis longus	*Distal phalanx digit 1 movement*
***Skeletal Network***			
**#**	**Bones/Cartilages**		**Complex**
**1**	5th Proximal phalanx, 5th Middle phalanx, 5th DistEal phalanx		*Digit 5*
**2**	1st Proximal phalanx, 1st Metatarsal, 1st Distal phalanx		*Digit 1/metatarsal 1*
**3**	2nd Proximal phalanx, 2nd Middle phalanx, 2nd Distal phalanx		*Digit 2*
**4**	4th Distal phalanx, 4th Middle phalanx, 4th Proximal phalanx		*Digit 4*
**5**	3rd Distal phalanx, 3rd Middle phalanx, 3rd Proximal phalanx		*Digit 3*
**6**	Tibia, Fibula, Calcaneus, Talus		*Leg/proximal ankle*
**7**	Navicular, Lateral cuneiform, Intermediate cuneiform, Medial cuneiform		*Tarsals*
**8**	Cuboid, 4th Metatarsal, 5th Metatarsal, 2nd Metatarsal, 3rd Metatarsal		*Cuboid/ metatarsals 2-3-4-5*
**9**	Ilium, Ischium, Pubis, Femur, Patella		*Girdle/thigh*
**10**	Lateral sesamoid		Lateral sesamoid
**11**	Medial sesamoid		Medial sesamoid
***Muscular Network***			
**#**	**Muscles**		**Complex**
*Same than in the normal adult* (see SI.Tab 2)			

### The network organization of the T18 fetus left UL

**Fig 5:**
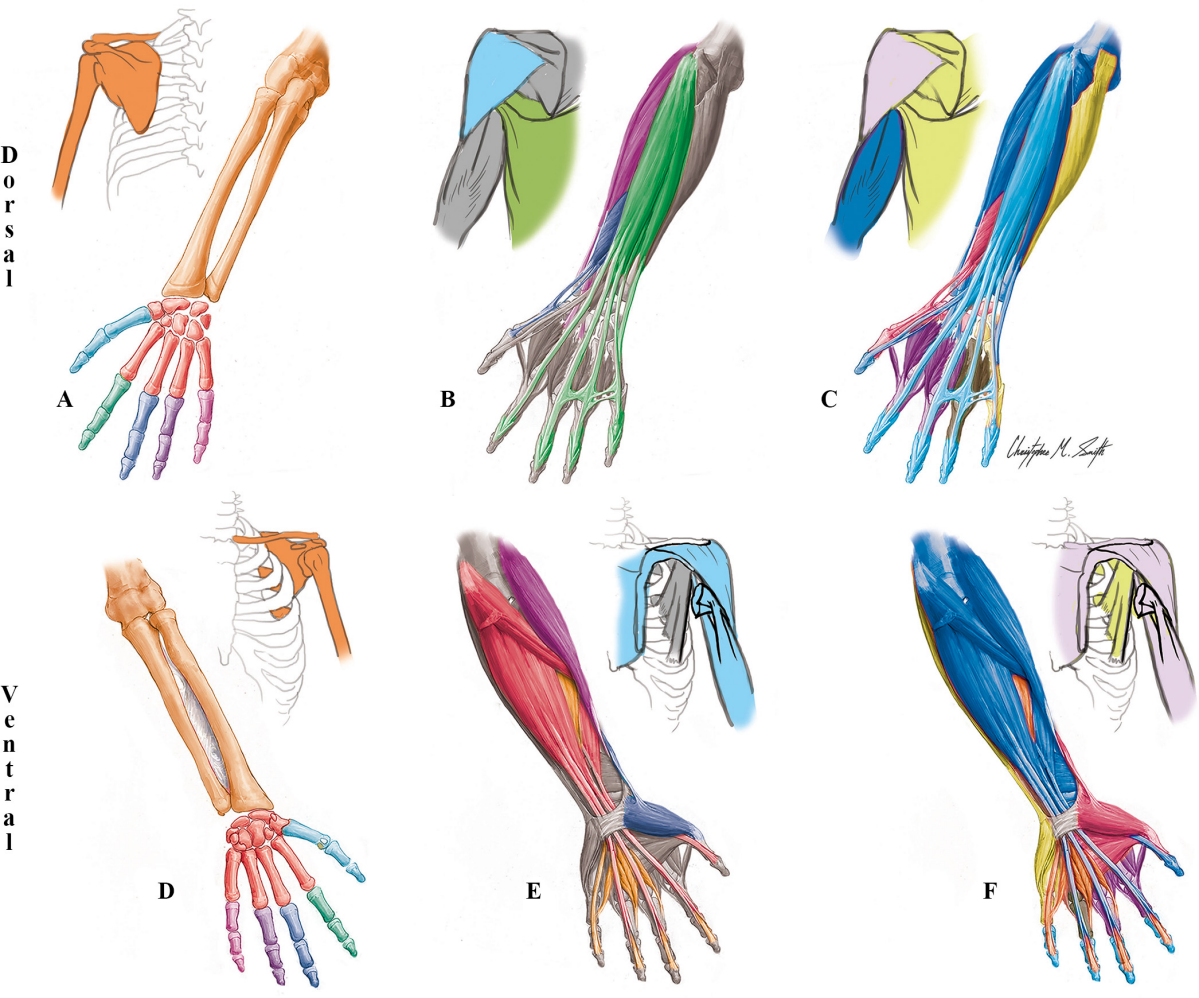
Modules of the T18 cyclopic fetus left UL identified using AnNA. **A** to **C** dorsal (extensor) view; **D** to **F** ventral (flexor) view. **A, D)** Skeletal network modules: as in normal adult/newborn (see Fig 1). **B, E)** Muscle network modules: in *dark green*, the ulnar extensor module; in *purple*, the radial extensor module; in *dark blue*, the thumb module; in *red*, the flexor module; in *orange*, the profundus/lumbrical module; in *light blue*, the biceps/deltopectoral module; and in *light green*, the latissimus dorsi/teres major module. **C, F)** Musculoskeletal network modules: in *blue*, the forearm-thumb movement module; in *purple*, the digits 2–3 movement module; in *turquoise*, the extensor module; in *red*, the thumb movement module; in *orange*, the profundus/lumbrical module; in *brown*, the digit 4 movement module; in *light green*, the scapular module; in *pink*, the clavicle movement/biceps module; and in *yellow*, the digit 5 movement module.

There are no differences in the morphological organization of the skeletal system of the left UL of the T18 fetus, of the right UL of this fetus, and of the left and right ULs of the normal newborn/adult. In contrast, the muscular system comprises 55 muscles and 20 contacts among them: two less muscles, and 16 more muscle contacts, than in the normal newborn/adult. This increase in contacts is sufficient to generate a small-world, hierarchical organization, suggesting that potential modules might be morphologically meaningful (e.g. in the context of the pathological fusion of muscles). The musculoskeletal system comprises 89 bones and muscles connected by 186 contacts (Fig 5; Table 4). As a consequence of more muscle-muscle connections in the T18 fetus left UL than in the normal newborn/adult UL, the musculoskeletal system also shows a higher degree of connections, as well as a small-world, hierarchical organization, but displays more parcellation (i.e. more modules: 9 vs. 7) than the normal phenotype. Despite these muscular and musculoskeletal differences, there is still a recognizable musculoskeletal network similarity between the T18 left UL and normal newborn UL (71% similarity). That is, the whole network organization of the T18 left UL is far from being chaotic.

**Table 4:** Phenotypic modules of the T18 fetus left upper limb identified using AnNA.

***Musculoskeletal Network***			
**#**	**Bones/Cartilages**	**Muscles**	**Complex**
**1**	Capitate, Lunate, Radial sesamoid, Scaphoid, Trapezium, Trapezoid, Ulnar sesamoid, 1st Metacarpal, 1st Proximal phalanx	Abductor pollicis brevis, Abductor pollicis longus, Adductor pollicis, Adductor pollicis accessorius, Extensor pollicis brevis, Flexor pollicis brevis, Opponens pollicis	*Thumb movement*
**2**	2nd Metacarpal, 2nd Proximal phalanx, 3rd Metacarpal, 3rd Proximal phalanx	Musculous interosseous accessorius, 1st Dorsal interossei, 1st Palmar interossei, 2nd Dorsal interossei, 3rd Dorsal interossei	*Digits 2–3 movement*
**3**	2nd Distal phalanx, 2nd Middle phalanx, 3rd Distal phalanx, 3rd Middle phalanx, 4th Distal phalanx, 4th Middle phalanx, 5th Distal phalanx, 5th Middle phalanx	Extensor digiti minimi, Extensor digitorum, Extensor indicis	*Extensor*
**4**	Humerus, Radius, Ulna, 1st Distal phalanx	Anconeus, Brachialis, Brachioradialis, Extensor carpi radialis brevis, Extensor carpi radialis longus, Extensor carpi ulnaris, Extensor pollicis longus, Flexor carpi radialis, Flexor digitorum superficialis, Flexor pollicis longus, Pronator quadratus, Pronator teres, Supinator, Triceps brachii	*Forearm-thumb movement*
**5**	Hamate, Pisiform, Triquetrum, 5th Metacarpal, 5th Proximal phalanx	Abductor digiti minimi, Flexor carpi ulnaris, Flexor digiti minimi brevis, Opponens digiti minimi, 3rd Palmar interossei	*Digit 5 movement*
**6**	Scapula	Coracobrachialis, Infraspinatus, Latissimus dorsi, Levator scapulae, Pectoralis minor, Rhomboid major minor, Serratus anterior, Subscapularis, Supraspinatus, Teres major, Teres minor	*Scapular*
**7**		Flexor digitorum profundus, 2nd Lumbrical, 3rd Lumbrical, 4th Lumbrical	*Profundus/lumbrical*
**8**	4th Metacarpal, 4th Proximal phalanx	2nd Palmar interossei, 4th Dorsal interossei	*Digit 4 movement*
**9**	Clavicle	Subclavius, Deltoid, Pectoralis major, Biceps brachii	*Clavicle movement/biceps*
***Skeletal Network***			
*Same than in the normal adult/newborn* (see SI.Tab 1)			
***Muscular Network***			
**#**	**Muscles**		**Complex**
**1**	Abductor pollicis brevis, Abductor pollicis longus, Extensor pollicis brevis, Flexor pollicis brevis, Opponens pollicis		*Thumb*
**2**	Flexor carpi radialis, Flexor digitorium superficialis, Flexor pollicis longus, Pronator teres		*Flexor*
**3**	2nd Lumbrical, 3rd Lumbrical, 4th Lumbrical, Flexor digitorium profundus		*Profundus/lumbrical*
**4**	Biceps brachii, Deltoid, Pectoralis major		*Biceps/deltopectoral*
**5**	Brachioradialis, Extensor carpi radialis brevis, Extensor carpi radialis longus		*Radial extensor*
**6**	Extensor carpi ulnaris, Extensor digiti minimi, Extensor digitorum		*Ulnar extensor*
**7**	Latissimus dorsi, Teres major		*Latissimus/teres major*
**8–38**	*Each other muscle is a 1-muscle module (for a list of all muscles see SI*.*Labels)*		

### The network organization of the T18 fetus left LL

**Fig 6:**
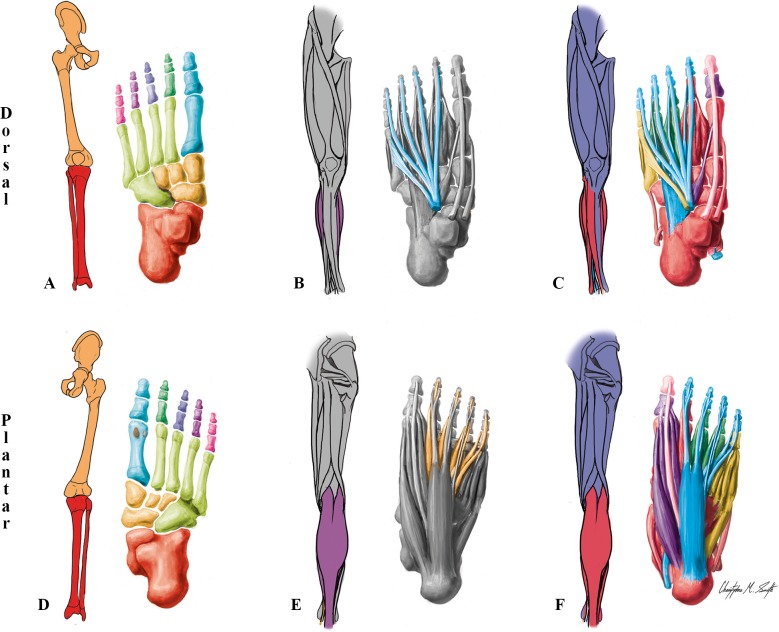
Modules of the T18 cyclopic fetus left LL identified using AnNA. **A** to **C** dorsal (extensor) view; **D** to **F** ventral (flexor) view. **A, D)** Skeletal network modules: as in normal newborn (see Fig 3). **B, E)** Muscle network modules: in *turquoise*, the extensor longus/fibularis module; in *orange*, the flexor longus/plantae/lumbrical module; in *purple*, the gastrocnemius/soleus module. **C, F)** Musculoskeletal network modules: in *turquoise*, the long flexor/extensor toes module; in *red*, the ankle/digit 2 movement module; in *magenta*, the hip-thigh-leg movement module; in *purple*, the big toe movement module; in *dark green*, the digits 3–4 movement module; in *yellow*, the digit 5 movement module; in *pink*, the distal phalanx digit 1 movement module.

There are no differences in the network organization of the skeletal system of the T18 left LL, the T18 right LL, and the normal newborn LL. The only difference between the muscular systems of the T18 left and the normal newborn LLs is that, in the T18 left LL (and also the T18 right LL), the gastrocnemius and soleus muscles are fused, forming a single module instead of two 1-muscle modules, in a total of 50 muscular modules (*vs*. 51 in the normal newborn). This differences, and other differences concerning the bone-muscle connections in the T18 left LL, result in a musculoskeletal network with two fewer modules (7 *vs*. 9) than that of the normal newborn LL, although this network keeps a small world, non-hierarchical organization as seen in the normal newborn LL (Fig 6; Table 5). These muscular and musculoskeletal differences account for only 21% difference (i.e. there is a 79% similarity) between the T18 left LL and the normal newborn LL (*S3 Results*).

**Table 5:** Phenotypic modules in the left lower limb of the T18 fetus.

***Musculoskeletal Network***			
**#**	**Bones/Cartilages**	**Muscles**	**Complex**
**1**	Cuboid, Intermediate cuneiform, Lateral cuneiform, Medial cuneiform, Navicular, 1st Metatarsal, 2nd Metatarsal, 2nd Proximal phalanx, Calcaneus, Fibula, Talus	Fibularis longus, Tibialis anterior, Tiblialis posterior, 1st Dorsal interossei, 1st Lumbrical, 2nd Dorsal interossei, Fibularis brevis, Gastrocnemius, Plantaris, Soleus	*Ankle/digit 2 movement*
**2**	Femur, Ischium, Pubis, Ilium, Tibia, Patella	Adductor brevis, Adductor longus, Adductor magnus, Gemellus inferior, Gemellus superior, Gluteus maximus, Gluteus medius, Gluteus minimus, Gracilis, Iliopsoas, Obturator externus, Obturator internus, Pectineus, Piriformis, Quadratus femoris, Sartorius, Tensor fasciae latae, Biceps femoris, Popliteus, Rectus femoris, Semimembranosus, Semitendinosus, Vastus intermedius, Vastus lateralis, Vastus medialis	*Hip-thigh-leg movement*
**3**	2nd Distal phalanx, 2nd Middle phalanx, 3rd Distal phalanx, 3rd Middle phalanx, 4th Distal phalanx, 4th Middle phalanx, 5th Distal phalanx, 5th Middle phalanx	Extensor digitorum brevis, Extensor digitorum longus, Flexor digitorum longus, Flexor digitorum brevis, Quadratus plantae, 4th Lumbrical	*Long flexor/extensor toes*
**4**	Lateral sesamoid, Medial sesamoid, 1st Proximal phalanx	Abductor hallucis, Adductor hallucis, Extensor hallucis brevis, Flexor hallucis brevis	*Big toe movement*
**5**	3rd Metatarsal, 3rd Proximal phalanx, 4th Metatarsal, 4th Proximal phalanx	1st Plantar interossei, 2nd Lumbrical, 3rd Dorsal interossei, 2nd Plantar interossei, 3rd Lumbrical, 4th Dorsal interossei	*Digits 3–4 movement*
**6**	5th Metatarsal, 5th Proximal phalanx	Abductor digiti minimi, Flexor digiti minimi brevis, 3rd Plantar interossei, Fibularis tertius	*Digit 5 movement*
**7**	1st Distal phalanx	Extensor hallucis longus, Flexor hallucis longus	*Distal phalanx digit 1 movement*
***Skeletal Network***			
**#**	**Bones/Cartilages**		**Complex**
**1–11** *Same than in the normal newborn* (see SI.Tab 3)			
***Muscular Network***			
**#**	**Muscles**		**Complex**
**1**	1st Lumbrical, 2nd Lumbrical, 3rd Lumbrical, 4th Lumbrical, Flexor digitorum longus, Quadratus plantae		*Flexor longus/plantae/lumbrical*
**2**	Extensor digitorum longus, Fibularis tertius		*Extensor longus/fibularis*
**3**	Gastrocnemius, Soleus		*Gastrocnemius/soleus*
**3–50**	*Each other muscle is a 1-muscle module (for a list of all muscles see SI*.*Labels)*		

### The network organization of the T18 fetus right UL

**Fig 7:**
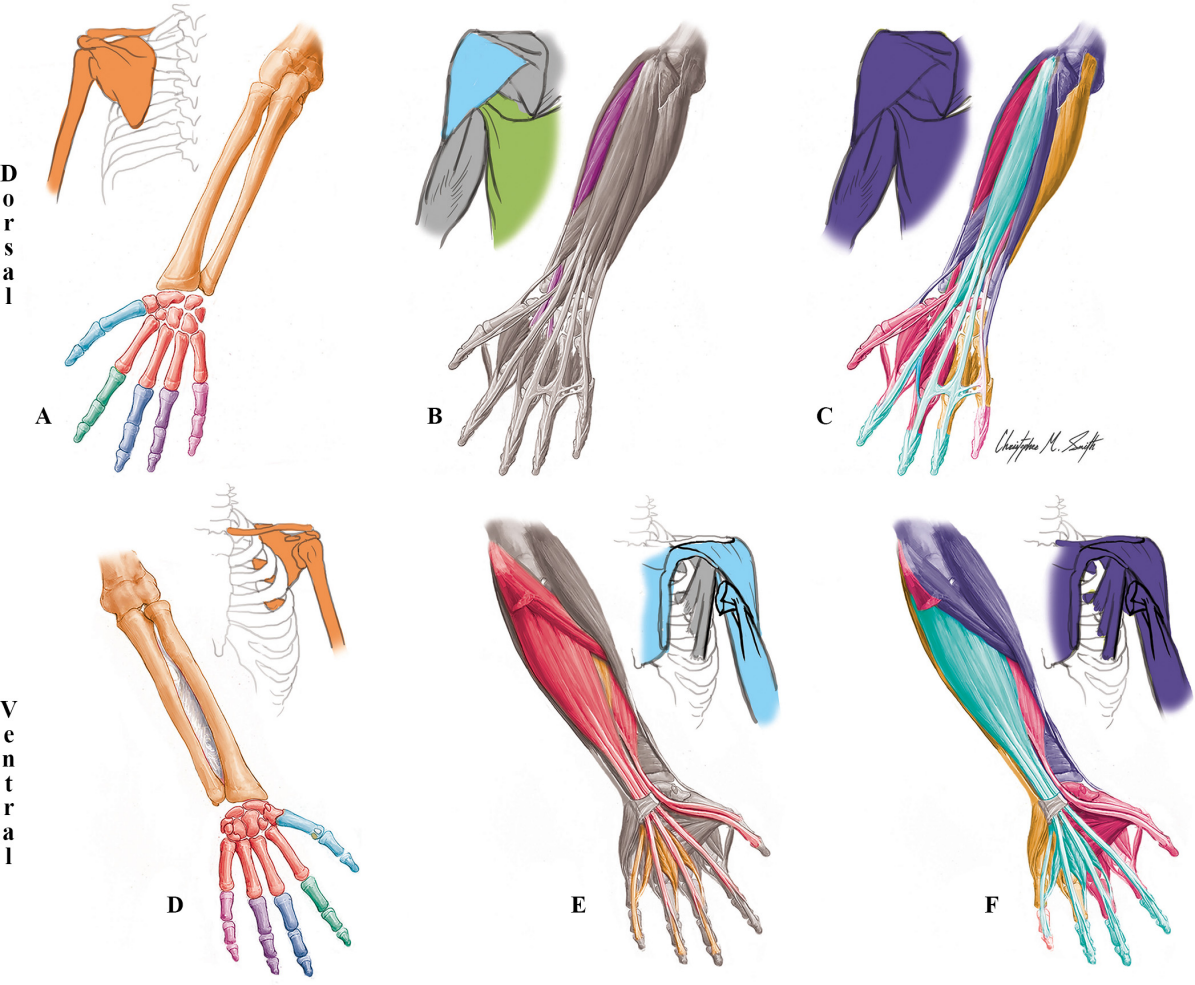
Modules of the T18 cyclopic fetus right UL identified using AnNA. **A** to **C** dorsal (extensor) view; **D** to **F** ventral (flexor) view. A horizontal flip was done with Photoshop, so the modules of this right UL can be more easily compared with those of the left ULs shown in Figs 1 and 4. **A, D)** Skeletal network modules: as in normal newborn/adult (see Fig 1). **B, E)** Muscle network modules: in *purple*, the extensor carpi radialis module; in *red*, the flexor module; in *orange*, the profundus/lumbrical module; in *light green*, the latissimus/teres major module; and in *light blue*, the biceps/coracodeltopectoral module. **C, F)** Musculoskeletal network modules: in *purple*, the scapular/forearm-thumb movement module; in *aquamarine*, the flexor/extensor/lumbrical module; in *red*, the wrist/digits 1-2-3 movement module; in *light pink*, the extensor digit minimi module; and in *yellow*, the digits 4–5 movement module.

The skeletal system is as described for the normal newborn/adult. The muscular system comprises 52 muscles and 12 contacts: five fewer muscles and eight more contacts in total than the normal newborn/adult. As in the left T18 UL, the muscular network lacks a small-world organization (Fig 7; Table 6). The T18 right UL musculoskeletal system comprises 86 bones and muscles connected by 180 contacts, and shows a small-world, hierarchical organization. Interestingly, it comprises only 6 modules, which indicates a quantitatively different modular organization than in the normal newborn and in the left T18 UL (68% and 58% similarity between the T18 right *vs*. normal newborn/adult ULs and between the T18 right *vs*. left ULs, respectively).

**Table 6:** Phenotypic modules of the T18 fetus right upper limb identified using AnNA.

***Musculoskeletal Network***			
**#**	**Bones/Cartilages**	**Muscles**	**Complex**
**1**	Hamate, Pisiform, Triquetrum, 4th Metacarpal, 4th Proximal phalanx, 5th Metacarpal, 5th Proximal phalanx	Abductor digiti minimi, Flexor carpi ulnaris, Flexor digiti minimi brevis, Opponens digiti minimi, 2nd Palmar interossei, 3rd Palmar interossei, 4th Dorsal interossei	*Digits 4–5 movement*
**2**	Clavicle, Humerus, Radius, Scapula, Ulna	Abductor pollicis longus, Anconeus, Biceps brachii, Brachialis, Brachioradialis, Coracobrachialis, Deltoid, Extensor carpi ulnaris, Infraspinatus, Latissimus dorsi, Levator scapulae, Palmaris longus, Pectoralis major, Pectoralis minor, Pronator quadratus, Pronator teres, Rhomboid major, Rhomboid minor, Subclavius, Serratus anterior, Subscapularis, Supinator, Supraspinatus, Teres major, Teres minor, Triceps brachii	*Scapular/forearm-thumb movement*
**3**	Capitate, Lunate, Scaphoid, Trapezium, Trapezoid, Ulnar sesamoid, 1st Distal phalanx, 1st Metacarpal, 1st Proximal phalanx, 2nd Metacarpal, 2nd Proximal phalanx, 3rd Metacarpal, 3rd Proximal phalanx	Adductor pollicis, Adductor pollicis accessorius, Extensor carpi radialis brevis, Extensor carpi radialis longus, Extensor pollicis longus, Flexor carpi radialis, Flexor pollicis longus, Musculous interosseous accessorius, 1st Palmar interossei, 1st Dorsal interossei, 2nd Dorsal interossei, 3rd Dorsal interossei	*Wrist/digits 1-2-3 movement*
**4**	2nd Distal phalanx, 2nd Middle phalanx, 3rd Distal phalanx, 3rd Middle phalanx, 4th Distal phalanx, 4th Middle phalanx	Extensor digitorum, Extensor indicis, Flexor digitorum profundus, Flexor digitorum superficialis, 2nd Lumbrical, 3rd Lumbrical, 4th Lumbrical	*Flexor/extensor/lumbrical*
**5**	5th Distal phalanx, 5th Middle phalanx	Extensor digiti minimi	*Extensor digiti minimi*
**6**	Radial sesamoid		*Radial sesamoid*
***Skeletal Network***			
*Same than in the normal adult/newborn* (see SI.Tab 1)			
***Muscular Network***			
**#**	**Muscles**		**Complex**
**1**	Biceps brachii, Coracobrachialis, Deltoid, Pectoralis major		*Biceps/coracodeltopectoral*
**2**	Flexor carpi radialis, Flexor digitorium superficialis, Flexor pollicis longus, Pronator teres		*Flexor*
**3**	Flexor digitorium profundus, 2nd Lumbrical, 3rd Lumbrical, 4th Lumbrical		*Profundus/lumbrical*
**4**	Extensor carpi radialis brevis, Extensor carpi radialis longus		*Extensor carpi radialis*
**5**	Latissimus dorsi, Teres major		*Latissimus/teres major*
**6–41**	*Each other muscle is a 1-muscle module (for a list of all muscles see SI*.*Labels)*		

### The network organization of the T18 fetus right LL

**Fig 8:**
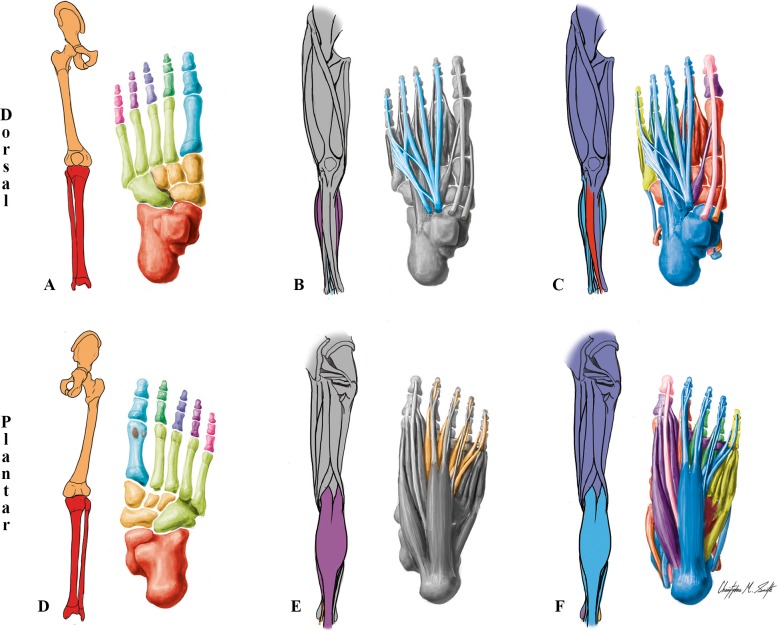
Modules of the T18 cyclopic fetus right LL identified using AnNA. **A** to **C** dorsal (extensor) view; **D** to **F** ventral (flexor) view. A horizontal flip was done with Photoshop, so the modules of this right UL can be more easily compared with those of the left ULs shown in Figs 2, 3 and 5. **A, D)** Skeletal network modules: as in normal newborn (see Fig 4). **B, E)** Muscle network modules: as in left T18 LL (see Fig 5). **C, F)** Musculoskeletal network modules: in *turquoise*, the ankle movement-long flexor/extensor toes module; in *red*, the ankle/digit 2 movement module; in *magenta*, the hip-thigh-leg movement module; in *purple*, the big toe movement module; in *dark green*, the digits 3–4 movement module; in *light green*, the digit 5 movement module; in *pink*, the distal phalanx digit 1 movement module.

As noted above, there are no differences in the network skeletal organization of the T18 left LL, the T18 right LL, and the normal newborn LL (Fig 8; Table 7). The only difference between the muscular modularity of the T18 left and right LLs is that on the right side the popliteus is missing, and therefore there is one less 1-muscle module, in a total of 49 modules (*vs*. 50 in the left side). This muscular difference and other musculoskeletal differences between these limbs (see Section 4) do not result in a difference in the number of musculoskeletal modules (7) and the type of overall musculoskeletal organization (small world, non-hierarchical) between the two limbs, but account for a 17% difference (83% similarity) between these limbs. The similarity between the right *vs*. left T18 LLs is therefore higher than that between the right T18 *vs*. normal newborn LLs (74%).

**Table 7:** Phenotypic modules in the right lower limb of the T18 fetus.

***Musculoskeletal Network***			
**#**	**Bones/Cartilages**	**Muscles**	**Complex**
**1**	Cuboid, Intermediate cuneiform, Lateral cuneiform, Medial cuneiform, Navicular, 1st Metatarsal, 2nd Metatarsal, 2nd Proximal phalanx	Fibularis longus, Tibialis anterior, Tiblialis posterior, 1st Dorsal interossei, 1st Lumbrical, 2nd Dorsal interossei	*Ankle/digit 2 movement*
**2**	Femur, Ischium, Pubis, Ilium, Tibia, Patella	Adductor brevis, Adductor longus, Adductor magnus, Gemellus inferior, Gemellus superior, Gluteus maximus, Gluteus medius, Gluteus minimus, Gracilis, Iliopsoas, Obturator externus, Obturator internus, Pectineus, Piriformis, Quadratus femoris, Sartorius, Tensor fasciae latae, Biceps femoris, Rectus femoris, Semimembranosus, Semitendinosus, Vastus intermedius, Vastus lateralis, Vastus medialis	*Hip-thigh-leg movement*
**3**	2nd Distal phalanx, 2nd Middle phalanx, 3rd Distal phalanx, 3rd Middle phalanx, 4th Distal phalanx, 4th Middle phalanx, Calcaneus, Fibula, Talus	Extensor digitorum brevis, Extensor digitorum longus, Flexor digitorum longus, Flexor digitorum brevis, Quadratus plantae, 4th Lumbrical, Fibularis tertius, Fibularis brevis, Gastrocnemius, Plantaris, Soleus,	*Ankle movement-long flexor/extensor toes*
**4**	Lateral sesamoid, Medial sesamoid, 1st Proximal phalanx	Abductor hallucis, Adductor hallucis, Extensor hallucis brevis, Flexor hallucis brevis	*Big toe movement*
**5**	3rd Metatarsal, 3rd Proximal phalanx, 4th Metatarsal, 4th Proximal phalanx	1st Plantar interossei, 2nd Lumbrical, 3rd Dorsal interossei, 2nd Plantar interossei, 3rd Lumbrical, 4th Dorsal interossei	*Digits 3–4 movement*
**6**	5th Metatarsal, 5th Proximal phalanx, 5th Distal phalanx, 5th Middle phalanx	Abductor digiti minimi, Flexor digiti minimi brevis, 3rd Plantar interossei,	*Digit 5 movement*
**7**	1st Distal phalanx	Extensor hallucis longus, Flexor hallucis longus	*Distal phalanx digit 1 movement*
***Skeletal Network***			
**#**	**Bones/Cartilages**		**Complex**
**1–11**	*Same than in the normal newborn* (see SI.Tab. 3)		
***Muscular Network***			
**#**	**Muscles**		**Complex**
**1–49**	*Same than in the left T18 LL with major 3 muscle modules and each other muscle being a single module (see SI*.*Tab 5)*, *the only difference being that in the right side the total number of muscle modules is 49*, *not 50*, *because the popliteus is missing (for a list of all muscles see SI*.*Labels)*		

## Discussion

### Normal phenotype of ULs

This is the first study using AnNA for the LLs of any tetrapod species, using a wide range of AnNA methods to acquire a vast amount of quantitative data for both the LLs and ULs in order to offer a comprehensive discussion of, and comparison between, these limbs. The S1–S24 Figs display a schematic summary of the anatomical networks of all limbs compared in the present work. These type of schemes are extremely useful to easily visualize and compare the overall connectivity patterns and network organization of each system: skeletal, muscular or musculoskeletal. Fig 1 provides an example of this in the skeletal networks of the normal adult UL *vs*. LL. For example, these two networks can be easily compared with, and thus used to discuss, Shubin & Alberch's model of limb skeletal morphogenesis [52], which is commonly used in textbooks and also specialized papers as a basis to study limb evolution and development [53].

According to this model, limb development involves the differentiation of *de novo* condensation (humerus/femur), which bifurcates to give rise to the ulna/fibula and radius/tibia cartilages. Importantly, the model assumes that the postaxial (ulnar/fibular) side of the limb is where most differentiation events take place ("postaxial dominance"). For instance, in the UL, the condensation, bifurcation, and segmentation of the ulna produces the formation of cartilages of the primary axis, such as the triquetrum ('ulnare' in amphibians) and lunate ('intermedium' in amphibians), in a proximodistal sequence. Then the condensation and bifurcation of the lunate gives rise to two proximal centralia, each then giving rise to each of the two distal centralia (N.B., in normal human development the central bone becomes fused with other carpal bones before birth). In turn, condensation, bifurcation and segmentation of the triquetrum will give rise to the digital arch in a posteroanterior (ulno-radial in human anatomy) sequence, to the hamate and digit 4 (this digit is the primary axis of the phalangeal region), to the capitate and digit 3, the trapezoid and digit 2, and trapezium and digit 1. The hamate, capitate, trapezoid and trapezium correspond to the '4th, 3rd, 2nd and 1st distal carpals' of amphibians, so in this model digit 5 is often seen as a *de novo* condensation. Regarding the scaphoid ('radiale' in amphibians), it segments from the radius, i.e. in this 'postaxial dominance' model the preaxial bones normally segment, but do not bifurcate.

In view of this Shubin & Alberch model, one would thus predict that, at least in some stages of the normal tetrapod development, the postaxial (ulnar) elements would in general display a higher connectivity to other elements (bifurcation and segmentation) than would the preaxial (radial) elements, in which there is only segmentation (i.e. radius primarily articulates distally only with scaphoid, and scaphoid not being part of the central series of carpal bones nor of the digital arch group). However, the normal adult/newborn skeletal network organization shown in Fig 1 actually seems to have a preaxial, instead of a postaxial, dominance in terms of connectivity patterns, with more posterior (ulnar) bones being more peripherical within the whole network. For instance, the ulna itself does not articulate with any carpal bone, while the radius articulates distally with the scaphoid, but also with the lunate. Also, the triquetrum articulates with two cartilaginous carpals (lunate and hamate; the pisiform being a sesamoid bone), while the scaphoid articulates with four (trapezium, trapezoid, capitate and lunate).

The overall adult/newborn skeletal network organization of the LL seems to be more symmetrical than the one of the UL (Fig 1). That is, there is no clear preaxial connectivity dominance as in the UL (e.g. both the tibia and fibula articulate with the talus, and lateral-fibular-tarsal bones such as the cuboid and lateral cuneiform are highly connected with other bones in the network), but there is no clear postaxial connectivity dominance either. However, three points should be made. Firstly, in adults of early tetrapod taxa the ulna articulated with the triquetrum (that is why anatomists named this bone 'ulnare' in those tetrapods [54]. Therefore, some of the ancestral adult connectivity patterns that would mirror the developmental relationships predicted by Shubin & Alberch's model were surely lost during tetrapod evolutionary history. Secondly, there are however connectivity patterns in early tetrapods and closely related sarcopterygian fish that do not mirror the ontogenetic relationships predicted in that model. For instance, in rhizodontid fish the radius articulates with more radials than does the ulna, similarly to what happens in the adult human/mammalian skeletal network [55]. In fact, the third point is that although Shubin & Alberch's model continues to be often used in specialized papers and particularly in textbooks, as noted above, at least some of the specific points of this model have been contradicted by more recent developmental studies, and necessarily need to be polished (for a detailed review and discussion of these specific points see [56]). Including AnNA in future embryological and developmental experimental works might help to better understand the details of, and changes in the patterns of connectivity during, skeletal morphogenesis in the UL and LL of humans and other tetrapods.

Another interesting aspect of the analysis of the skeletal network of the normal human/newborn UL is that metacarpal 1 behaves, in a network context, exactly as does the proximal phalanx of each of the other digits, being included together with the proximal and distal thumb phalanges into a digit1/metacarpal 1 skeletal module, while the four modules including the other four digits include only phalanges (Figs 2 and 3; Tables 1 and 2). As the same five modules are also seen in the skeletal organization of the foot, this could be used as an argument to support the view of those authors arguing that the bones that are usually designated as metacarpal 1 and metatarsal 1 actually correspond to the proximal phalanges of the thumb and big toe, respectively. That is, that these digits have three phalanges each, as do the other digits, and that the true metatarsal 1 and metacarpal 1 are actually missing (see, e.g. [57,58]). In contrast to metacarpal 1, all other metacarpals are included, together with all carpals, in the *wrist/metacarpals 2–5* module (Figs 1 and 2; Table 1). The other three modules of the nine-module skeletal network of the normal UL are the *girdle/arm/forearm* module including the clavicle, scapula, humerus, radius and ulna, a module including only the radial sesamoid, and a module including exclusively the medial sesamoid (these two sesamoid bones are embedded in soft tissues, and do not have major articulations with other bones).

By adding muscles to these skeletal modules, the clear proximodistal modular separation seen in the skeleton (girdle/arm/forearm *vs*. wrist/metacarpals 2–5 *vs*. each digit *vs*. each sesamoid) becomes more faint. For instance, some musculoskeletal modules of the normal UL extend from the body midline to the distal phalanx of the thumb (*arm-forearm-thumb movement* module, including latissimus dorsi and flexor and extensor pollicis longus). The grouping of the thumb and digits 2 and 3 in the *digits 1-2-3 movement* musculoskeletal module (Fig 2; Table 1) is interesting because the thumb has a developmental and evolutionary history that is markedly different to that of other digits (e.g. peculiar pattern of *Shh* expression [59] and morphofunctional evolution in primates [28]. In addition, the existence of a *digits 4–5 movement* musculoskeletal module in humans is also interesting, and unexpected functionally and evolutionary, because digit 4 is the first to form developmentally in our species while digit 5 forms later (often starting to form only after digit 3, and even 2, in mice and humans) and is functionally different from the other fingers (e.g. contributing to the full opposition of our hand, together with the thumb) [57,60]. The existence of a *superficial flexor/extensor* module, including numerous muscles as well as bones from digits 2, 3, 4 and 5, as well as of the *arm-forearm-thumb movement* module including muscles that attach onto the thumb but not onto other digits, do reflect the clear developmental separation of the thumb from the other digits in tetrapods in general, and its functional separation from other digits during primate and human evolutionary history in particular.

The three other musculoskeletal modules of the normal UL are the *profundus/lumbrical* module that is the only UL musculoskeletal module exclusively formed by muscles, the *clavicle movement* module that includes the clavicle and the only UL muscles (deltoid, pectoralis major and subclavius) attached to it, and the *scapular* module which includes the scapula and muscles attached to it (Fig 2; Table 1). Importantly, the overall musculoskeletal network of the normal adult/newborn UL is more similar to what would be expected based on knowledge of functional groupings (47% similarity) than based on developmental groupings (41%). That is, the whole musculoskeletal network organization of the normal UL seems to reflect function slightly more than development (*S4 Results*).

### Abnormal phenotype of T18 ULs

In recent works we described and compared in some detail two models that reflect two very different ways of viewing birth defects [46,61]. Therefore, here we will just provide a short introduction to them; for more details readers should refer to those two works, and particularly to the original papers [62,63]. In short, Alberch's ill-named theory "the logic of monsters" (*LoMo*) [62,63] argued that teratologies are forms that lack adaptive function but that normally preserve structural order, being based on an "internalist" developmental framework. That is, due to strong (internal) developmental constraints and thus a limited set of possible phenotypic outcomes, a teratological form has to follow the rules that pertain to the normal developmental mechanisms available. Alberch thus suggested that the study of birth defects can be particularly useful to better understand normal development, thus coming back to a view that was often followed by researchers between the 11th and 17th centuries but then mainly became abandoned—and often ridiculed—by various researchers in the 18th century [64].

For instance, the LoMo has very different assumptions and predictions than models that have been more accepted by pathologists and comparative anatomists in the last decades, such as the "lack of homeostasis" model of Shapiro [63], which tend to see birth defects as more random, chaotic phenotypic features. In fact, the only major point in which the two models agree is that the developmental processes that will be the most often and seriously affected are those that are more unstable (leading to variations) in the normal population. An illustrative example, predicted by both the LoMo and "lack of homeostasis" models, is that a very common human variation (polymorphism), the absence of palmaris longus muscle seen in c.15-20% of normal population, is often seen amplified in humans with severe congenital malformations: muscle absent in 74% (105) of 141 defective upper limbs reviewed in Smith et al. [49]. However, the "lack of homeostasis" model predicts this outcome because it assumes a generalized decreased developmental and physiological homeostasis, while the LoMo predicts it because it assumes a logical parallel between variant and defective development, due to strict developmental constraints. That is, while the "lack of homeostasis" model argues that defects are in general more random and disorganized due to a general disturbance of homeostasis, the LoMo predicts that defects are more "logical" and "constrained" because constraints are in general still kept intact by internal homeostasis. Therefore, contrary to the former model, the LoMo predicts that congenital malformations and plastic variations found in a certain taxon often also mirror features that are consistently found in the normal phenotype of individuals of other taxa. This prediction has been supported by studies showing that the existence of similar patterns of intra-specific diversity in a taxon (plasticity) and inter-specific diversity in different taxa is usually the result of similar developmental mechanisms [65].

AnNA is a powerful tool to contribute to such discussions, which have important medical, developmental and evolutionary implications. This is because it is able to provide quantitative data to specifically examine if there is an "internal logic" (e.g. of connectivity patterns and network organization) in cases of individuals with birth defects, or if the patterns observed in these individuals reflect instead a more chaotic, random disarray of defects, such as predicted for instance in the "lack of homeostasis" model. For example, the LoMo explains why some abnormal fusion of muscles seen in the limbs of the T18 fetus studied for the present work (e.g. flexor pollicis brevis and opponens pollicis; extensor pollicis brevis and abductor pollicis longus [49]) is also present in normal variants of the human population and in the wild type phenotype of various non-human primates [28].

Most examples regarding the gross anatomy and network organization of the muscular system of the left T18 UL support the LoMo. In fact, one of the more striking results of our AnNA, which clearly illustrates the quantitative power of this methodology, is that the overall musculoskeletal network organization of the T18 left UL is actually more logical than that of the normal newborn/adult UL itself. That is, it is 48% and 55% similar to the developmental and functional groupings, respectively; while the numbers of the normal adult/newborn limb are 41% and 47%, respectively (*S4 Results*). These are the kind of empirical results that can seem surprising, but that provide a showcase of the possibilities and quantitative value of AnNA because many authors, based on a combination of *a priori* expectations and the use of rather subjective methods, argue that birth defects are just rather chaotic, random, and often unpredictable, as explained above. However, as seen in the case of the left T18 UL, in at least some cases birth defects can actually can be more predictable than the normal phenotype itself, thus providing important clues to better understand both normal and abnormal development. Based on alternative models such as the LoMo that predict that there is often a "logic" in abnormal development due to strict developmental constraints, these quantitative results become easier to understand and to frame in a broader developmental and evolutionary context.

In fact, the major surprise is that the T18 left UL musculoskeletal network is more predictable than the normal phenotype in not only a developmental, but also a purely functional, context (*S4 Results*), because even the LoMo assumes that birth defects should in general not be adaptive/functional. Of course, in the context of the whole body one cannot argue that this T18 fetus was more "functional" than a normal fetus, if not it would not have died during the fetal stage. However, this T18 fetus clearly did not die because of the specific phenotypic defects of his left UL, neither. Therefore, the *a priori* expectations that all systems of all body regions would be affected in a similar, non-functional, random/chaotic way due to a "lack of homeostasis" do not stand in view of the empirical AnNA results obtained here, and particularly of the similarity percentages referred to just above. However, there are of course a few cases in which the network modularity of the left T18 UL seems to be less logical, functionally, than that of the normal phenotype. For instance, the *clavicle movement/biceps* musculoskeletal module of the left T18 UL seems less logical functionally than the *clavicle movement* musculoskeletal module of the normal UL because the biceps brachii has no relation to the movements of the clavicle. Instead, this peculiar T18 musculoskeletal module is due to an abnormal—and very likely non-functional—blending between the biceps brachii and the pectoralis major, on both sides of the T18 fetus.

Such examples of abnormal, unpredictable, and/or seemingly non-functional configurations are however much more common in the right T18 UL. They include for instance the integration of extensor muscles with the flexor digitorum profundus and lumbricals, resulting into a *flexor/extensor/lumbrical* musculoskeletal module, and the presence of a separate *extensor digiti minimi* musculoskeletal module (Fig 7; Table 6). This is quantitatively reflected by the fact that the similarity values between the phenotype of the right T18 UL and the functional and developmental groupings shown in *S2 Methods* are much lower than those for the left T18 UL, being also lower than those for the lower phenotype: 35% (developmental) and 46% (functional) (vs. 48% and 55% in the left T18 UL, and 41% and 47% in the normal phenotype, respectively; *S4 Results*). Another interesting quantitative result is that the overall musculoskeletal networks of the right and left T18 ULs are each more similar to that of the normal UL (68% and 71% similarity respectively) than to each other (58% similarity). That is, the overall network organization of the T18 ULs is markedly asymmetrical.

### Normal phenotype of LLs

The 11 skeletal network modules of the normal adult LL are shown in Figs 1 and 3, and Table 2. In terms of the individual bones they include, they are somewhat similar to the nine skeletal network modules of the normal adult UL, including the placement of metatarsal 1 in the *digit 1/metatarsal 1* module. The only difference is that in the normal adult UL all carpals are included in a single module with metacarpals 2-3-4-5, while in the normal adult UL the tarsals are divided into three modules: the navicular and cuneiforms are grouped with metatarsals 2 and 3 in the *tarsals/metatarsals 2–3* module, the cuboid and metatarsals 4 and 5 form the *cuboid/metatarsals 4–5* module, and the calcaneus and talus form the *leg/proximal ankle* module together with the tibia and fibula.

While in the normal adult UL each muscle forms a single-muscle module, in the normal adult LL there is a flexor *longus/plantae/lumbrical* muscle module formed by the lumbricals 1, 2, 3 and 4, the flexor digitorum longus muscle that provides the physical place of origin for these four small muscles, and the quadratus plantae that is connected to this latter long muscle (Fig 3; Table 2). This is an interesting difference between the UL and LL, because in the normal adult UL the lumbricals are also originated from the tendons of the flexor digitorum profundus; it thus seems that the addition of the quadratus plantae is crucial for the definition of the *longus/plantae/lumbrical* muscle module of the LL. The only other muscle module of the normal adult LL including more than one muscle is the *extensor longus/fibularis* module, including the extensor digitorum longus and a small muscle, the fibularis tertius, that in the normal phenotype usually originates the tendon of this long muscle to digit 5 (Fig 3; Table 2).

Regarding the nine musculoskeletal modules of the normal adult LL, the *ankle/digit 2 movement* module is very different from any of the UL musculoskeletal modules, as in the UL the musculoskeletal module including many carpal bones is a thumb movement module, while the corresponding LL module is mainly related to muscles moving digit 2 and the foot as a whole (Fig 3; Table 2). The *hip-thigh-leg movement* module includes all pelvic and thigh muscles as well as a zeugopod (leg) bone, the tibia. Therefore, it basically combines aspects of the UL *scapular* module and *arm-forearm-thumb movement* module of the UL. However, there are major differences relative to the UL, as this *hip-thigh-leg movement* includes only a zeugopod bone, and does not include any zeugopod muscle nor any muscle related to digit 1 (i.e. big toe, in the foot) movement, for instance. The bones of the *long flexor/extensor toes* LL musculoskeletal module correspond topologically to the ones included in the *superficial flexor/extensor* module of the UL. However, in terms of muscles this LL module corresponds to a mix between the muscles included in both the *profundus/lumbrical* module and the *superficial flexor/extensor* module of the UL, because it also includes the flexor digitorum longus and one of the four lumbricals (the 4th) that is connected to the this long muscle.

The *big toe movement* musculoskeletal module of the normal adult LL is somewhat similar to the *thumb movement* module of the adult UL in terms of the muscles it includes, the major difference being that there is no opponens hallucis in the human foot. Moreover, this LL module includes only the proximal phalanx and sesamoid bones of the big toe, while the UL module include the thumb proximal phalanx and sesamoid bones but also metacarpal 1 and numerous carpals. Contrarily to the adult UL, in the adult LL there are three separate musculoskeletal modules for the movements of digits 3, 4, and 5. Furthermore, in the adult UL the long flexor and extensor muscles of the thumb are included in the *arm-forearm-thumb movement* module, while in the LL the long flexor and extensor muscles of the big toe are included in a separate module, the *distal phalanx digit 1 movement* module. This is interesting, because one would expect the peculiarities related to the special human thumb to be associated with a higher parcellation (i.e. more modules) allowing higher freedom of movements in the human hand. However, it should be pointed out that phylogenetic constraints seem to be extremely important for the patterns seen in the adult human UL and LL [50]. As our ape ancestors also used the LLs to move on the trees (e.g. grasp tree branches, that is why apes are often called 'quadrumana'), they also had a highly mobile big toe, as extant apes do [66], and the human LL anatomical network organization revealed by AnNA might reflect that evolutionary history. Therefore, further comparative studies of other primates are needed, for instance to analyze whether there is effectively a similar modular configuration of the big toe in the LL of other primates and particularly apes.

The other LL musculoskeletal module is the *ankle movement* module, which includes the fibula, calcaneus, talus and the three superficial posterior leg muscles (gastrocnemius, plantaris and soleus, all innervated by the tibial nerve), and interestingly also a lateral leg muscle innervated by the superficial fibular nerve (fibularis brevis). This is an illustrative example of how muscles of two developmental groups become integrated in a single musculoskeletal functional module. In fact, our quantitative AnNA reveals that the whole network organization of the normal adult LL is significantly more similar to what would be predicted based on functional groupings (45%) than based on developmental groupings (36%) (*S4 Results*).

Focusing now on the normal newborn LL (Fig 4; Table 3), the only skeletal difference in terms of patterns of connectivity between this LL *vs*. the normal adult LL concerns the fusion of the ischium, pubis and ilium into a single hip bone during postnatal development. This single difference leads to a more cohesive, hierarchical skeletal organization of the LL in the adult, resulting in changes in both the skeletal (88% similarity to newborn) and musculoskeletal (86% similarity to newborn) networks, although the number and overall configuration of the muscles remain exactly the same (*S3 Results*). Namely, in terms of skeletal modules, instead of the adult *tarsals/metatarsals 2–3* module and the *cuboid/metatarsals 4–5* module of the adult LL, the newborn LL has a *tarsals* module and a *cuboid/metatarsals 2-3-4-5* module because metatarsals 2 and 3 are included in this latter module. The differences between the network organization of the newborn and adult LLs emphasize the sensibility of AnNA. In a way, these differences bring to mind the chaos theory and butterfly effect of physics, which postulate that a small change in one state of part of a system can have significant effects within seemingly unrelated parts of the system (*"the flap of a butterfly wing in Brazil can cause a Tornado in Texas"* [67]). That is, the postnatal fusion between the ilium, ischium and pubis does not affect the modularity organization of the surrounding (proximal) skeletal structures of the LL, but instead causes the distal *metacarpals 2 and 3* to move from the *cuboid/metatarsals 2-3-4-5* skeletal module of the newborn to the *tarsals/metatarsals 2–3* skeletal module of the adult. This is even more striking when one takes into account the mainly non-chaotic, *ordered* nature of the changes displayed by abnormal limb phenotypes, and particularly the fact that these changes during normal developmental lead to a musculoskeletal network organization of the normal adult LL that is remarkably more similar to that of the left T18 LL than to that of the normal newborn LL itself.

The major differences regarding the musculoskeletal network of the normal newborn *vs*. adult LLs concern the *hip-thigh movement* module and the *leg/ankle movement* module of the newborn. That is, in the newborn the tibia, patella and many muscles that move the leg are included in a *leg/ankle movement* module (which is thus different from the adult *ankle movement* module), while during postnatal development they became included into the *hip-thigh-leg movement* module (which thus contrasts with the newborn *hip-thigh movement* module). That is, the postnatal fusion of the ischium, ilium and pubis into a single hip bone results in a more cohesive pelvis that then becomes integrated, within the whole musculoskeletal network, into a larger *hip-thigh-leg movement* that also includes bones of, and muscles that move the, leg. As expected, the newborn overall musculoskeletal LL organization configuration is slightly more similar to what is expected based on developmental groupings (38%) than is the adult overall musculoskeletal LL organization (36%) (*S4 Results*).

### Abnormal phenotype of T18 LLs

For those researchers defending a more chaotic/random view of birth defects, one of the more surprising results of our AnNA will probably be the fact that in terms of musculoskeletal network organization, the left T18 LL is more similar to the normal adult LL (89%) than is the normal newborn LL itself (86%), as noted above (*S3 Results*). How can it be that a normal newborn LL, that will give rise to a normal adult LL, is more different in its overall musculoskeletal network organization from the adult LL, than is the left LL of a fetus with a condition as severe as T18, which usually results in individuals dying before birth [49,68]? This example provides further support for LoMo, because it emphasizes that the network organization of the T18 fetus is far from being chaotic, stressing that the study of birth defects, and the use of AnNA, can effectively open various new lines of research with medical implications. Specifically, more developmental studies using AnNA should be done to examine the changes of network organization in more ontogenetic stages of both normal and abnormal development, ideally combined with molecular and experimental developmental studies, in order to understand the links between normal and pathological development in a more detailed, comprehensive way.

The new data provided in the present study allows us to pave the way for such studies. For instance, a detailed analysis of the results reveals that a major difference in the muscular system of the T18 LL *vs*. the normal newborn LL is the fusion of the gastrocnemius and soleus in the left T18 LL. These two muscles thus form a module together, instead of constituting each its own module, as they do in the normal newborn and adult LLs, and therefore there is slightly more integration (i.e. less parcellation) in the muscular network of the T18 left LL than in the normal newborn/adult LL. A similar example concerns the musculoskeletal system, in which the left T18 LL has a *digits 3–4 movement* module, while in the normal newborn/adult there is one *digit 3 movement* module and one *digit 4 movement* module. Another major difference between the left T18 LL and the normal newborn LL is that the calcaneus, fibula and talus and the muscles fibularis brevis, gastrocnemius, plantaris and soleus are not included in the *leg/ankle movement* musculoskeletal module, as they are in the normal newborn. Instead, they are integrated in the *ankle/digit 2 movement* musculoskeletal module, which actually seems theoretically more logical functionally, because it means that in the left T18 LL almost all muscles working on the ankle are grouped in this latter module.

In turn, in the left T18 LL the tibia and patella and the major flexors (semimembranosus, semitendinosus, biceps femoris) and extensors (rectus femoris and vastus intermedius, lateralis and medialis), as well as the popliteus, instead of being part of the *ankle/digit 2 movement* musculoskeletal module (as seen in the newborn), are part of the *hip-thigh-leg movement* module. This results also in less parcellation in the abnormal limb; together with the differences mentioned above, in total the left T18 LL has only seven musculoskeletal modules, *vs*. nine in the normal adult/newborn LL. Interestingly, this *hip-thigh-leg movement* module of the left T18 LL is very similar to the one of the adult normal LL, and very different from the pattern seen in the normal newborn LL. It is therefore this feature that mainly contributes to the *pattern* described above, i.e. that the whole musculoskeletal network organization of the normal adult LL is more similar to that of the left T18 LL than to that of the normal newborn LL. Knowing this pattern can thus now help with studying the processes that lead to this pattern. For instance, this pattern might be the result of abnormal developmental acceleration in the left T18 LL, leading to the display of connectivity patterns (in this fetal limb) that are normally only acquired during postnatal development. As noted above, detailed developmental and experimental studies are thus needed to clarify the mechanisms involved in abnormal limb development, including testing this specific acceleration hypothesis.

Regarding the differences between right *vs*. left T18 LLs, these concern only one difference in muscular modularity (popliteus missing on right side, so there is one less 1-muscle module, and the popliteus is therefore not included in the *hip-thigh-leg movement* musculoskeletal module), and the following two major differences in musculoskeletal modularity. The first is that on the right side there is a *ankle movement-long flexor/extensor toes* module (Fig 8; Table 7). This difference is related to the fact that the fibularis tertius was integrated in this module because on the right side this muscle has a separate muscle belly originating from the fibula (i.e. it is not simply a mainly tendinous structure originating from the tendon of the extensor digitorum longus to digit 5). That is, because of this abnormal connection between the fibula and fibularis tertius, this latter muscle became integrated in the musculoskeletal module that includes the fibula. The overall comparison of all LLs included in the present work thus indicates that the *ankle movement* musculoskeletal module is particularly flexible and unstable developmentally. It is defined as a separate module in the normal adult LL, it is part of a larger module in the normal newborn LL together with movers of the leg, providing an illustrative example of a postnatal change leading to more parcellation (i.e. more modules). In turn, it is associated with the *ankle/digit 2 movement* module in the left T18 LL and with the *long flexor-extensor toes* module in the right T18 LL.

The second major musculoskeletal difference between the right and left T18 LLs is that the *digit 5 movement* module seen on the right side includes the middle and distal phalanges of digit 5. This configuration makes sense in theory, because in both the right and left sides of the T18 fetus the flexor digitorum brevis no longer goes to digit 5, and moreover on the right side this also happens to the extensor digitorum longus (i.e. it also lacks a tendon to digit 5). This is a good example of how some anatomical changes occurring during both normal and abnormal development may be "buffered". It is only due to the additional absence of the tendon of the extensor digitorum longus to digit 5 seen on the right T18 LL—and thus the accumulation of both defects in this limb—that the overall network pattern is changed in this latter limb. This example also illustrates how the absence of structures in abnormal development, in this case of the tendons of the extensor digitorum longus and flexor digitorum brevis to digit 5, often leads to more integration or less parcellation (see also [46], for similar examples, but concerning the head).

In summary, the left T18 LL is more similar to the normal phenotype, its overall musculoskeletal organization being more similar to that of the normal adult LL, than is the organization of the normal newborn LL. In contrast, the right T18 LL is more modified, as expected by the greatest number of phenotypic defects [49]. Contrary to the UL, there is less similarity to functional and developmental groupings in both the left (0.44 and 0.34, respectively) and right (0.43 and 0.34, respectively) T18 LLs than in both the normal newborn (0.45 and 0.38, respectively) and adult (0.45 and 0.36, respectively) LLs (N.B., within ULs, the highest similarities are seen in the left T18 UL; *S4 Results*). However, the major changes that lead to the formation of the peculiar musculoskeletal modules of the T18 right LL (fibularis tertius being separate muscle, and extensor digitorum longus and flexor digitorum brevis missing tendons to digit 5) are often seen in human variations and in the normal phenotype of other animals [49], further providing support for the LoMo. Therefore, it is likely that the AnNA of the limbs of other animals will reveal that these modules uniquely found in the right T18 LL within the present study are in fact not peculiar at all within tetrapods as a whole.

### Comparison of ULs and LLs and notes on UL-LL serial homology *vs*. homoplasy

The overall comparison between all the limbs studied for the present work reveals several aspects that open further avenues of research. One is that the musculoskeletal similarity between the left and right T18 ULs *vs*. the normal newborn ULs is 71% and 68%, respectively, while that of the left and right T18 LLs *vs*. the normal newborn LLs is 79% and 74%, respectively (*S3 Results*). So, in the T18 fetus the LLs are clearly less defective than the ULs, and the left side less defective than the right side, both in terms of their gross anatomy and in terms of their intricate musculoskeletal network organization. It is often suggested in the literature about birth defects that the LLs have less defects than the ULs, but it is not clear if that suggestion reflects the reality of abnormal development, or instead a bias due to the fact that most studies tend to focus more on the ULs (for a recent overview, see [49]). The results of a recent review including data obtained exclusively in studies that described defects in both the ULs and LLs of human individuals with limb birth defects (thus avoiding such biases) suggest that the ULs tend effectively to have more gross anatomical defects than the LLs [61]. Within 316 defects compiled in studies including the head and ULs and LLs, the proportion of UL defects (158, or 50%) was substantially higher than that of LL defects (64, or 20%) (and of head defects: 94, or 30%). However, the present study is the first providing empirical data showing that this pattern is also seen in the overall network modularity of the limbs, at least in the case of the T18 fetus.

Interestingly, the comparisons between all the limbs included in our quantitative analysis contradicts the commonly accepted idea that birth defects often lead to a lower integration (i.e. more parcellation) of bones and muscles [33]. This is because within the 12 direct comparisons of the number of skeletal, muscular and musculoskeletal modules between the normal newborn *vs*. left and right T18 ULs and LLs, only in one case there are more T18 modules: the number of musculoskeletal modules in the normal UL is 7, vs. 9 in the left T18 UL. There is a similar number of skeletal modules in the normal newborn and T18 left and right ULs (9 modules) and LLs (10 modules each). In the seven remaining cases there are in fact less modules in T18: muscular modules are 38 in left and 41 in right T18 ULs *vs*. 57 in normal newborn UL, and 50 in left and 49 in right T18 LLs *vs*. 51 in normal newborn UL; musculoskeletal modules are 6 for right T18 UL *vs*. 7 for normal newborn UL, and 7 in left and 7 in right T18 LLs vs. 9 in normal newborn LL.

As noted in before, there is a 93% similarity between the skeletal systems of the normal adult UL *vs*. LL, but a profound difference (27% similarity) between their whole musculoskeletal systems (*S3 Results*). It is therefore striking to see that the musculoskeletal similarity between the T18 left UL and LL (33%) is actually higher than that between the normal adult UL *vs*. LL, particularly taking into account that the skeletal similarities are 27% in both cases (normal adult UL vs. LL, and T18 left UL *vs*. LL). That is, by adding muscles to the network analysis, the overall similarity between the abnormal UL and LL of the T18 fetus becomes actually higher than that of the normal UL and LL themselves. This supports the LoMo theory, and makes sense when one takes into account what was stated in Section 3.2 about the musculoskeletal system of the T18 left UL being more "logical/predictable" developmentally (47%) than the normal adult UL itself (40%). That is, according to the LoMo, defective organs still display a "logical" pattern due to strong developmental constraints—in some cases even more "logical" than the normal phenotype—,as is the case of the left T18 UL. Therefore, because the developmental mechanisms involved in the normal morphogenesis of both the UL and LL are so similar, particularly their distal regions, and the left T18 UL seems to be particularly constrained developmentally, it does make sense that the whole musculoskeletal network organization of the left T18 UL and LL is actually more similar than the one seen in the UL and LL of normal adults, in which, apart from many other factors, strong developmental constraints are at play.

In other words, by displaying a higher/more direct link between developmental mechanisms/constraints and the phenotypic outcome, the left T18 UL reflects better the derived developmental mechanisms that it shares with the left T18 LL than does the normal adult UL and LL, where these links are lower/less direct. This is thus an illustrative example of how the study of the abnormal can in fact reveal important insights on the development of the normal [69]. This does not happen on the right side of the T18 fetus, because the UL and LL of that side have more abnormalities than the left ones, probably because they broke some developmental constraints that were not broken in the normal newborn/adult limbs and in the left T18 limbs. Therefore, the similarity between the musculoskeletal systems of the T18 right UL *vs*. LL (22%) is significantly lower than that between the normal adult UL *vs*. LL (27%) and between the left T18 UL *vs*. LL (33%) (*S3 Results*). Another related point of our results that illustrates the quantitative power of AnNA is that both the skeletal and musculoskeletal network organization of the normal adult UL are more similar to developmental (41%) and functional (47%) groupings than is that of the normal adult LL (36% and 45% respectively: *S4 Results*).

A major difference between normal and abnormal limbs is that the T18 limbs display a left-right asymmetry, while phenotypically normal limbs are obviously usually symmetric. However, this asymmetry is clear not as high and chaotic as predicted by the "lack of homeostasis" hypothesis, with each limb displaying a series of random, peculiar features. Regarding the ULs, the overall musculoskeletal network similarity between the left and right T18 ULs (58%) is effectively lower than that between each of these limbs and the normal adult/newborn UL (68% and 71% between normal UL and right and left T18 ULs, respectively). This reveals a marked asymmetry, as noted above. However, the overall musculoskeletal network similarity between the left and right T18 LLs (83%) is actually higher than that between each the of these limbs and the normal newborn LL (74% and 79% between normal newborn LL and right and left T18 LLs, respectively) (*S3 Results*). Furthermore, the musculoskeletal system of both T18 left and right LLs has a small world organization, and that of both the ULs of this fetus has also a small-world organization, as well as a hierarchical organization, thus pointing to a natural deviation from randomness. In fact, a recent review on muscle birth defects provided further support for the LoMo [61]: within the total 1540 human muscle defects compiled for that review, the vast majority (1044, i.e. 68%) are found in *both* the left (522) and right (522) sides of a same individual, while only 496 (32%) are found in a single side. The fact that a left-right symmetry is usually kept in individuals with severe congenital malformations, not only in non-defective structures but even in structures that are extremely defective themselves, does not support the "lack of homeostasis" model, which predicts a more random, and thus asymmetrical, distribution of defects.

In this regard, the overall comparison of all the four limbs of the T18 fetus studied for the present work provides a particularly illustrative case study supporting the LoMo. This is because, despite the several malformations displayed by this fetus, there is an overall coherent, "logical" and predictable pattern in all four limbs (Figs 6 and 8). In both ULs the extensor digitorum tendon to digit 5 is missing, and moreover the tendon to digit 5 of the extensor digitorum longus (which topologically corresponds to the extensor digitorum in the LL) is also missing on the right foot. Furthermore, in both ULs the flexor digitorum superficialis is missing the tendon to digit 5, and in both LLs the flexor digitorum brevis (which topologically corresponds to the extensor digitorum in the LL) is also missing a tendon to this digit. In addition, the tendons to digit 5 of all these muscles are often missing in both variations of the normal human population and in the normal phenotype of various non-human taxa [70], as predicted by the LoMo.

Importantly, all these similarities between the defects of the dorsal (extensor) and ventral (flexor) muscle masses of the ULs and LLs are seen only in the zeugopods and autopods, supporting the idea that these similarities are due to a very strong developmental link resulting from a derived, homoplasic co-option of similar genes to form these muscle masses on the distal—and evolutionarily new/derived—portion of the four limbs [50,71]. In fact, the dorso-ventral symmetry and upper-lower limb similarity are seemingly even more constrained than the left-right symmetry in some cases. For example, in the T18 right leg and forearm short extensors that normally go to digit 2 (extensor digitorum brevis bundle going to digit 2 and extensor indicis, respectively) go instead to digits 2 and 3, while on the left side they have a normal insertion to digit 2 only. In striking contrast, within the numerous defects found in this fetus, there is not even a single similarity between defects of muscles attached to the phylogenetically older pectoral *vs*. pelvic girdles.

This leads us to the last key subject to be discussed in this paper: the UL-LL serial homology *vs*. homoplasy. In the above sections we described numerous differences between the ULs *vs*. LLs of not only the T18 fetus but of the normal phenotype, which seem to reveal profound differences between the UL and LL in general. At the same time, the study of the T18 fetus shows clear cases of peculiar (derived) features revealing a strong integration between the forearm/hand structures and the leg/foot structures. How can these two facts be conciliated? Followers of the UL-LL serial homology dogma can always argue that the profound anatomical differences between the UL and LL were acquired during evolution/development. This is because a true (phylogenetic/historical) UL-LL serial homology requires an ancestral duplication, leading to an ancestral similarity between the pectoral and pelvic appendages [50]. However, this also reveals a major problem with the limb serial homology dogma. By simply stating that any difference between these appendages can always be explained by derived changes leading to dissimilarity, defenders of this dogma enter a circular reasoning that cannot be tested, refusing to accept the vast amount of increasing evidence available that actually does contradict this dogma. Such evidence includes the fact that in the oldest fossil fish discovered so far with both pectoral and pelvic appendages, and also in early developmental stages of all tetrapods studied so far, there are actually also profound differences between these appendages, in particular between their proximal regions (i.e. pectoral girdle and associated structures *vs*. pelvic girdle and associated structures; recently reviewed in [50,51]). Therefore, the only way to avoid this circular reasoning is to elaborate more detailed, specific hypotheses that can be tested empirically.

In order to do so, Diogo et al. [50] specifically proposed some precise tests in which, instead of comparing the appendages as a whole, the comparison concerns the similarity between the proximal regions of the UL and LL *vs*. the similarity between the distal regions of the UL and LL. To avoid the circular reasoning that gross anatomy may simply reflect "superficial" changes occurred during gnathostome evolution or occurring at later developmental stages, they included molecular, embryological, and developmental components in their specific prediction. They predicted that distal regions of the tetrapod UL and LL are more similar to each other than are the proximal regions of these limbs in tetrapods (or of the fish pectoral and pelvic appendages), because the pectoral and pelvic appendages are not serial homologues. Instead, they arose at different geological times and were originally markedly different anatomically, and remained markedly different throughout gnathostome evolution, as reflected by the fact that the girdles and associated muscles of each appendage of all taxa (extant and fossil) continue to be strikingly different. The exceptions are thus the more distal and phylogenetically more recent structures of some derived taxa, such as the zeugopod and autopod bones and muscles of tetrapods, which display a more similar gross anatomy in the UL *vs*. LL, particularly in phylogenetically basal tetrapods such as salamanders. According to this hypothesis, the derived similarity between the UL *vs*. LL is therefore due to homoloplasy, and not to ancestral serial homology. Specifically, it is due to the co-option of similar genes for the development of the derived, peculiar autopod/zeugopod skeleton and soft tissues of both the UL and LL of tetrapods, during the fins-limbs transition. In this sense, this hypothesis is similar to the idea of Roth [72], who used the term "genetic piracy" to designate the derived (homoplasic) co-option of similar genes in the development of the UL and LL during the origin of tetrapods.

The first test of Diogo and colleagues’ hypothesis was developed by these authors and completed by Diogo & Molnar [51]. They compared in detail the gross anatomy of representative taxa of all major groups of extant fish and tetrapods, and showed that, regarding soft tissues such as muscles, there is effectively no similarity at all between the proximal regions of the UL *vs*. LL (e.g. in all tetrapods analyzed the number of muscles of shoulder/arm with clear topological correspondents in the pelvis/thigh is 0). In contrast, there is a high gross similarity between the zeugopod/autopod soft tissues of the tetrapod UL *vs*. LL; for example, in salamanders the number of forearm/hand muscles with clear topological correspondents in the leg/foot is 19. The second, embryological, test of the UL-LL homoplasy hypothesis was then undertaken by Diogo & Tanaka [71] and Diogo & Ziermann [73], who studied in detail the development of the UL and LL muscles of both frogs and salamanders. They examined whether the completely different patterns of proximal UL *vs*. proximal LL seen in adults of these taxa is due to changes occurred during development (i.e. serial homology hypothesis) or just reflects differences displayed from the earlier developmental stages (i.e. homoplasy hypothesis). Their results supported the latter scenario because in all developmental stages of both frogs and salamanders the number of shoulder/arm muscles with topological correspondents in the pelvis/thigh is always 0. That is, there are profound differences in the soft tissues, and also in the skeleton, of the proximal regions of the pectoral *vs*. pelvic appendages from the very early stages of development. The third and fourth tests of the UL-LL homoplasy hypothesis were undertaken by Sears et al. [74]. They compared the similarity between the genetic networks and developmental mechanisms involved in the morphogenesis of the shoulder *vs*. pelvis and of the forearm/hand *vs*. the left/foot in model tetrapod organisms. As predicted by the UL-LL homoplasy hypothesis, the genetic networks and developmental mechanisms involved in the formation of the pelvis are markedly different from those involved in the formation of the shoulder, while those involved in the formation of the more derived hand/forearm *vs*. foot/leg regions are much more similar, likely due to derived genetic co-option, as hypothesized by Roth [72] and Diogo et al. [50].

In the present work we thus present the fifth test of the UL-LL homoplasy hypothesis, which concerns anatomical networks. The idea is that, if there is an ancestral similarity between the proximal regions of the UL and LL due to serial homology, that might not be easily recognizable in more superficial/gross anatomical studies due to secondary developmental/evolutionary loss; perhaps the study of the more intricate, fine connectivity network patterns will still be able to recover at least some aspects of this ancestral similarity. Therefore, apart from the various points noted above about the significant qualitative network differences between the UL *vs*. LL, we used AnNA to quantitatively compare the parameters of the intricate network musculoskeletal organization of four datasets for all limbs, including not only the normal adult and newborn phenotypes, but also the abnormal T18 limbs: 1) parameters for each UL (i.e. normal newborn/adult and left/right T18) including only the proximal bones and muscles of these limbs, mainly structures associated/attached to the girdle; 2) parameters for each UL including only the distal bones and muscles of these limbs, mainly zeugopodia/autopodial structures; 3 and 4) same as 1 and 2, but for LLs instead of ULs. The results (*S2 Results*) are as predicted by the homoplasy model: in the overall, for all conditions the network organization of the distal structures of the UL is quantitatively much more similar to that of the distal structures of the LL than is that of the proximal structures of the UL *vs*. the proximal structures of the LL.

Apart from these quantitative AnNA data and the various other AnNA and gross anatomical qualitative comparisons between the human normal UL and LL, there are also further qualitative data supporting the UL-LL homoplasy hypothesis. For instance, regarding the T18 fetus studied for the present work, the qualitative comparison between the T18 ULs *vs*. LLs supports and complements the quantitative data, indicating that there is significantly higher similarity between the network organization of the distal structures of the ULs *vs*. LLs than there is between more proximal structures of these limbs (i.e. related/close to the girdles). There are various similar changes in the ULs and LLs of this T18 fetus, such as the loss of a tendon to digit 5 of the hand/foot by at least some forearm/leg muscles of these limbs. However, there is *not even a single* abnormal transformation in any proximal structure of the ULs that has even any general resemblance to any change in the LLs of this fetus, and vice-versa. For instance, in the T18 left UL there is an abnormal (supernumerary) presence of a bundle (coracobrachialis profundus) of the proximal coracobrachialis muscle, and in both left and right T18 ULs there is an extra biceps brachii tendon inserting onto the pectoralis major [49]. In the T18 LLs, there is not any supernumerary proximal muscle bundle or tendon. Also, in both T18 ULs, the rhomboideus major and minor are deeply blended, as are the pectoralis major and deltoideus, and on the right T18 UL there is a fusion between the coracobrachialis and the biceps brachii. However, there is no blending or fusion between any of the proximal muscles of the LLs. Furthermore, on the left T18 UL, there is no short head of the biceps brachii, while there is no head or bundle or tendon of any proximal muscle missing in the T18 LLs.

Why should the distal regions of the LLs *vs*. ULs of the T18 fetus display similar abnormalities, while the proximal regions of these limbs do not display even a single similar defect, if there was a true serial homology between the LLs and ULs? To our knowledge, there is no way of answering these broader comparative and pathological questions using the UL-LL serial homology dogma, and that shows its major flaw: by being so vague, and often defended by circular reasoning, it becomes almost impossible to contradict because one can always argue that ancestral similarities were simply lost; this dogma ends up by explaining almost nothing at all and lacking specific predictable power. In contrast, the homoplasy LL-UL hypothesis states that there was an ancestral dissimilarity between the pectoral and pelvic appendages followed by a derived (homoplasic) gene co-option of similar genes for the development of newly acquired distal structures of the UL and LL, such as the forearm/leg and hand/foot, which is testable and has a very specific predictable power. For instance, it is able to explain the results of all five tests mentioned above, as well as the other evolutionary, developmental, and network data mentioned in this paper, including those concerning birth defects. If, for example, any of the five tests have failed (by showing a higher similarity between the proximal regions of the UL *vs*. LL than between distal regions, for any of the five types of data), this UL-LL hypothesis would be contradicted. However, within all cases studied and data obtained so far, either quantitative or qualitative, or from normal or abnormal development, this hypothesis continues to stand, thus becoming stronger.

### Future research directions

There are various ways of expanding the use of AnNA to study the development, evolution and pathology of limbs. Concerning evolution, it is important to expand musculoskeletal AnNA to other primates, including key human fossils through detailed muscle reconstructions, as well as other tetrapods. For instance, this will enable to investigate if there are cases where network UL-LL differences seen in the normal human phenotype might be the result of changes from cases of ancestral similarity, or if some similarities found in humans are derived and not seen in other tetrapods. Such studies will also enable a better understating of which features are unique for humans, and thus pave the way for further studies about human evolution in a broader functional and ecological context. With respect to development, as noted above, we plan to include more developmental stages for both normal and abnormal development, and also add other pathology cases. In particular, concerning future directions with medical implications, AnNA can be employed to better understand changes in modularity and integration when bones or whole digits are lost or duplicated in the UL or LL, because non-pentadactyly is one of the most common birth defects in humans. Lastly, regarding developing further types of analyses within the AnNA methodology itself, it would be interesting to connect AnNA with theoretical network discussions on small-world organization, for instance, identifying which elements of a certain network bear an important role in holding together otherwise separate parts of that anatomical network [75]. These elements are often referred as "weak ties" in the literature, because their failure to establish connections (e.g. due to loss of the element) disturbs severely the organization of the network. Weak ties can be identified using network parameters such as clustering coefficient (low values) and betweenness centrality (high values). For example, the "weak tie" in the normal adult UL skeletal system is the humerus. Our exploratory analyses of the network structure shows that when the humerus is removed from the limb network some elements become disconnected. However, the whole UL musculoskeletal network does not split into two or more groups of elements and thus the overall organization is not disturbed to the degree of collapsing (e.g. forearm structures do not become completely disconnect from more proximal structures such as the girdle). We plan to further develop these analyses in the future, as well as to pave the way for, and stimulate, future research by other researchers, using AnNA.

## Methods

For this work, we studied the musculoskeletal structures of the ULs and LLs of a 28-week old T18 cyclopic fetus and in karyotypically normal newborn and adult humans. Details about the dissections of the T18 fetus were recently provided in detail by Reid et al. [76], Gondré-Lewis et al. [77], and Smith et al. [49]. The descriptions and network matrices done for the normal newborn and adult configuration are based those dissections of human newborns and our own review of the literature, which allowed us to carefully establish the normal (i.e. most commonly found) phenotype seen in our species [28,33,49,51]. No dissections of human specimens have been carried out for the present study. Anatomical networks of the normal adult human head were described by Esteve-Altava et al. [29], and were recently briefly compared to the ULs [46]. Here we provide the first detailed analyses specifically focused on the limbs themselves, and particularly on the comparisons between upper and lower, newborn and adult, and normal and abnormal limbs.

### Anatomical network modeling

We built network models for the ULs and LLs of the T18 fetus and the normal newborn and adult phenotypes. We have built left and right network models for the T18 fetus limbs because they are asymmetric in the number of bones/muscles and the connections among them. Physical articulations among anatomical parts (bones and/or muscles) were modeled as a system of connections among nodes. This information is coded in an adjacency matrix: a square symmetric matrix where each row and column represents one anatomical part, and the presence/absence of contacts between two anatomical parts is formalized with a 1/0 notation. These adjacency matrices are openly available from http://dx.doi.org/10.6084/m9.figshare.1431463.

We analyzed and compared the skeletal, the muscular, and the musculoskeletal components of each limb. For each component, we built a network model using different definitions of node and connection. Thus, in skeletal networks, nodes represent the bones and cartilages and connections represent the physical articulations among them (i.e. sutures, synchondroses and synovial joints). In muscular networks, nodes represent muscles and connections represent tendinous joints and fibrous fusions among them. Finally, in musculoskeletal networks, nodes represent both bones and muscles, whereas connections represent all the above-described physical junctions among them.

### Network analysis

We have analyzed the general architecture and the community structure of each of these anatomical network models (for a total of 24). The sections below summarize the analyses that were carried out. Further details of the use of network analysis in anatomical systems are given in previous works [29,44]. Details of the results retrieved are given in the Supplementary Information. These include the network parameters (*S1 Results*), the number and composition of the modules identified, the dendrograms showing the hierarchical grouping, and network plots featuring modularity. We performed these analyses in R using functions from the *igraph* package [78]. See *S2 Methods* for a detailed protocol and *S3 Methods* for the data used in the analysis.

### Quantifying basic network parameters

For each anatomical system, we quantified the following parameters: density of connections (D), average clustering coefficient (C), average shortest path length (L), the longest of all shortest paths (Diameter), and heterogeneity of connections (H). Specifically, D is the number of existing connections with respect to the total maximum possible according to the total number of nodes, D = 2K/N(N − 1), where *K* is the number of connections in the network and *N* is the number of nodes; C is the arithmetic mean of the clustering coefficient of all nodes in the network, $C=\frac{1}{N}\sum \left(\sum \tau_{i}/\sum k_{i}(k_{i}-1)\right)$, where *τ*_*i*_ is the number of triangular motifs including node *i*, and *k*_*i*_ is the number of connections of node *i*; L is the arithmetic mean of the shortest path length between all pairs of nodes in the network, $L=\frac{1}{N-1}\sum l_{i,j}$, where *l*_*i*,*j*_ is the minimum number of connections that connects nodes *i* and *j*; the Diameter is the longest of all shortest paths; and, finally, H is the ratio between the standard deviation and the mean of connectivity, *H* = *σ*_*K*_/*μ*_*K*_, where *σ*_*K*_ is the standard deviation of the number of connections of all nodes in the network and *μ*_*K*_ is the mean of the number of connections.

### Estimating the small-world and hierarchical organization

We assessed the presence of the small-world phenomena in each anatomical system by comparing the values of C and L of the corresponding network model with those of 10,000 random equivalent networks (i.e. the same number of nodes, but re-connected at random) [79]. After correcting for the size, an empirical network is small-world if it fulfills the following condition [80]: $\frac{C/{\overset{-}{C}}_{rand}}{L/{\overset{-}{L}}_{rand}}\geq 0.012\times N^{1.11}$. A small-world network has an organization between regularity and randomness, which facilitates the formation of meaningful modules. This means that connectivity modules in small-world anatomical systems are not due to chance, and hence, they are expected to be morphologically meaningful.

We estimated the presence of a hierarchical organization of connections in each anatomical system by testing the fit of its network model’s connectivity distribution, P(*k*), and clustering coefficient distribution, C(*k*), to a power-law distribution function [81]. The P(*k*) is the probability to find a node with a given number of connections in the network: P(*k*) = n_k_/N. The C(*k*) is the mean clustering coefficient of all nodes with *k* connections: C(*k*) = c_i,k_/N. Theoretically, the P(*k*) and the C(*k*) in a hierarchical network tend to fit a power-law distribution (e.g. P(*k*) = c × *k* ^−α^). This type of organization emerges as a consequence of highly clustered groups of nodes, which is directly related with the formation of modules.

### Identifying the modular organization

A connectivity module is here defined as a group of anatomical parts (bones and muscles) with more connections among them than to others outside their group. To identify the connectivity modules that make each anatomical system we used a 3-step walk-trap algorithm [82]. This algorithm outputs a nested aggrupation of nodes (i.e. as a dendrogram; *S4 Methods*). To decide among all potential partitions, we quantified the modularity Q-value of each one. Q is the difference between the actual proportion of connections among nodes in the same module and the proportion expected in a random network, $Q=\frac{1}{2K}\sum_{i,j}\left[A_{ij}-\frac{k_{i}\times k_{j}}{2k}\right]\times \delta (m_{i},m_{j})$, where *K* is the number of connections, *A*_*ij*_ is the adjacency matrix, *k*_*i*_ is the connections of *i*, *k*_*j*_ that of *j*, *m*_*i*_ is the module of *i*, and *m*_*j*_ that of *j*. If *m*_*i*_ = *m*_*j*_ then *δ(m*_*i*_,*m*_*j*_ *)* = 1, else *δ(m*_*i*_,*m*_*j*_ *)* = 0. Q ranges from -1 to 1: if the number of connections among nodes in the same module is not higher than expected at random, then Q ≤ 0, otherwise Q > 0. In networks with a strong modularity, Q varies from 0.3 to 0.7 (higher values being rare). The higher the Q, the better the partition.

### Quantitative comparison of morphological similarity

We have compared how similar are the modular organization of the ULs and LLs (i.e. number of modules and constitutive parts) of the T18 fetus and the normal newborn, by quantifying the ratio of anatomical elements (bones and muscles) grouped in a same module in both networks at the same time divided by the total number of elements in common. The labels of bones are used to check what elements are common to both networks. The function used to make this quantification is given in *S3 Methods*.

### Quantitative comparison of functional and developmental hypotheses

We have compared the network partition in modules of each limb with functional and developmental null hypotheses. We elaborated detailed tables dividing the structures seen in the normal human ULs and LLs in functional and developmental groups (*S5 Methods*; *S6 Methods*), based on previous comparative, evolutionary and developmental work and our review of the literature [28,50,51,66,70,83,84]. By doing this, we can directly compare, for each system, the overall modular organization of the network with those functional *vs*. developmental groupings, and therefore examine which of these groupings is more similar to the network organization reported. This more objective and informed quantitative methodology avoids the risk of circular reasoning, such as that often done in studies of integration and modularity (see comments by Ross [39]). We estimated the assessed the match between network modules and null hypotheses of modularity using a normalized mutual information index, based on information theory [85]: index is 0 when the two divisions match completely, and it is 1 when they are totally different. The normalized mutual information index was quantified using function compare in the *igraph* package in R.

## Supporting Information

S1 Fig Newborn upper limb skeletal network. See S1 Methods for labels of nodes and legend of Figs 1 to 8 for color/module correspondence.
(PDF)

S2 Fig Adult upper limb skeletal network. Legend idem than S1 Fig.
(PDF)

S3 Fig Newborn upper limb muscular network. Legend idem than S1 Fig.
(PDF)

S4 Fig Adult upper limb muscular network. Legend idem than S1 Fig.
(PDF)

S5 Fig Newborn upper limb musculoskeletal network. Legend idem than S1 Fig.
(PDF)

S6 Fig Adult upper limb musculoskeletal network. Legend idem than S1 Fig.
(PDF)

S7 Fig Newborn lower limb skeletal network. Legend idem than S1 Fig.
(PDF)

S8 Fig Adult lower limb skeletal network. Legend idem than S1 Fig.
(PDF)

S9 Fig Newborn lower limb muscular network. Legend idem than S1 Fig.
(PDF)

S10 Fig Adult lower limb muscular network. Legend idem than S1 Fig.
(PDF)

S11 Fig Newborn lower limb musculoskeletal network. Legend idem than S1 Fig.
(PDF)

S12 Fig Adult lower limb musculoskeletal network. Legend idem than S1 Fig.
(PDF)

S13 Fig T18 left upper limb skeletal network. Legend idem than S1 Fig.
(PDF)

S14 Fig T18 left upper limb muscular network. Legend idem than S1 Fig.
(PDF)

S15 Fig T18 left upper limb musculoskeletal network. Legend idem than S1 Fig.
(PDF)

S16 Fig T18 right upper limb skeletal network. Legend idem than S1 Fig.
(PDF)

S17 Fig T18 right upper limb muscular network. Legend idem than S1 Fig.
(PDF)

S18 Fig T18 right upper limb musculoskeletal network. Legend idem than S1 Fig.
(PDF)

S19 Fig T18 left lower limb skeletal network. Legend idem than S1 Fig.
(PDF)

S20 Fig T18 left lower limb muscular network. Legend idem than S1 Fig.
(PDF)

S21 Fig T18 left lower limb musculoskeletal network. Legend idem than S1 Fig.
(PDF)

S22 Fig T18 right lower limb skeletal network. Legend idem than S1 Fig.
(PDF)

S23 Fig T18 right lower limb muscular network. Legend idem than S1 Fig.
(PDF)

S24 Fig T18 right lower limb musculoskeletal network. Legend idem than S1 Fig.
(PDF)

S1 Methods Labels of nodes in network plots. (XLS)

S2 Methods Protocol of AnNA of limbs. (RMD)

S3 Methods Data generated in the AnNA of limbs. (RDATA)

S4 Methods Dendrograms generated by the walk-trap community detection algorithm. (PDF)

S5 Methods Description of developmental and functional hypotheses used in the comparison. (DOCX)

S6 Methods Code of the developmental and functional hypotheses. (XLS)

S1 Results Network parameters for each anatomical system. (DOCX)

S2 Results Network parameters for proximal and distal sub-networks. (XLS)

S3 Results Similarity in the modular organization between networks. (XLS)

S4 Results Comparison of network modules with developmental and functional hypotheses. (XLS)
