# Supplementary figures and images for: Anatomical Network Comparison of Human Upper and Lower, Newborn and Adult, and Normal and Abnormal Limbs, with Notes on Development, Pathology and Limb Serial Homology vs. Homoplasy
Source: PLoS One. 2015 Oct 9;10(10):e0140030. doi: 10.1371/journal.pone.0140030 (PMC4599883; doi:10.1371/journal.pone.0140030)

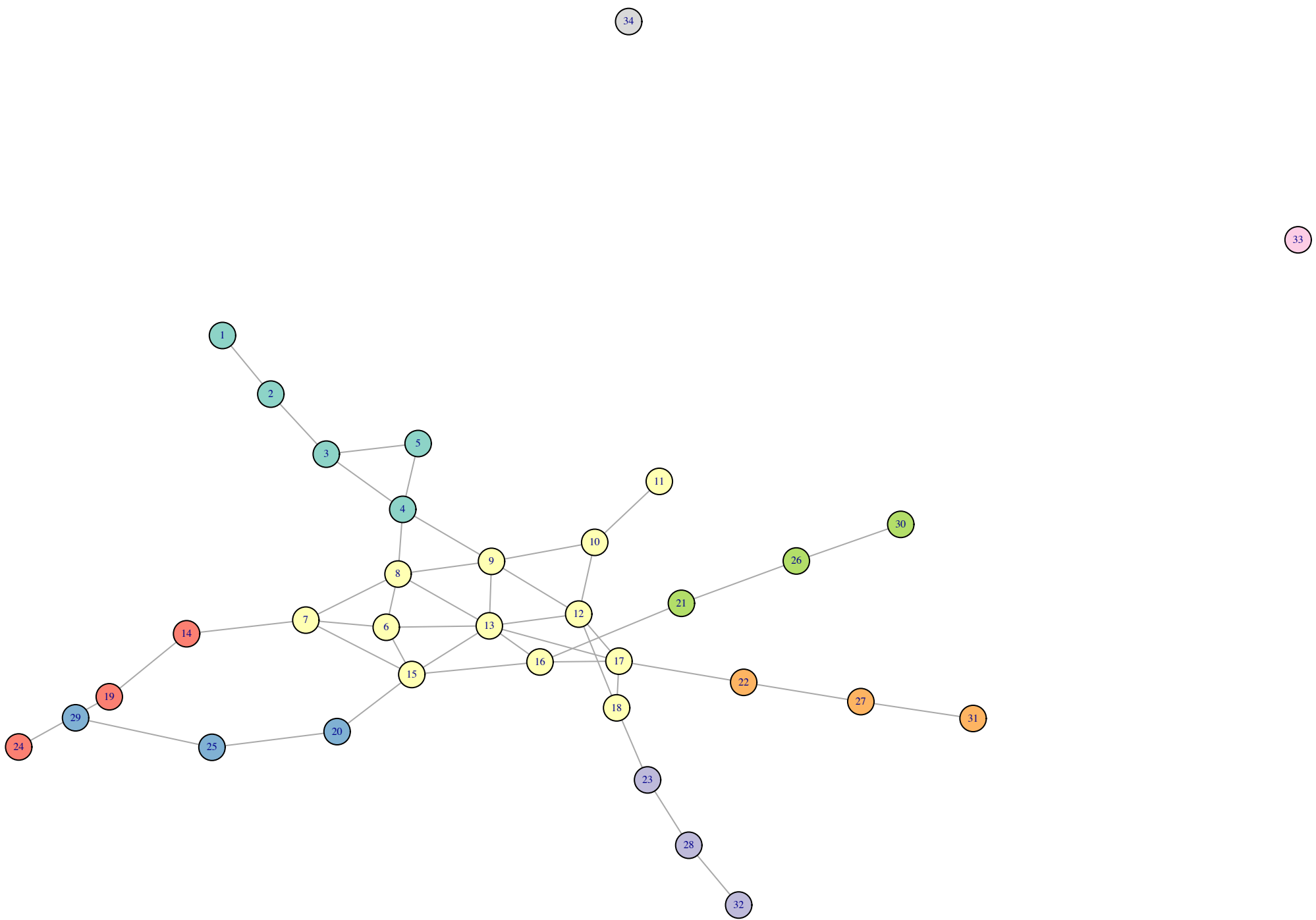

Supplement: S1 Fig — See S1 Methods for labels of nodes and legend of Figs 1 to 8 for color/module correspondence. (PDF) [file pone.0140030.s001.pdf]

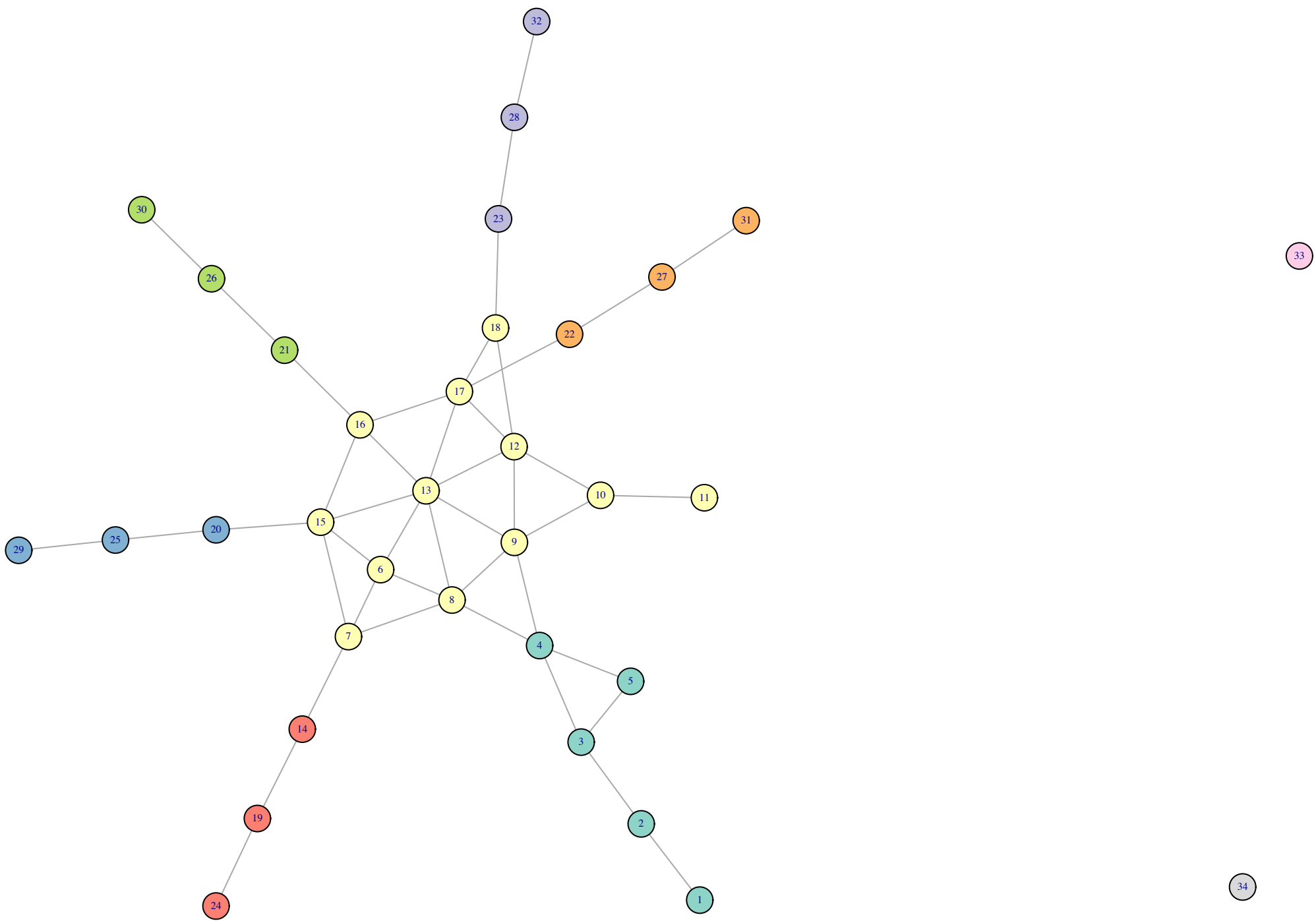

Supplement: S2 Fig — Legend idem than S1 Fig. (PDF) [file pone.0140030.s002.pdf]

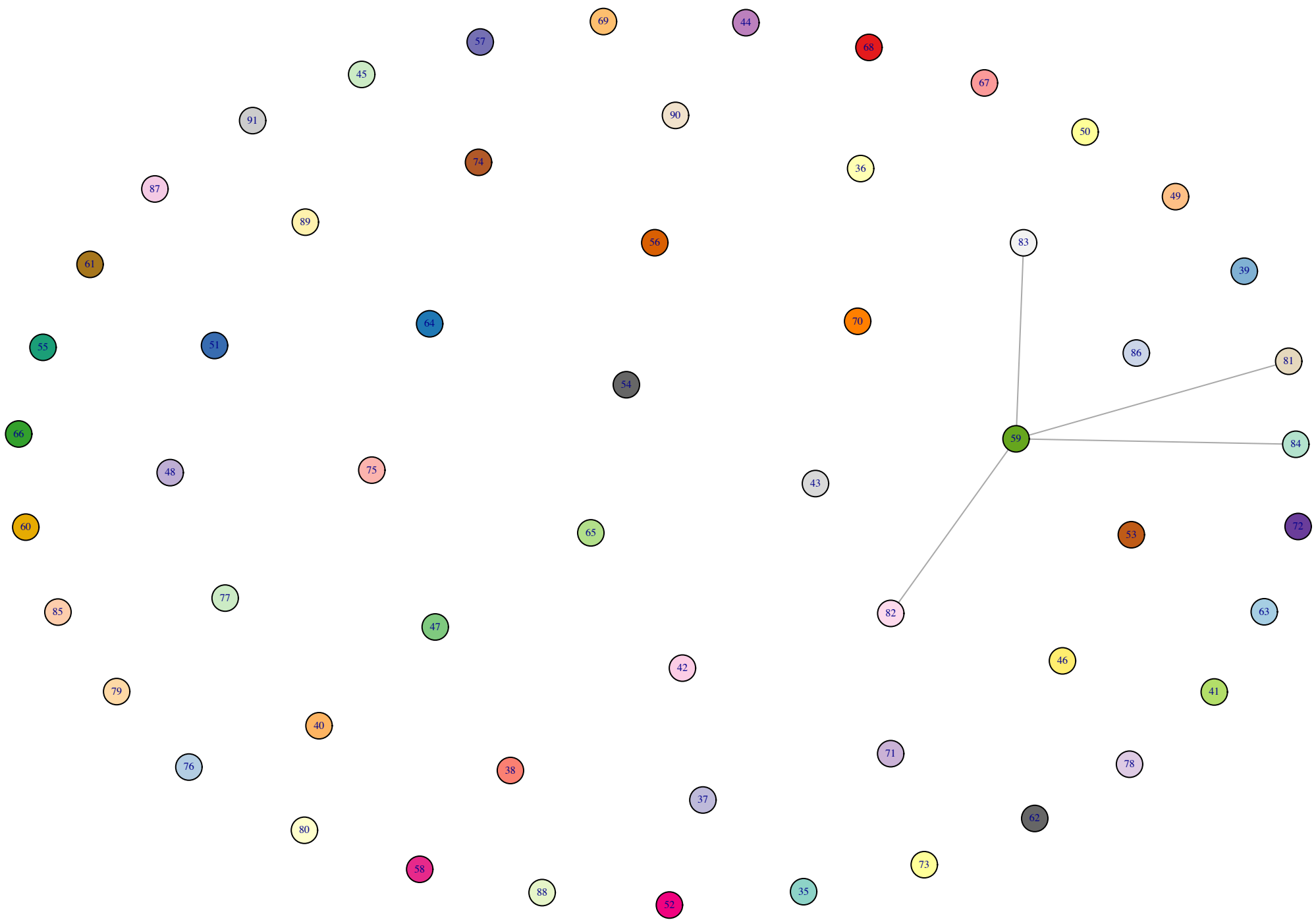

Supplement: S3 Fig — Legend idem than S1 Fig. (PDF) [file pone.0140030.s003.pdf]

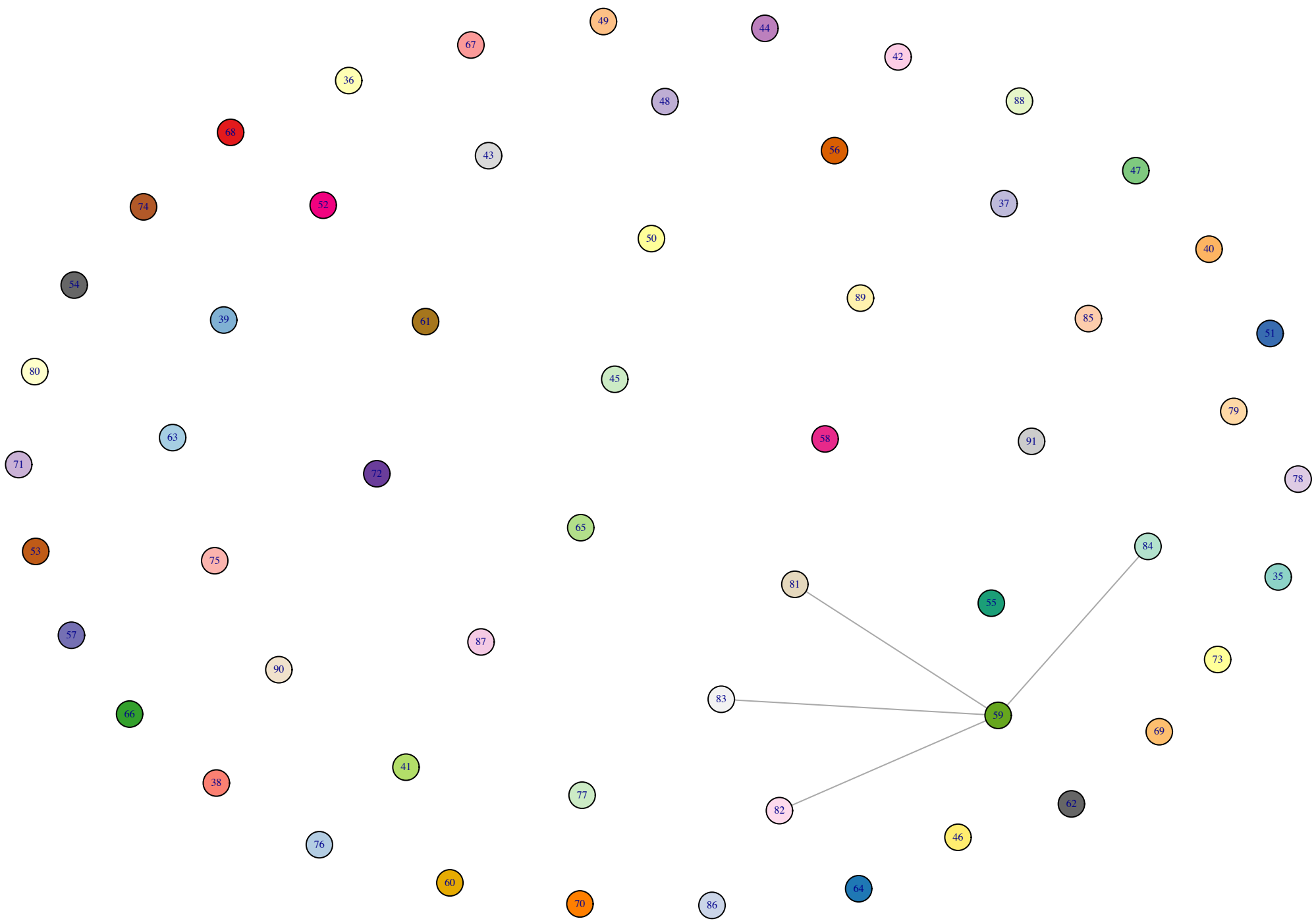

Supplement: S4 Fig — Legend idem than S1 Fig. (PDF) [file pone.0140030.s004.pdf]

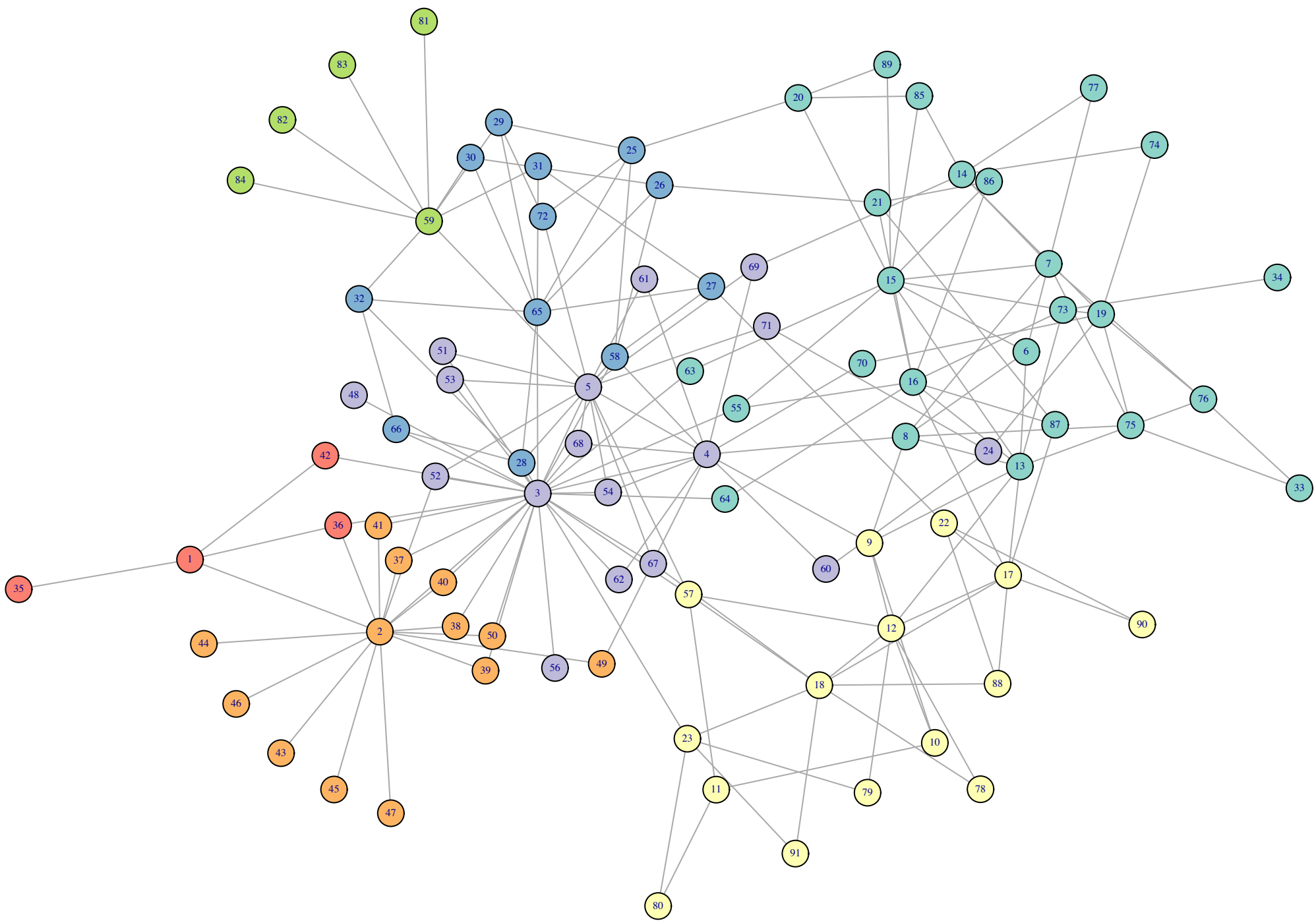

Supplement: S5 Fig — Legend idem than S1 Fig. (PDF) [file pone.0140030.s005.pdf]

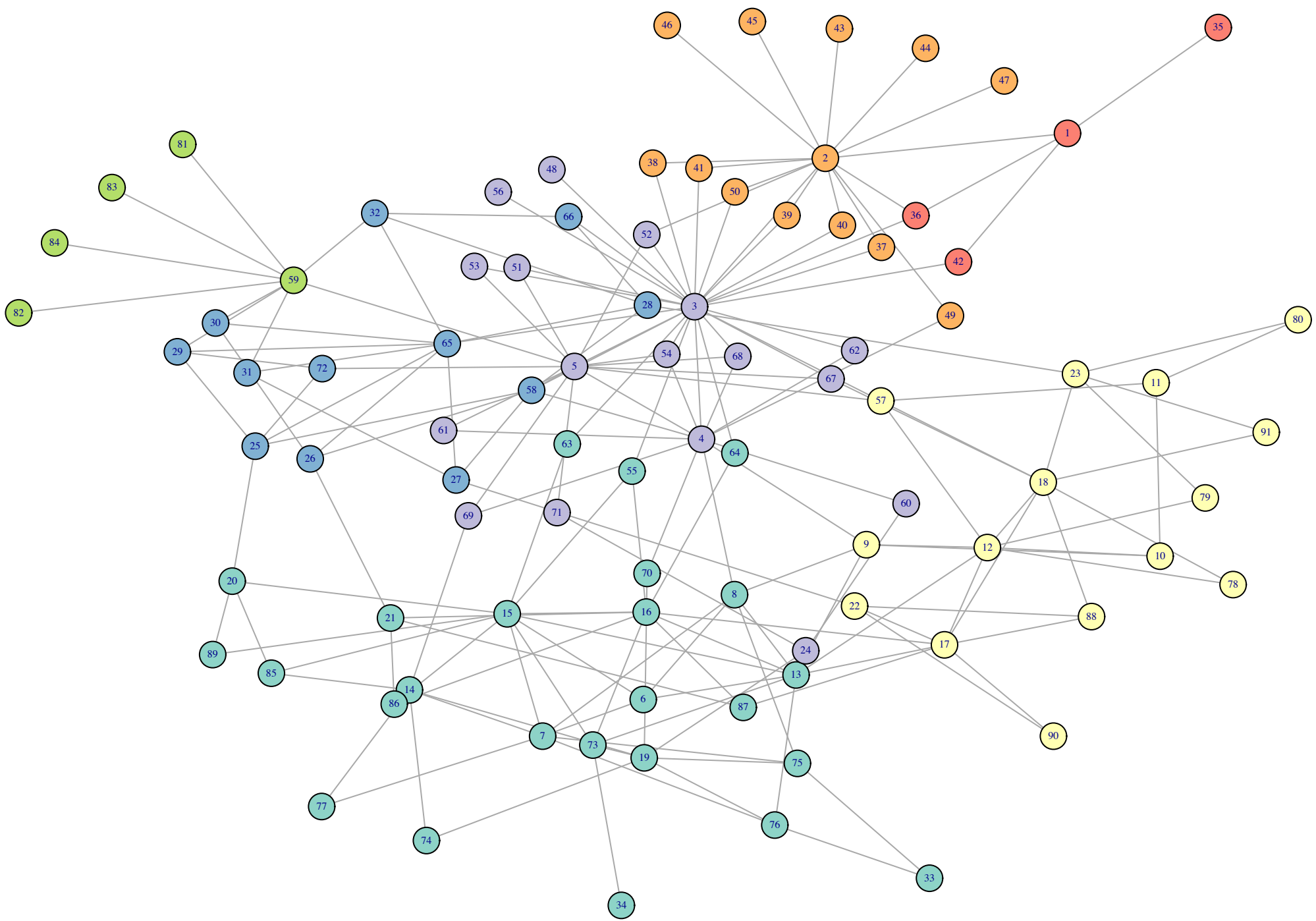

Supplement: S6 Fig — Legend idem than S1 Fig. (PDF) [file pone.0140030.s006.pdf]

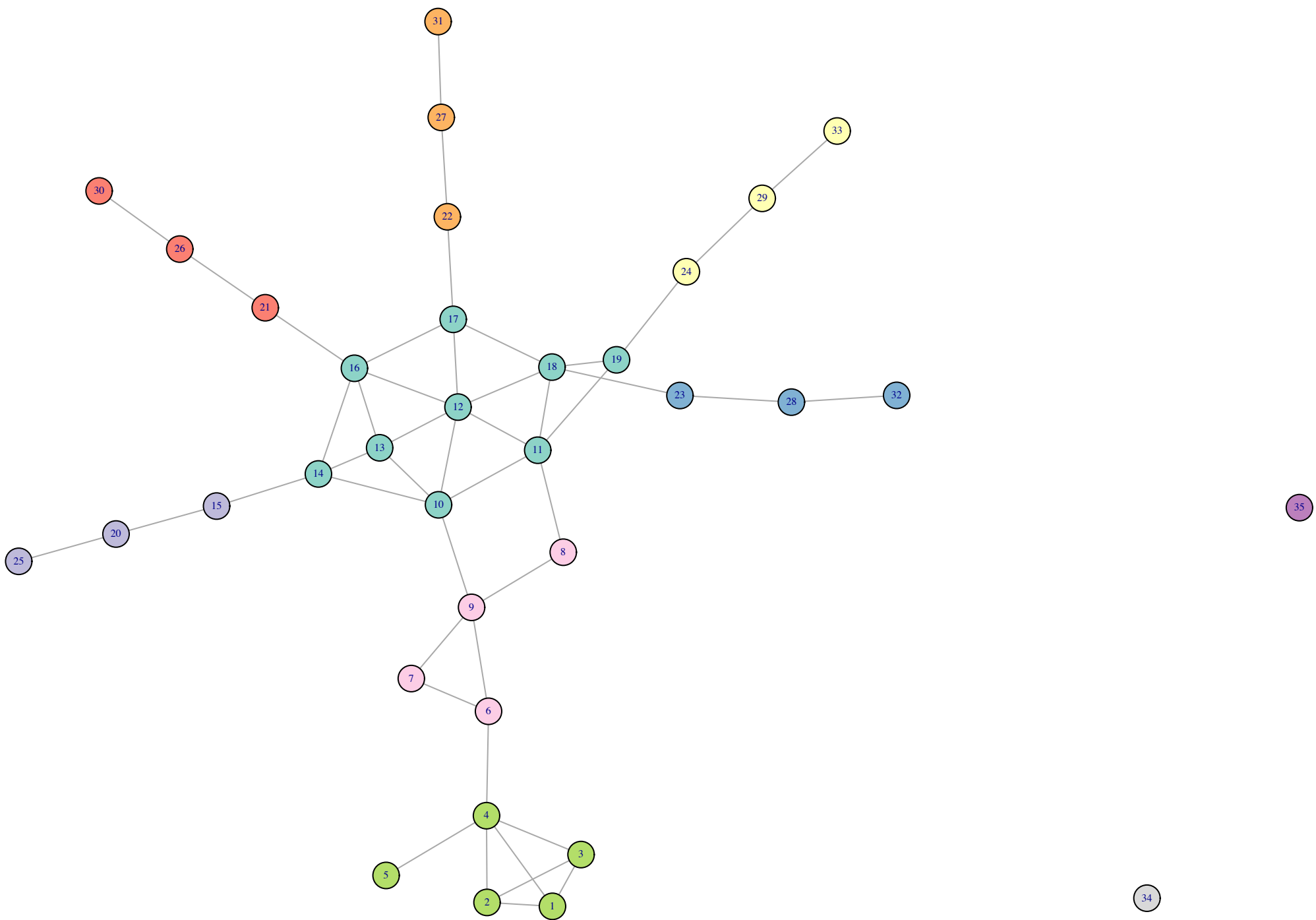

Supplement: S7 Fig — Legend idem than S1 Fig. (PDF) [file pone.0140030.s007.pdf]

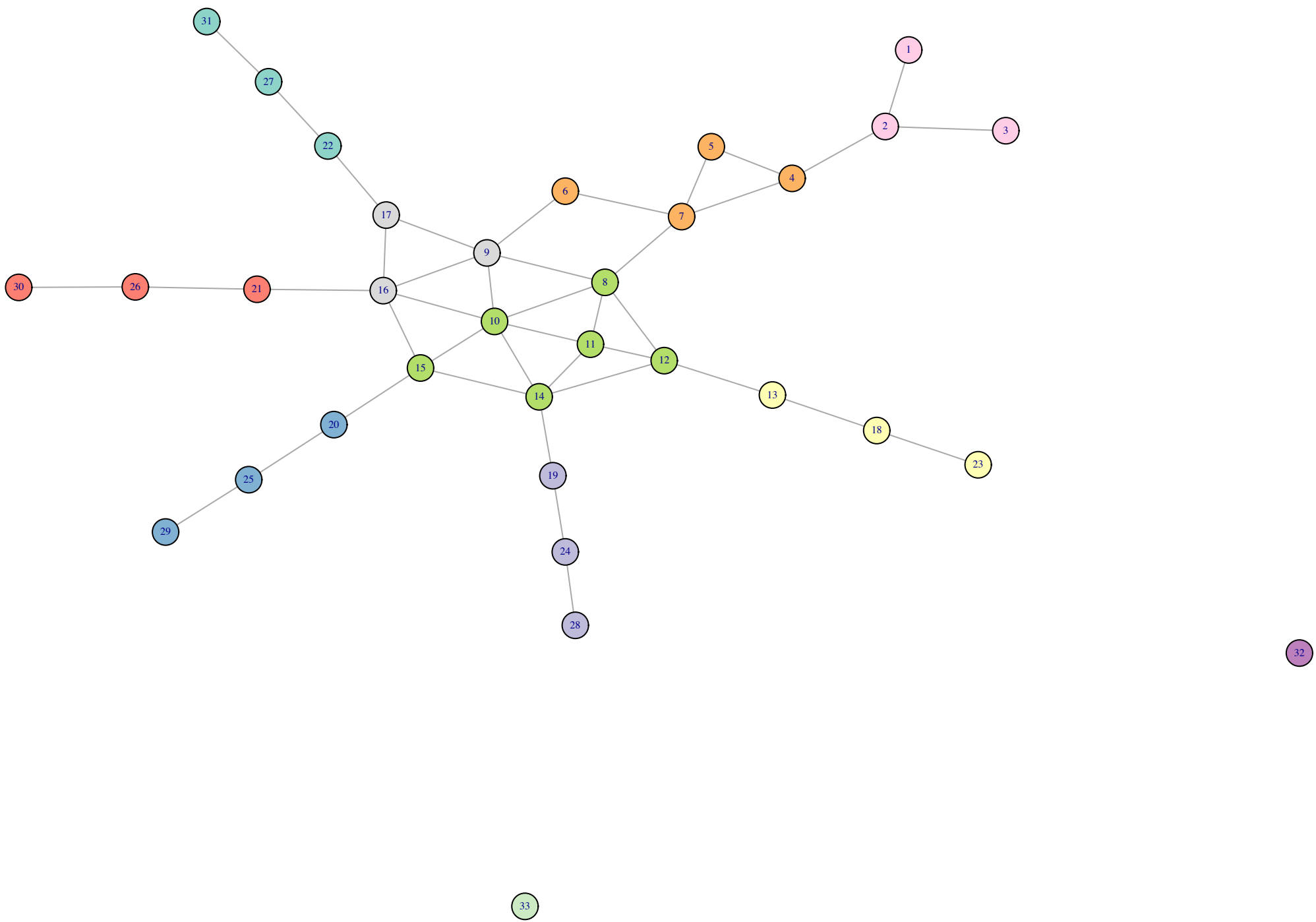

Supplement: S8 Fig — Legend idem than S1 Fig. (PDF) [file pone.0140030.s008.pdf]

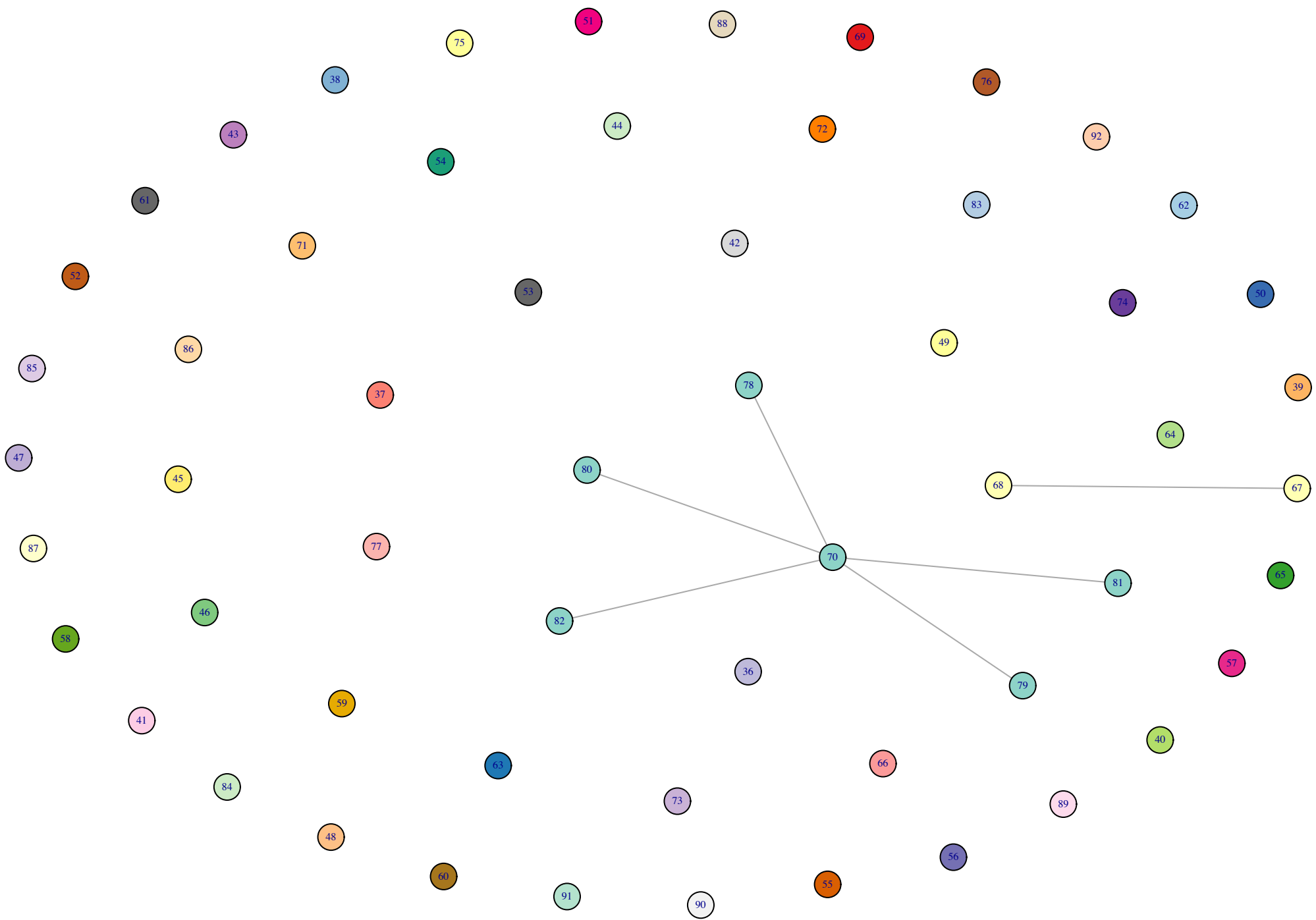

Supplement: S9 Fig — Legend idem than S1 Fig. (PDF) [file pone.0140030.s009.pdf]

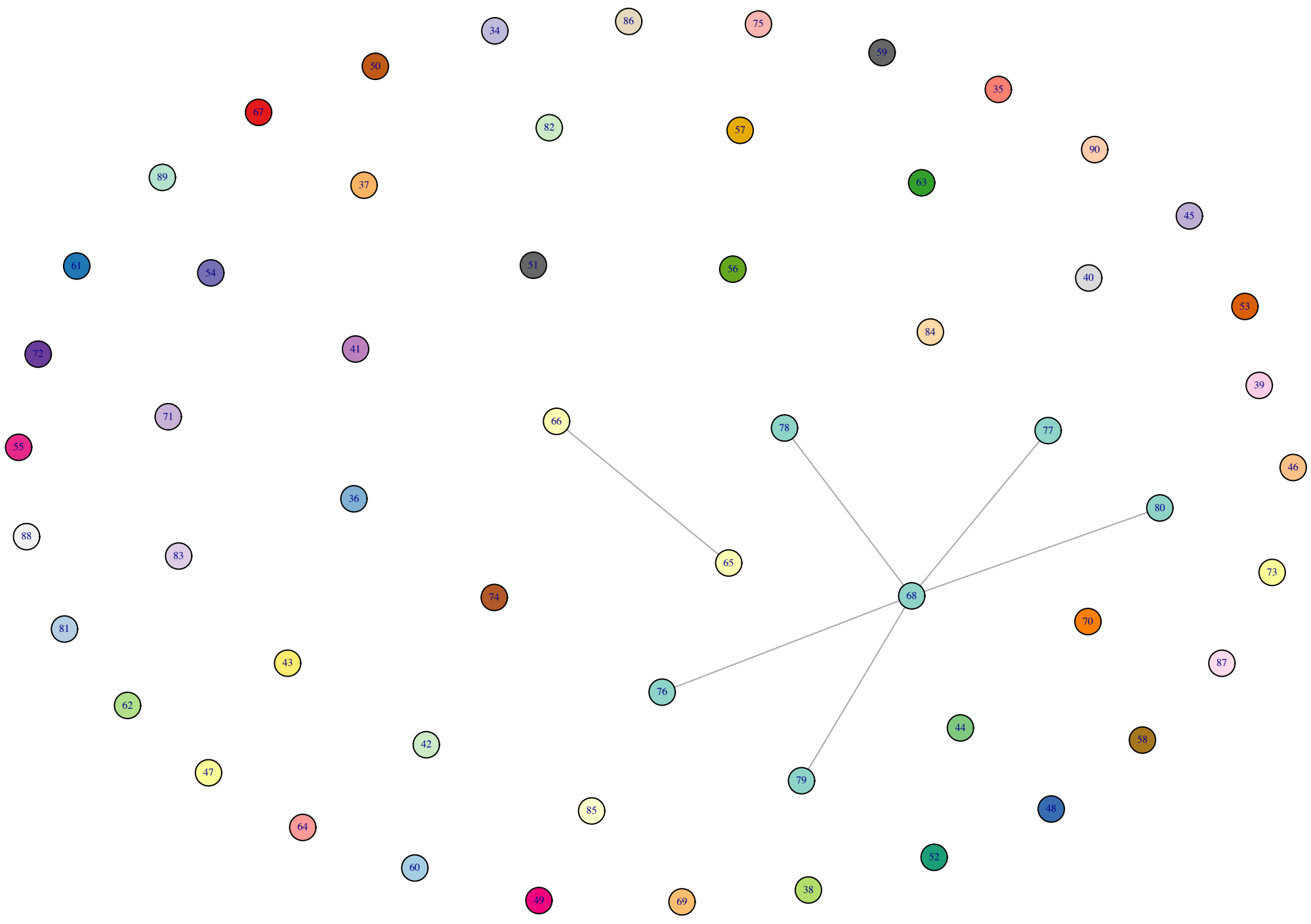

Supplement: S10 Fig — Legend idem than S1 Fig. (PDF) [file pone.0140030.s010.pdf]

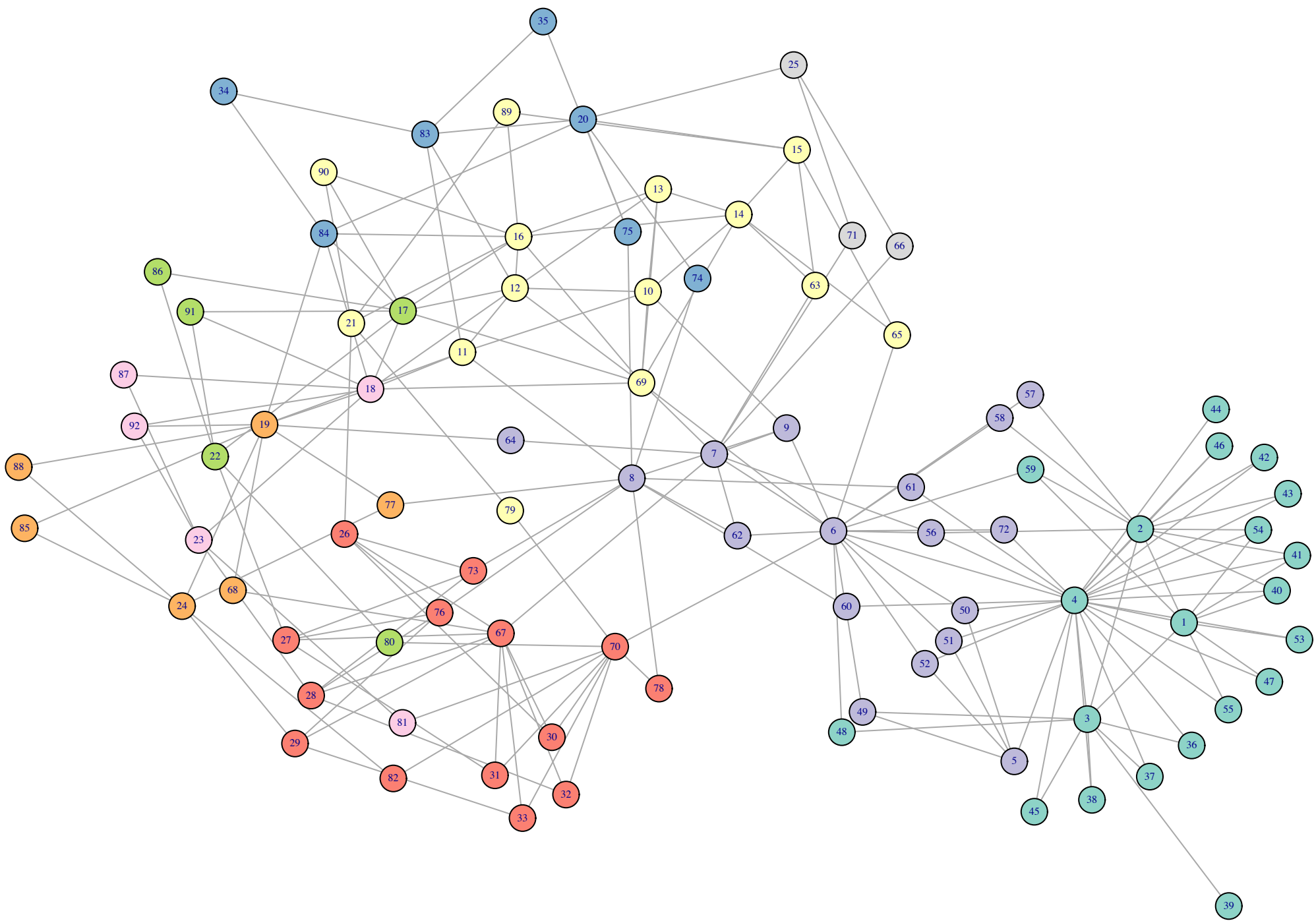

Supplement: S11 Fig — Legend idem than S1 Fig. (PDF) [file pone.0140030.s011.pdf]

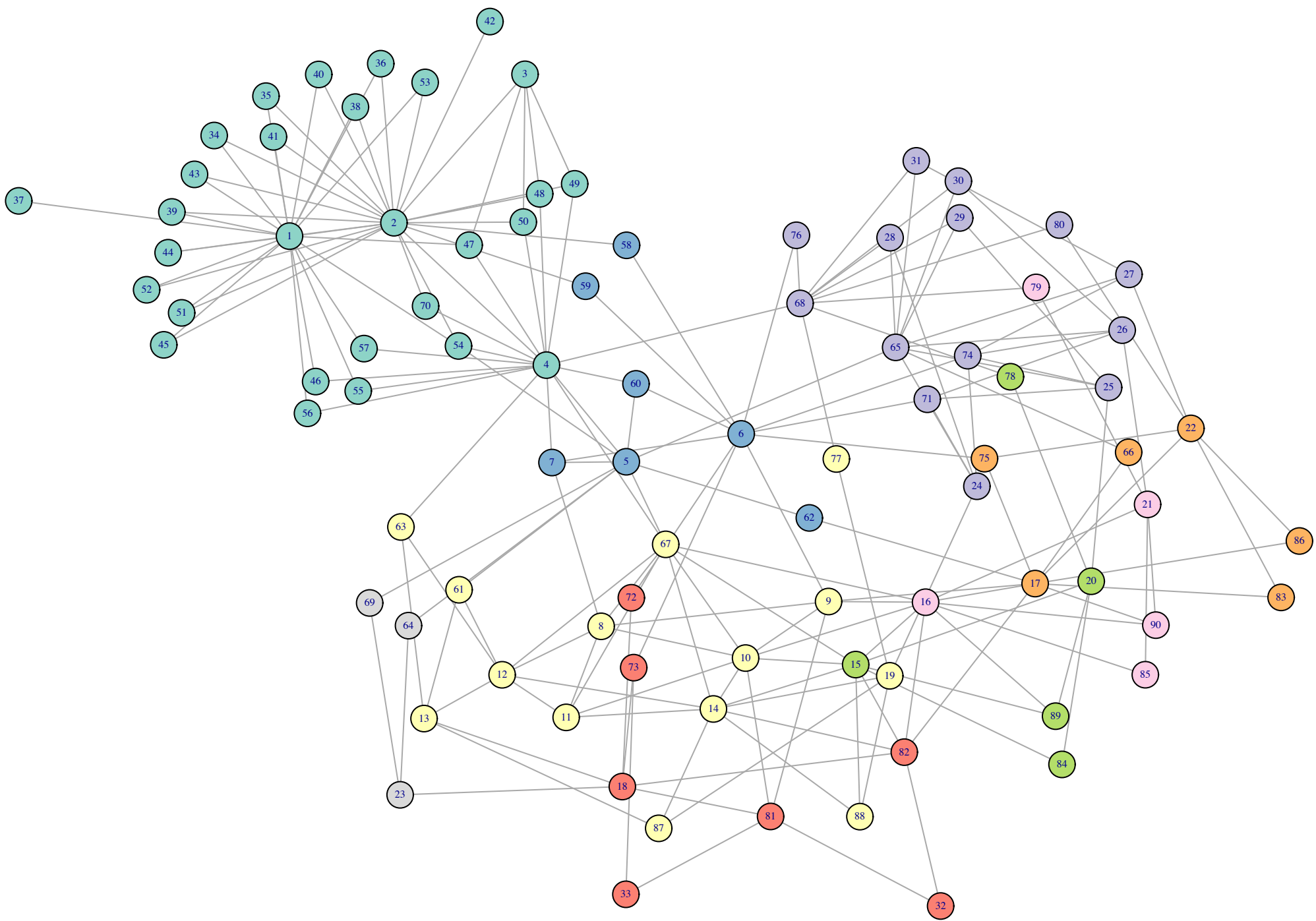

Supplement: S12 Fig — Legend idem than S1 Fig. (PDF) [file pone.0140030.s012.pdf]

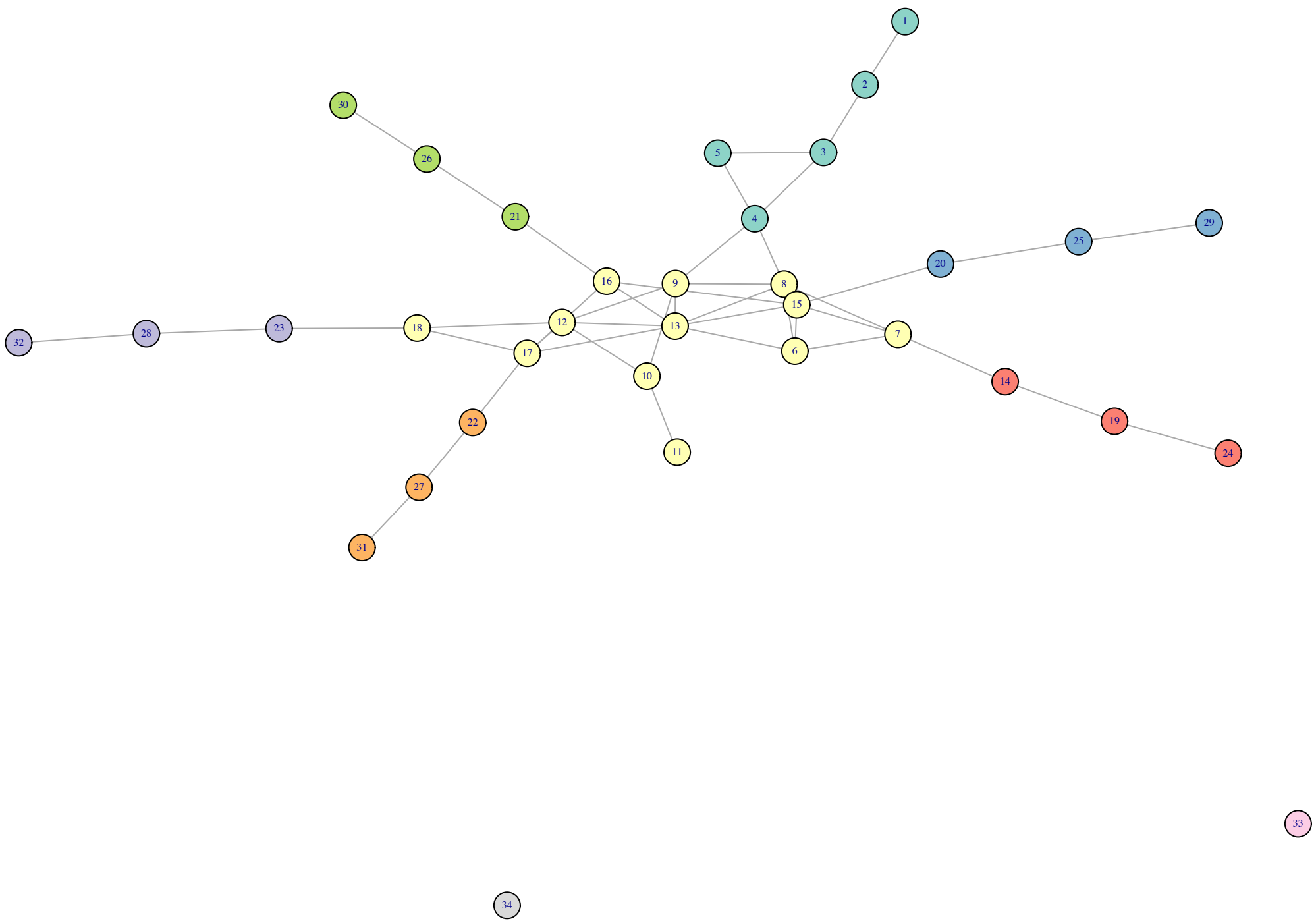

Supplement: S13 Fig — Legend idem than S1 Fig. (PDF) [file pone.0140030.s013.pdf]

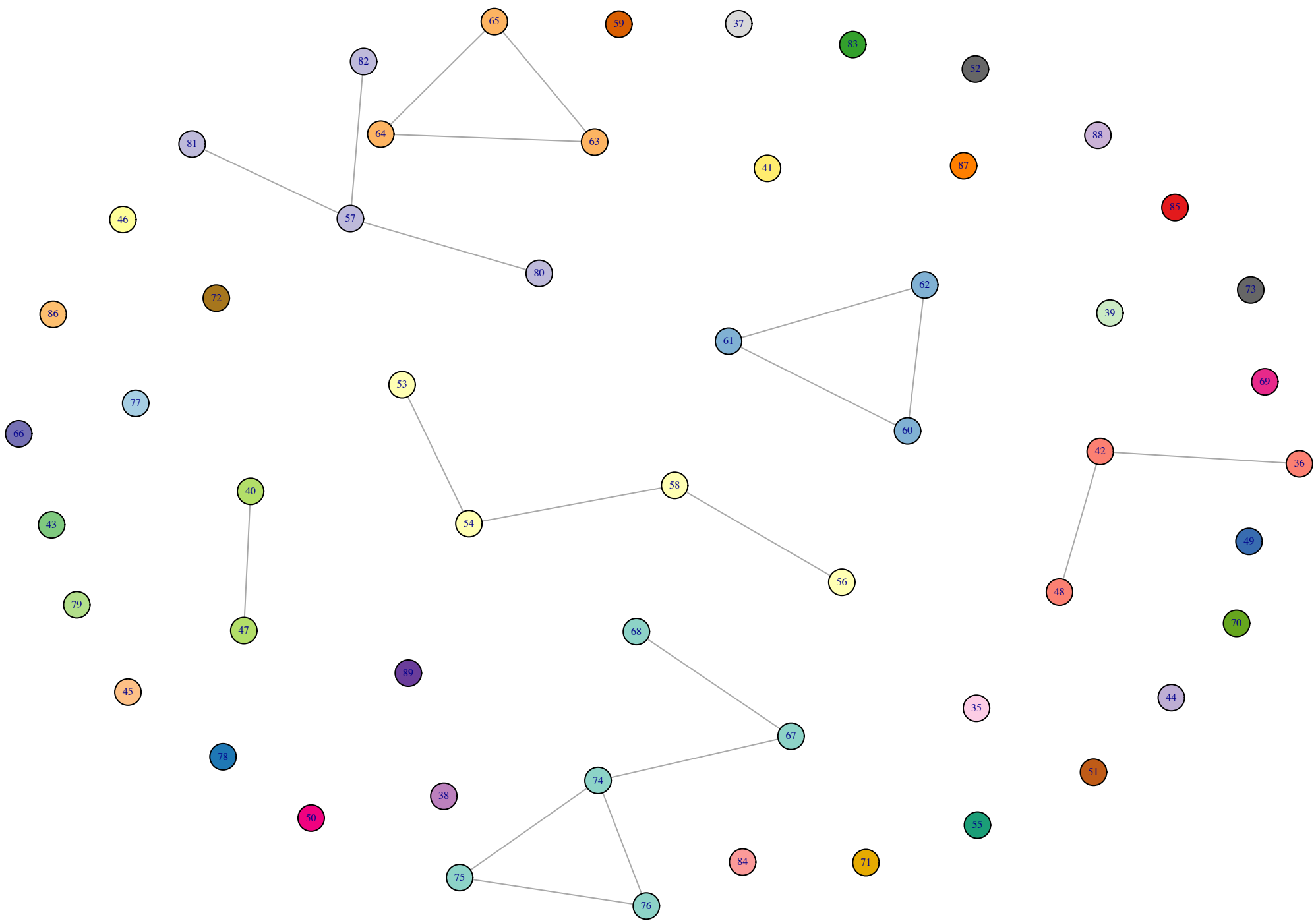

Supplement: S14 Fig — Legend idem than S1 Fig. (PDF) [file pone.0140030.s014.pdf]

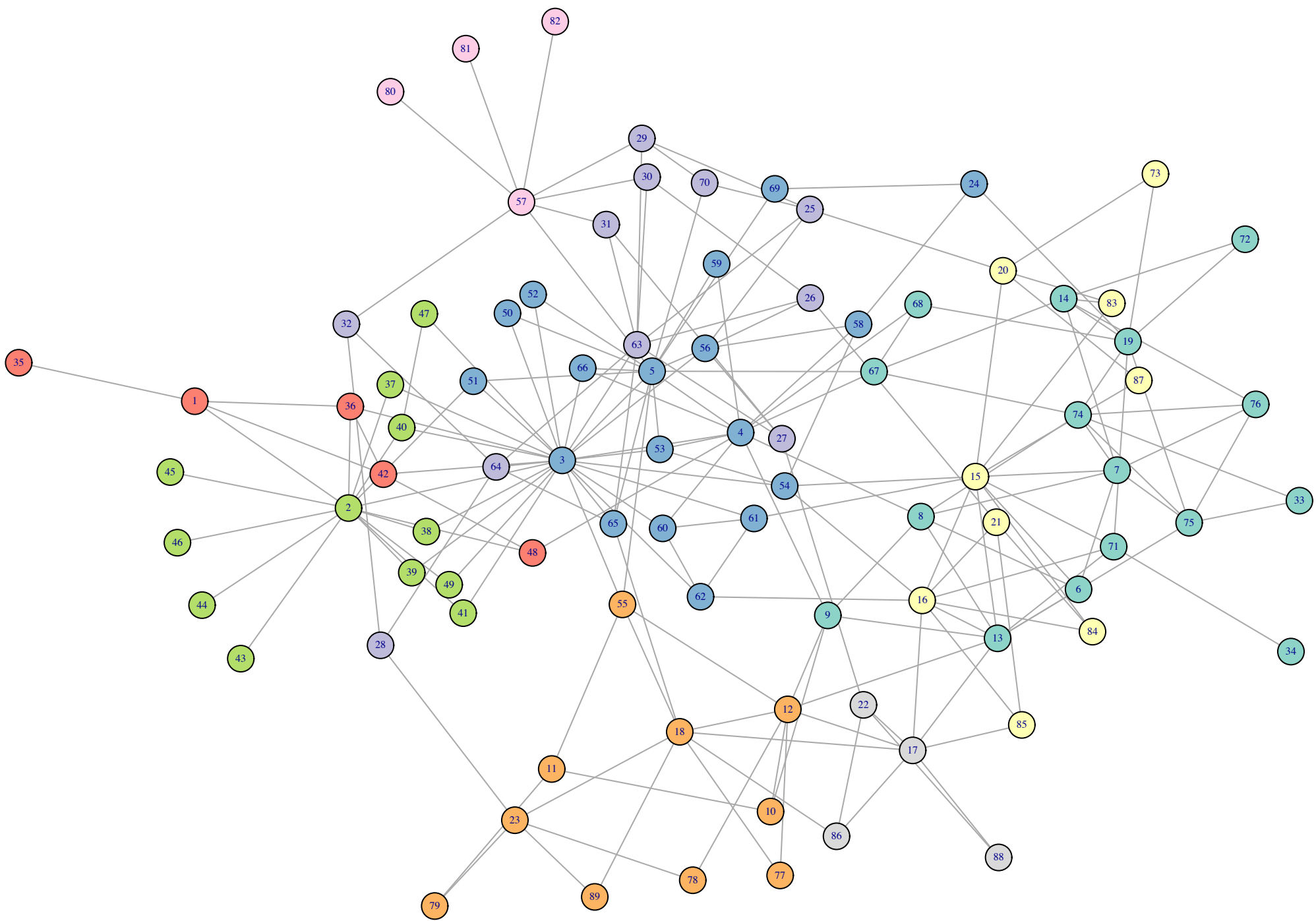

Supplement: S15 Fig — Legend idem than S1 Fig. (PDF) [file pone.0140030.s015.pdf]

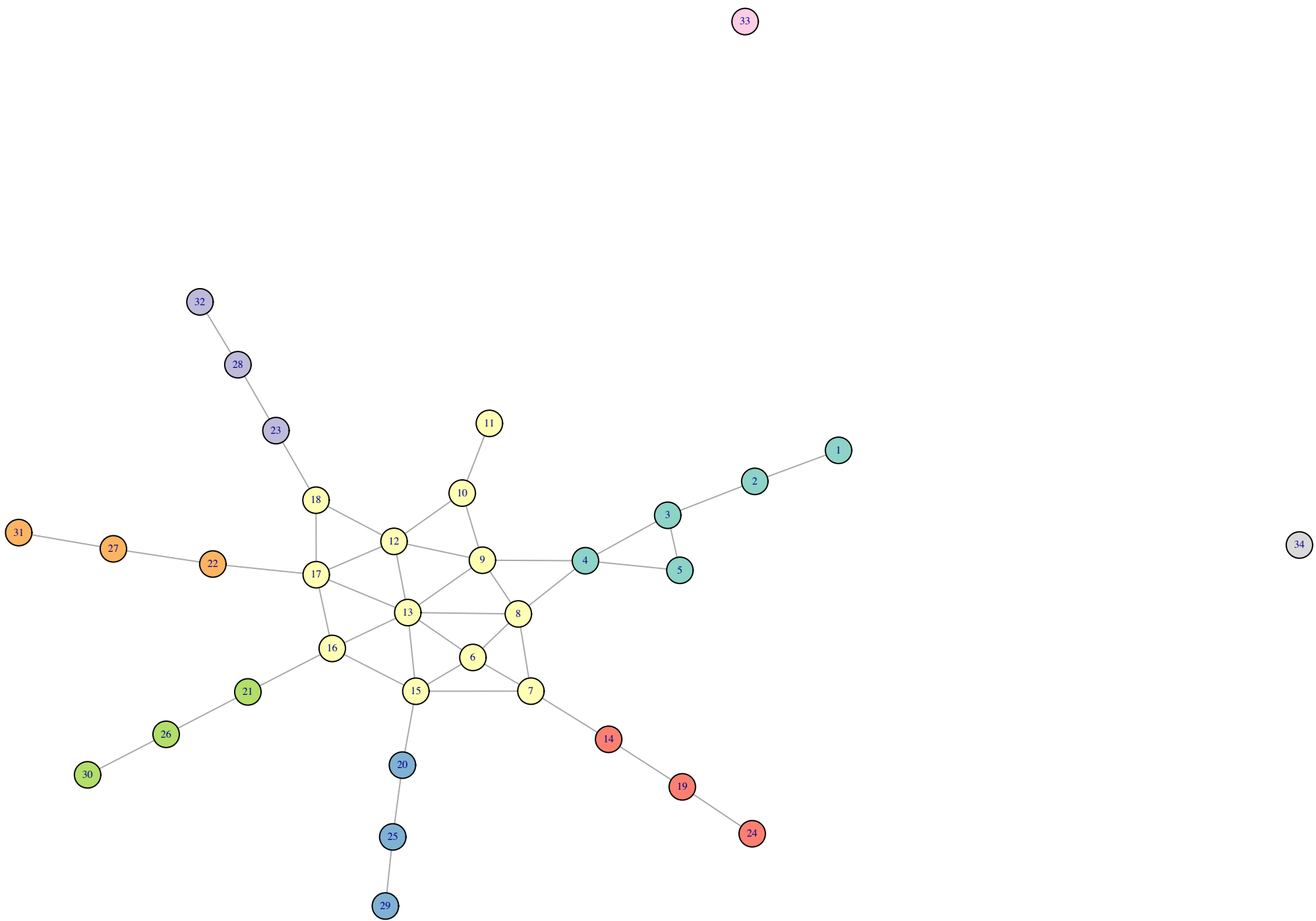

Supplement: S16 Fig — Legend idem than S1 Fig. (PDF) [file pone.0140030.s016.pdf]

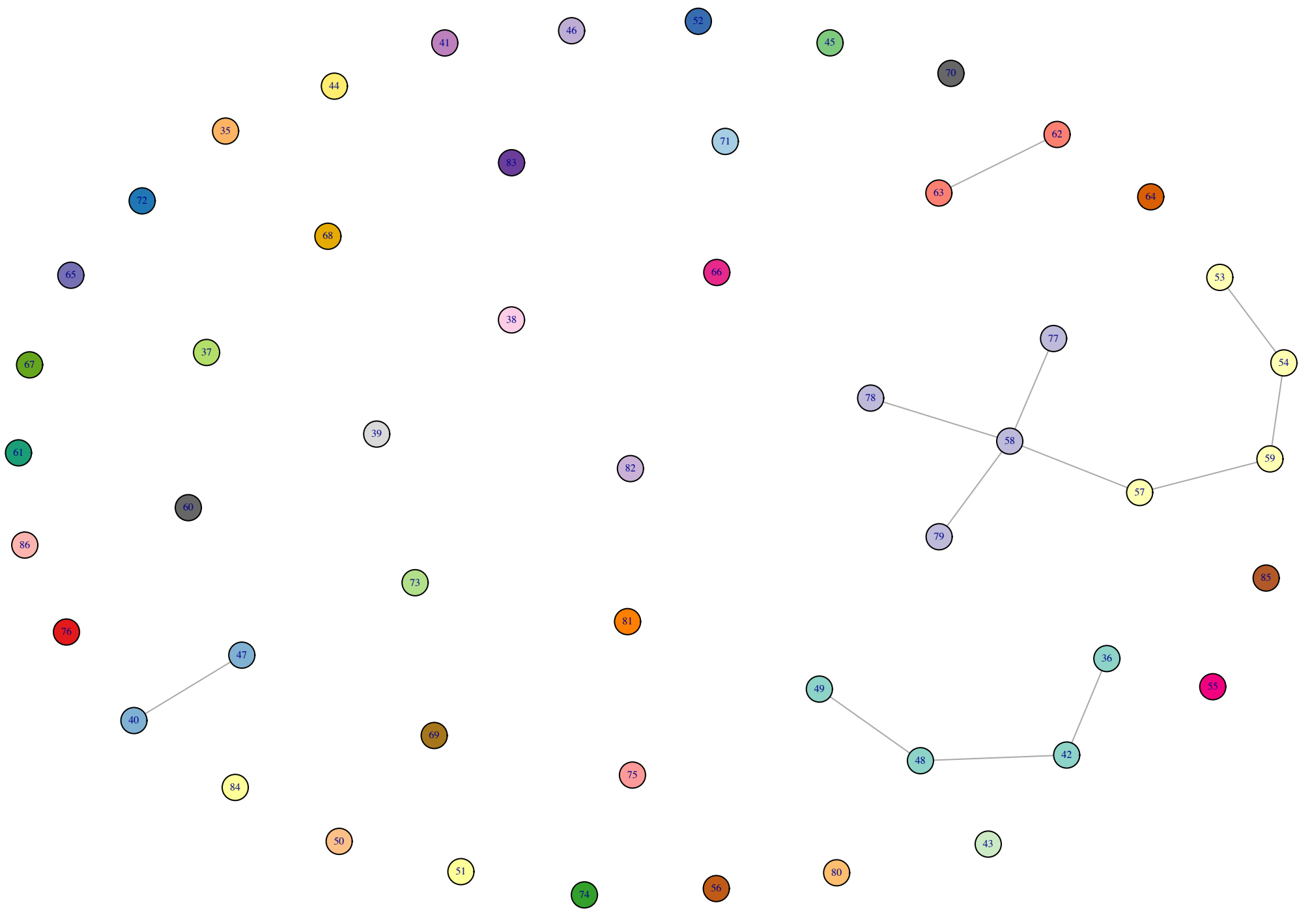

Supplement: S17 Fig — Legend idem than S1 Fig. (PDF) [file pone.0140030.s017.pdf]

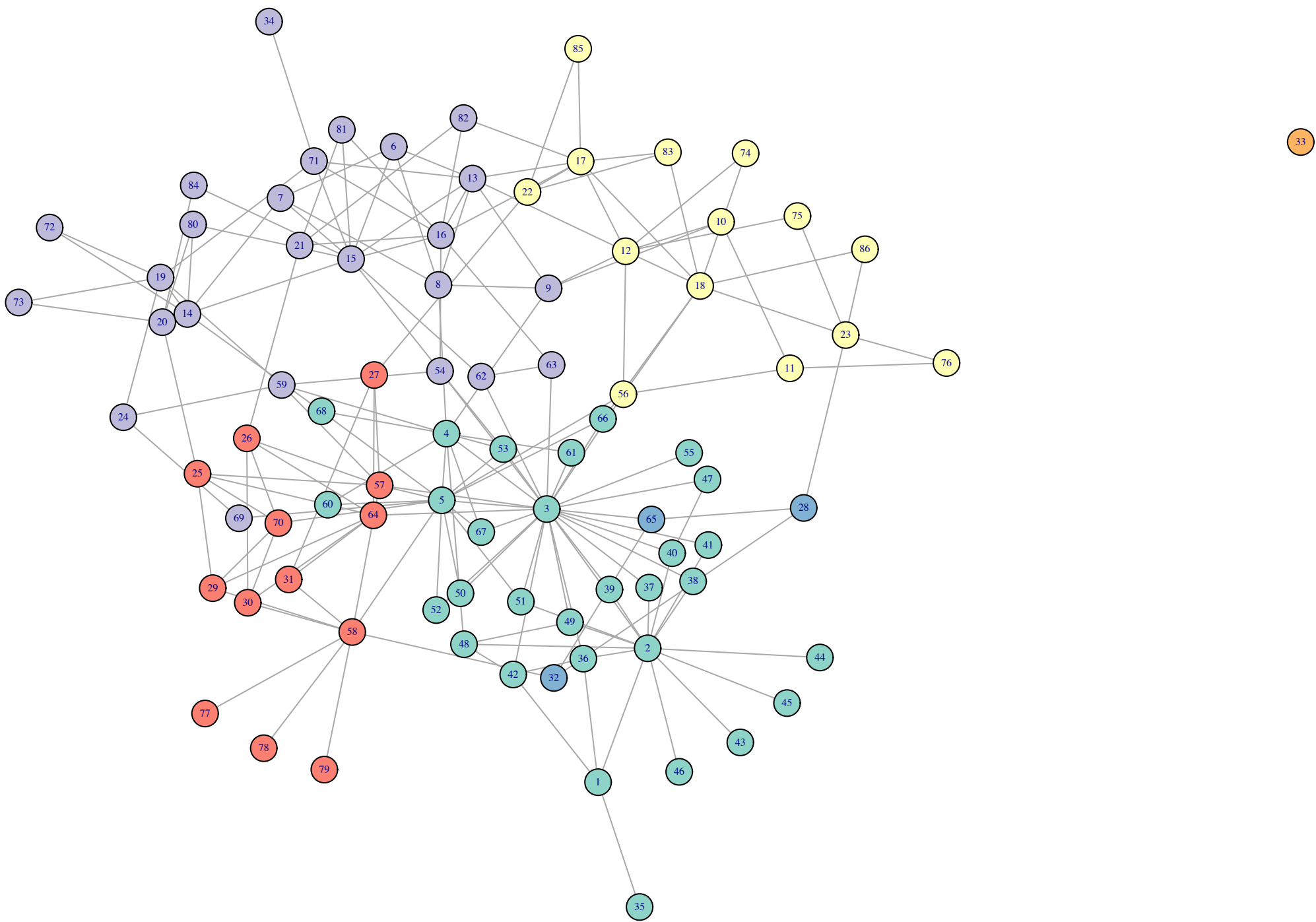

Supplement: S18 Fig — Legend idem than S1 Fig. (PDF) [file pone.0140030.s018.pdf]

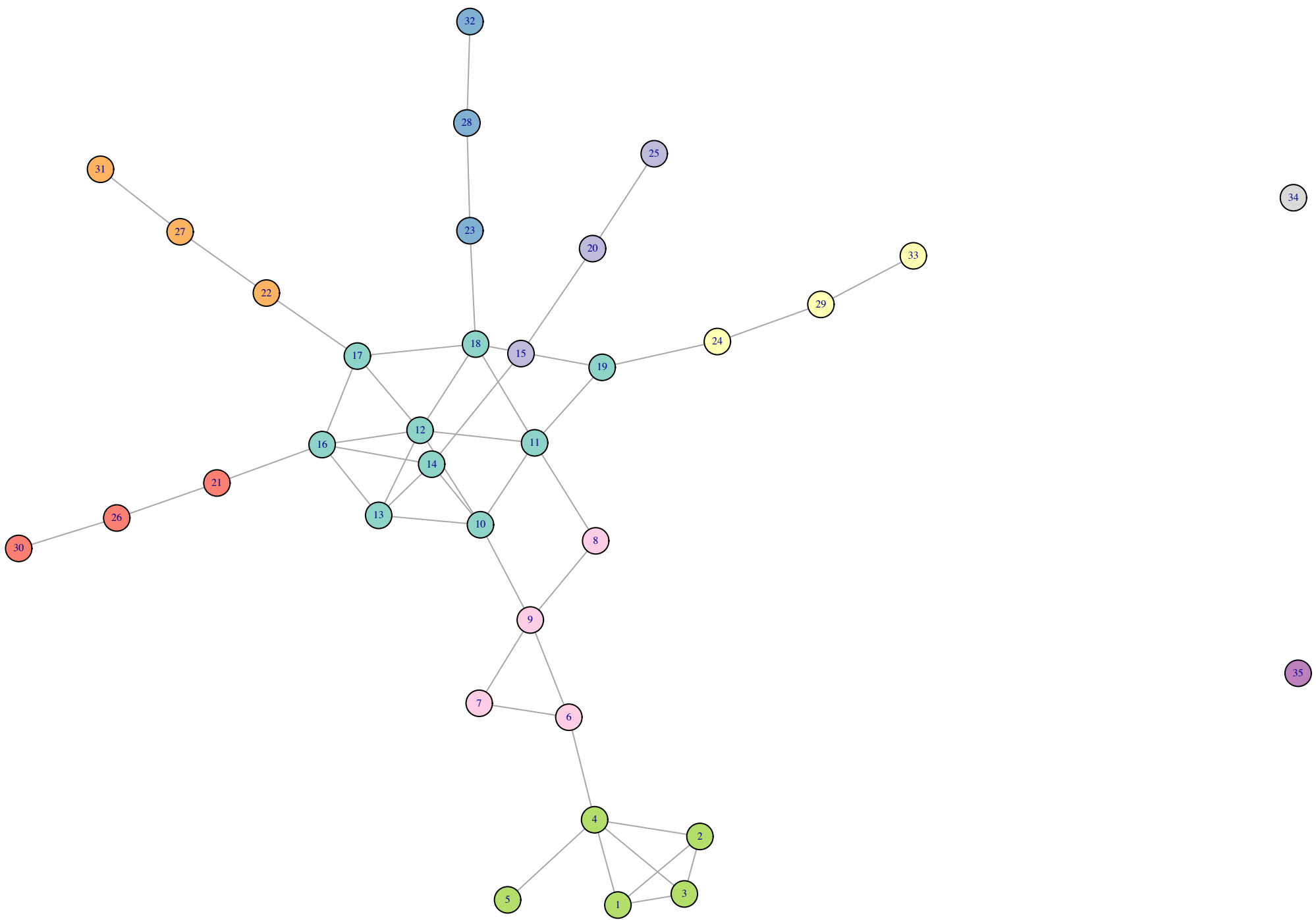

Supplement: S19 Fig — Legend idem than S1 Fig. (PDF) [file pone.0140030.s019.pdf]

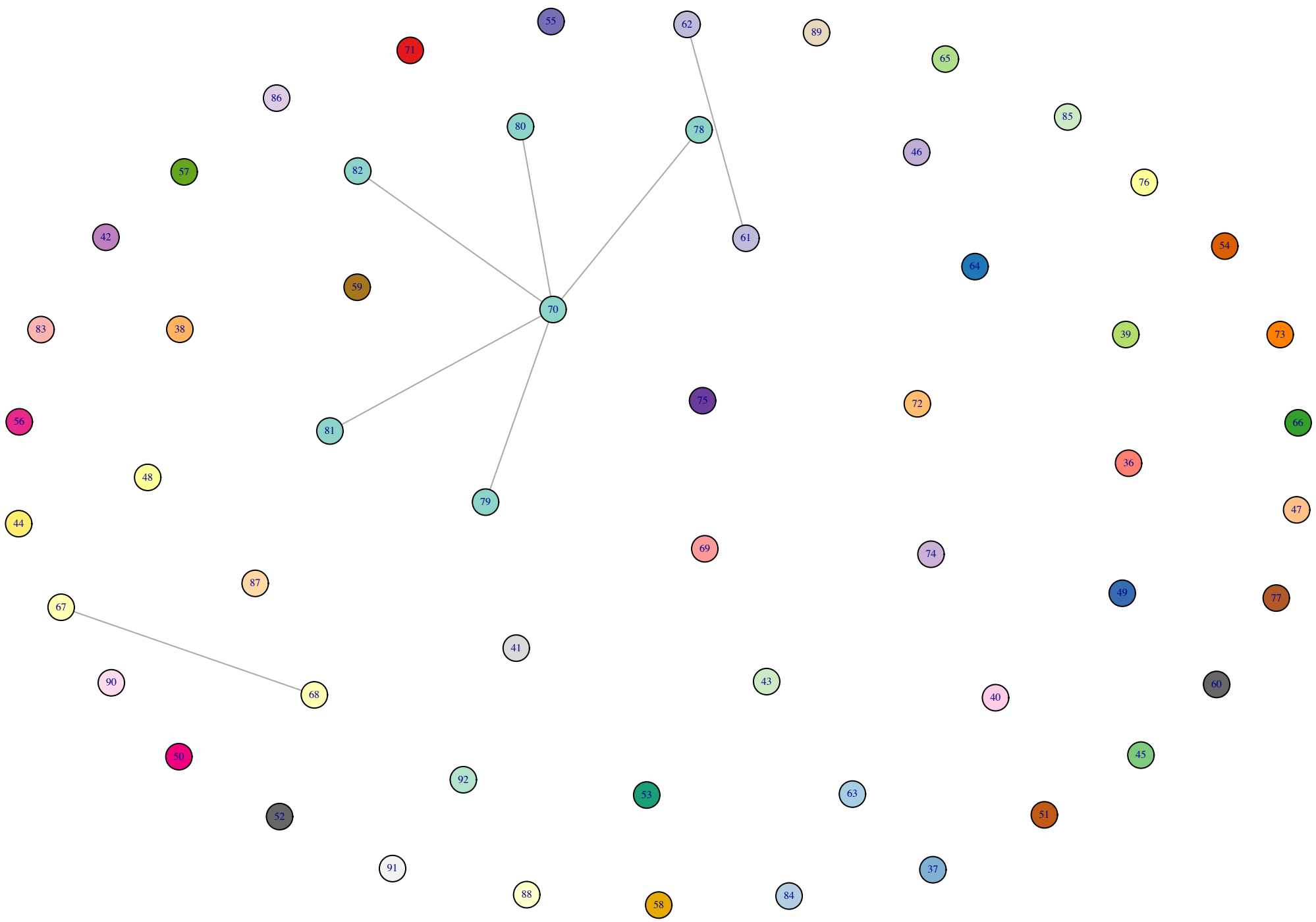

Supplement: S20 Fig — Legend idem than S1 Fig. (PDF) [file pone.0140030.s020.pdf]

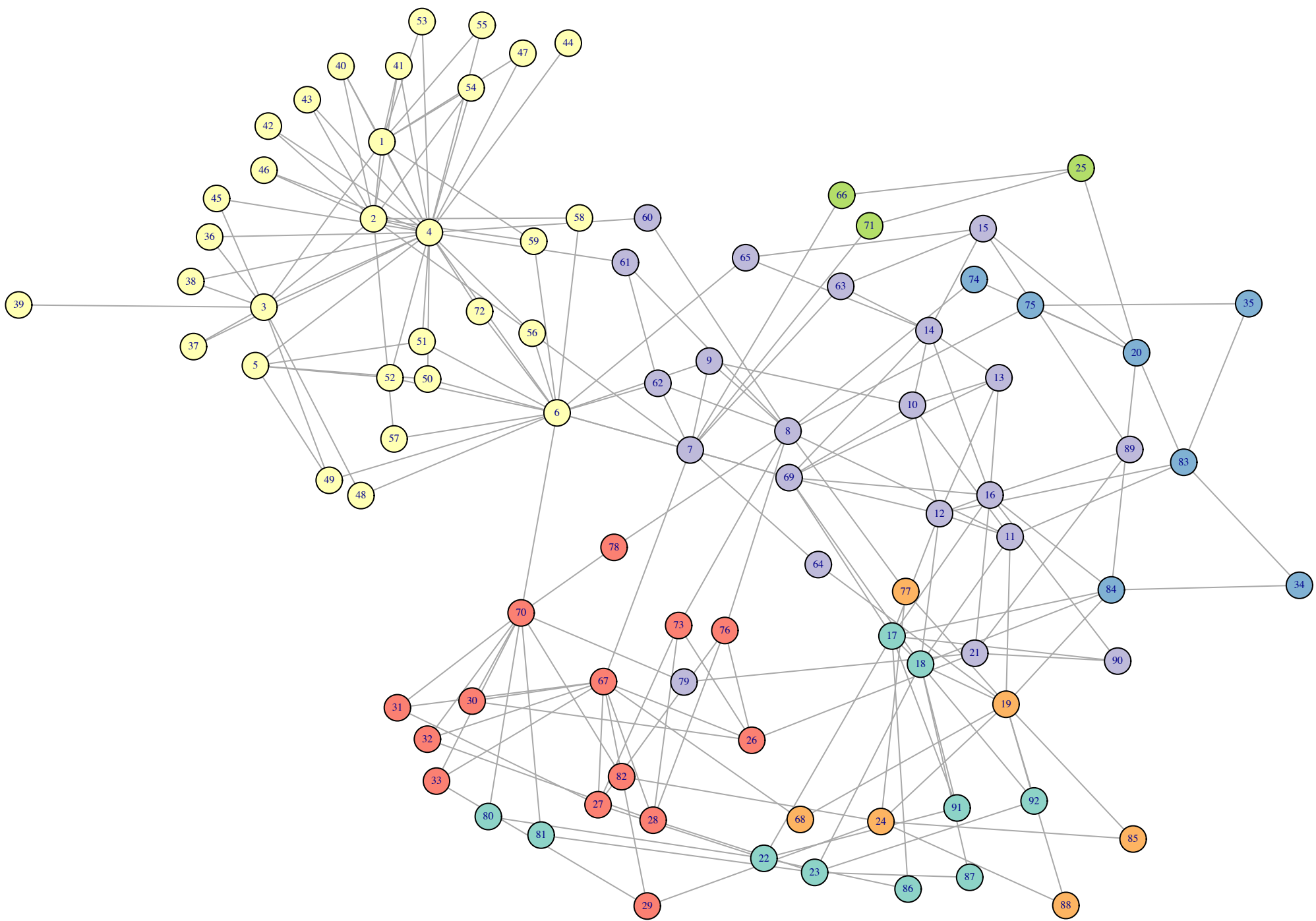

Supplement: S21 Fig — Legend idem than S1 Fig. (PDF) [file pone.0140030.s021.pdf]

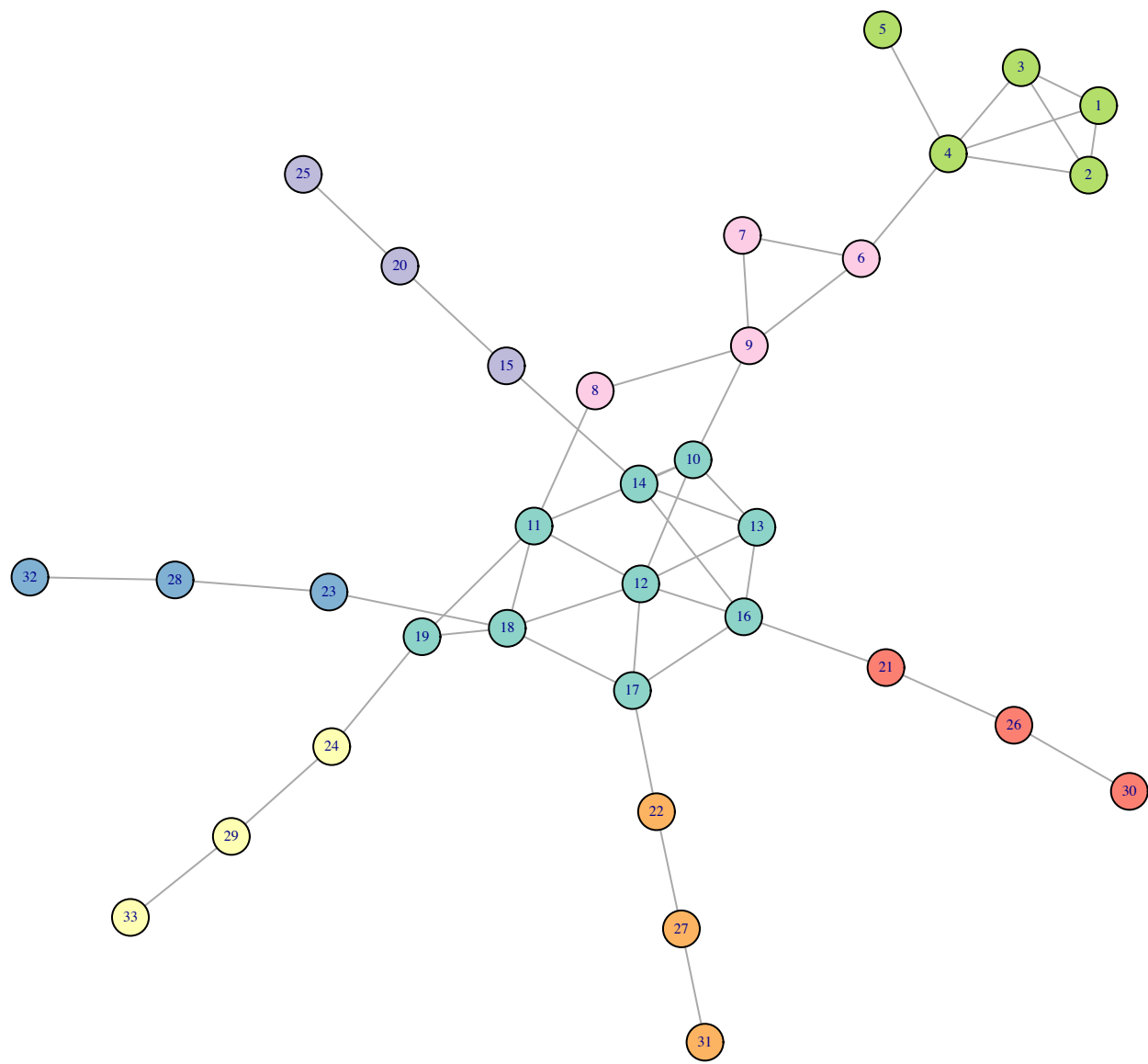

Supplement: S22 Fig — Legend idem than S1 Fig. (PDF) [file pone.0140030.s022.pdf]

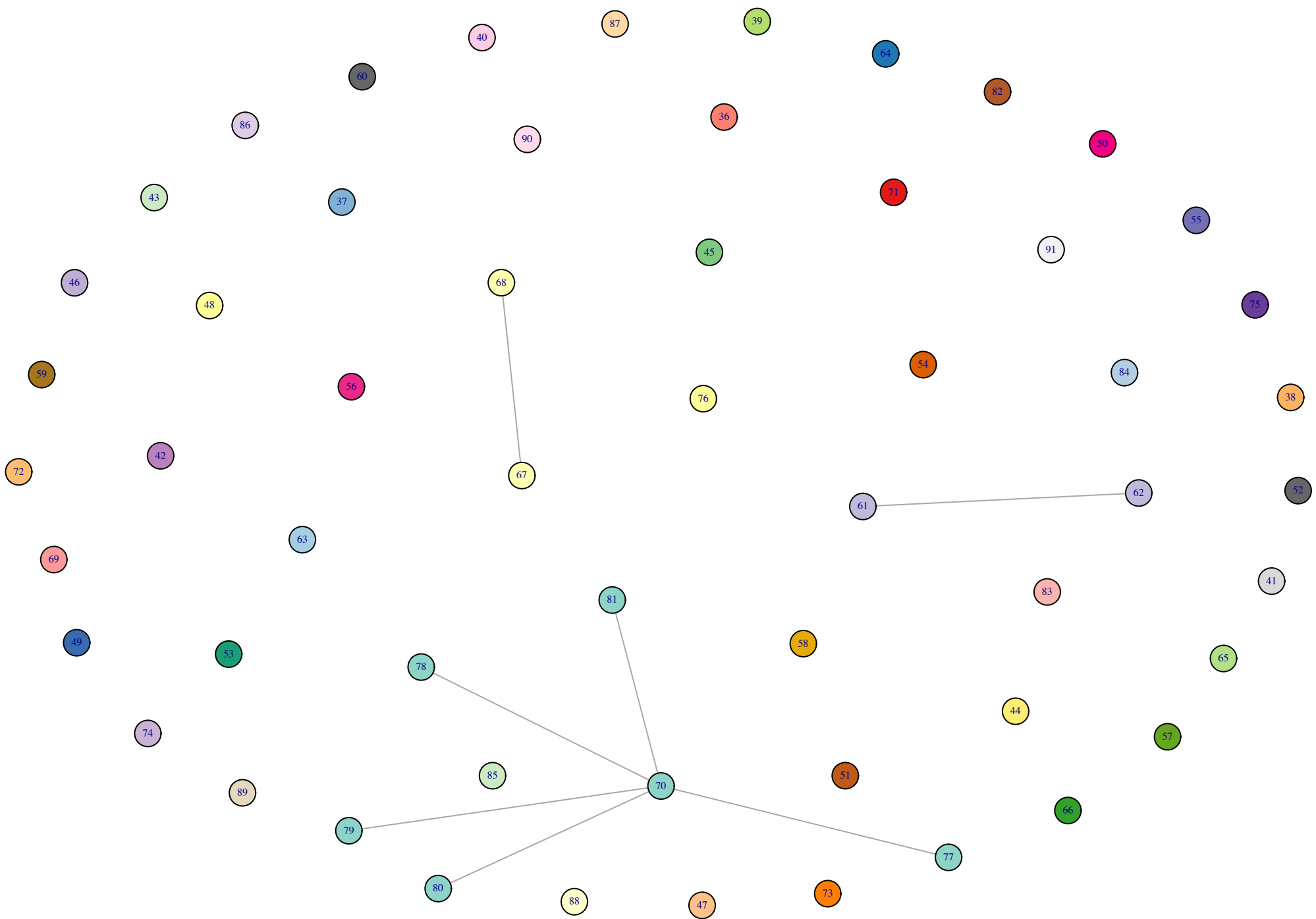

Supplement: S23 Fig — Legend idem than S1 Fig. (PDF) [file pone.0140030.s023.pdf]

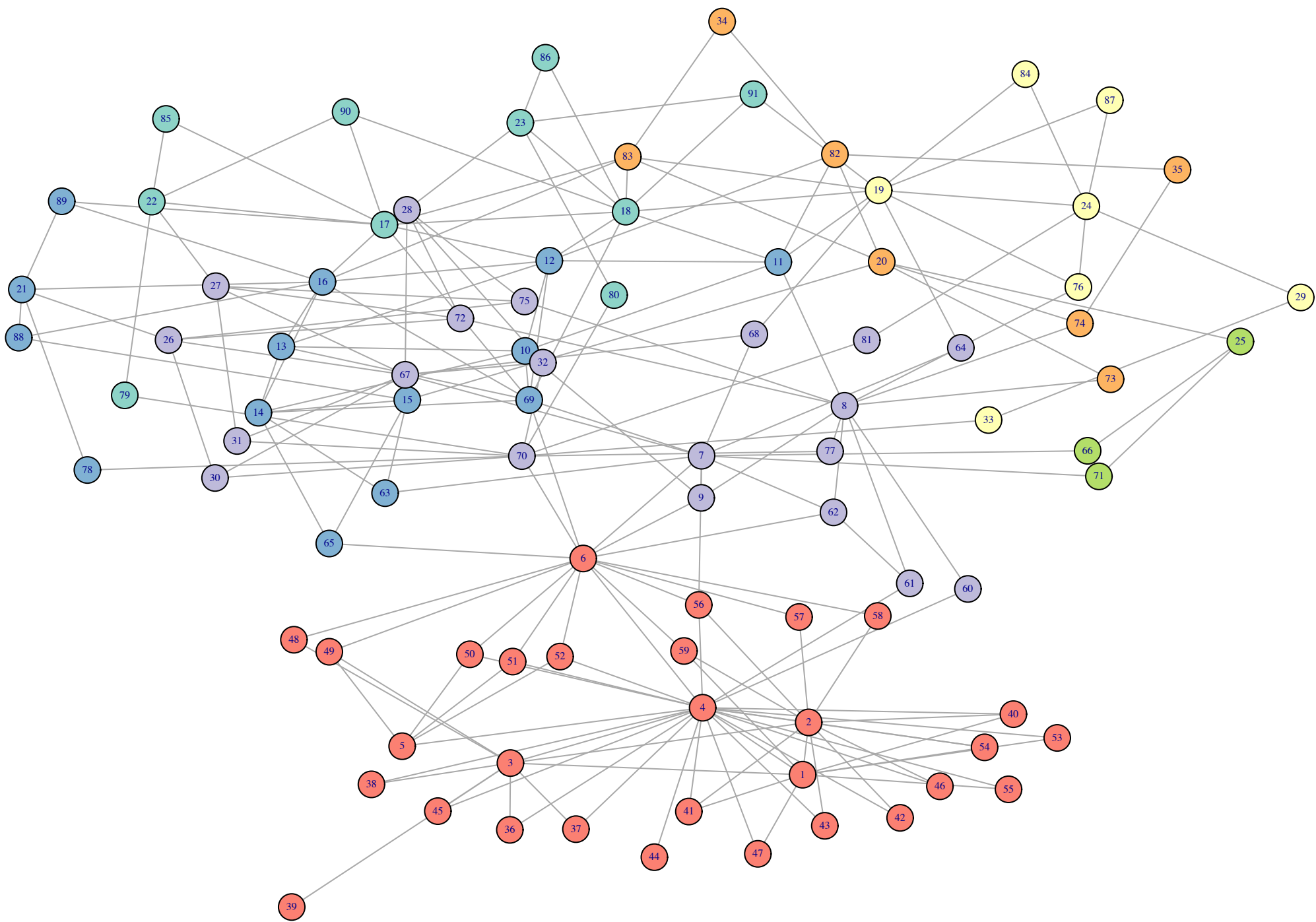

Supplement: S24 Fig — Legend idem than S1 Fig. (PDF) [file pone.0140030.s024.pdf]
