# Supplementary material for: Anatomical Network Comparison of Human Upper and Lower, Newborn and Adult, and Normal and Abnormal Limbs, with Notes on Development, Pathology and Limb Serial Homology vs. Homoplasy
Source: PLoS One. 2015 Oct 9;10(10):e0140030. doi: 10.1371/journal.pone.0140030 (PMC4599883; doi:10.1371/journal.pone.0140030)

Adult Lower Muscular Limb

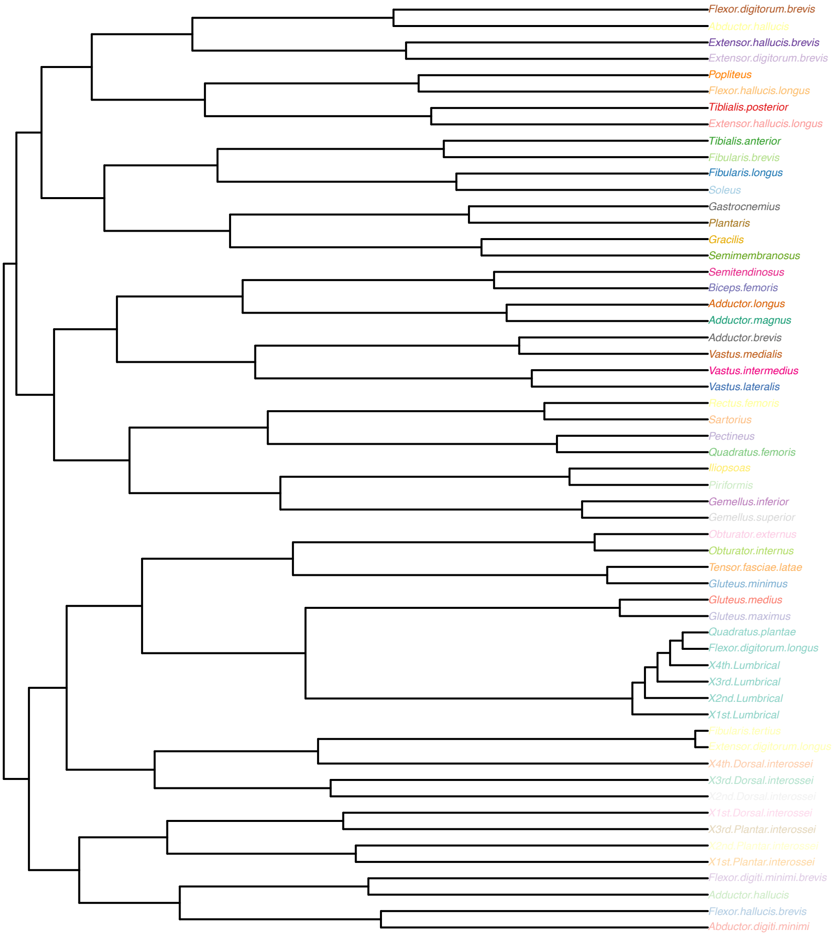

Adult Lower Musculoskeletal Limb

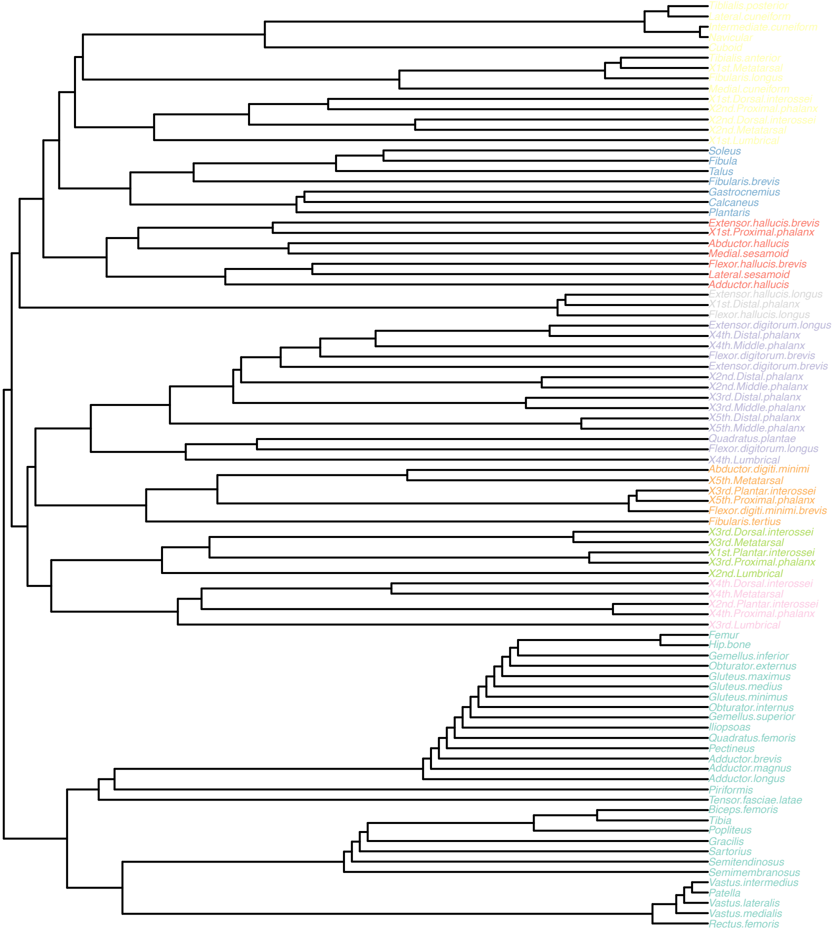

Adult Lower Skeletal Limb

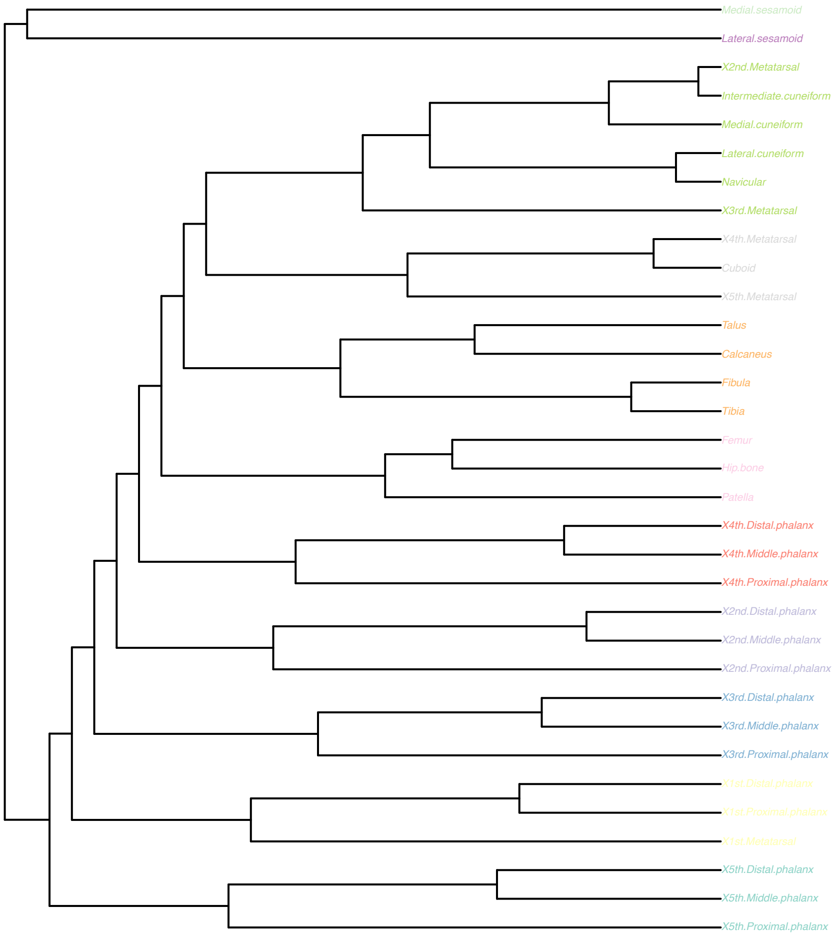

Adult & Newborn Upper Muscular Limb

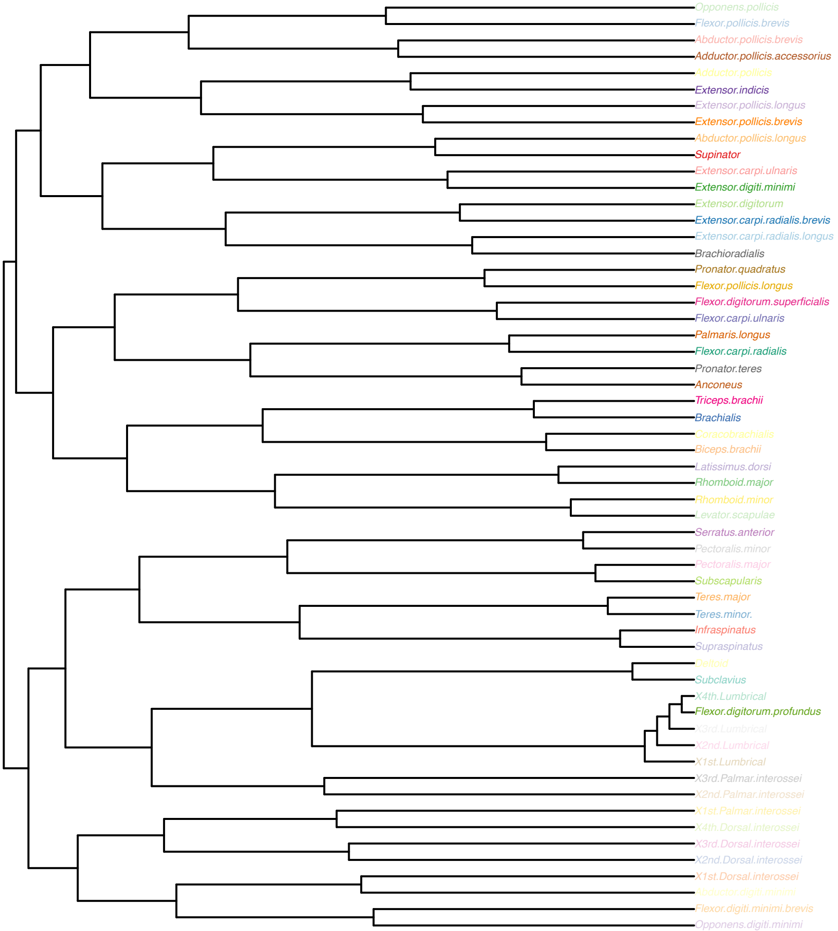

Adult & Newborn Upper Musculoskeletal Limb

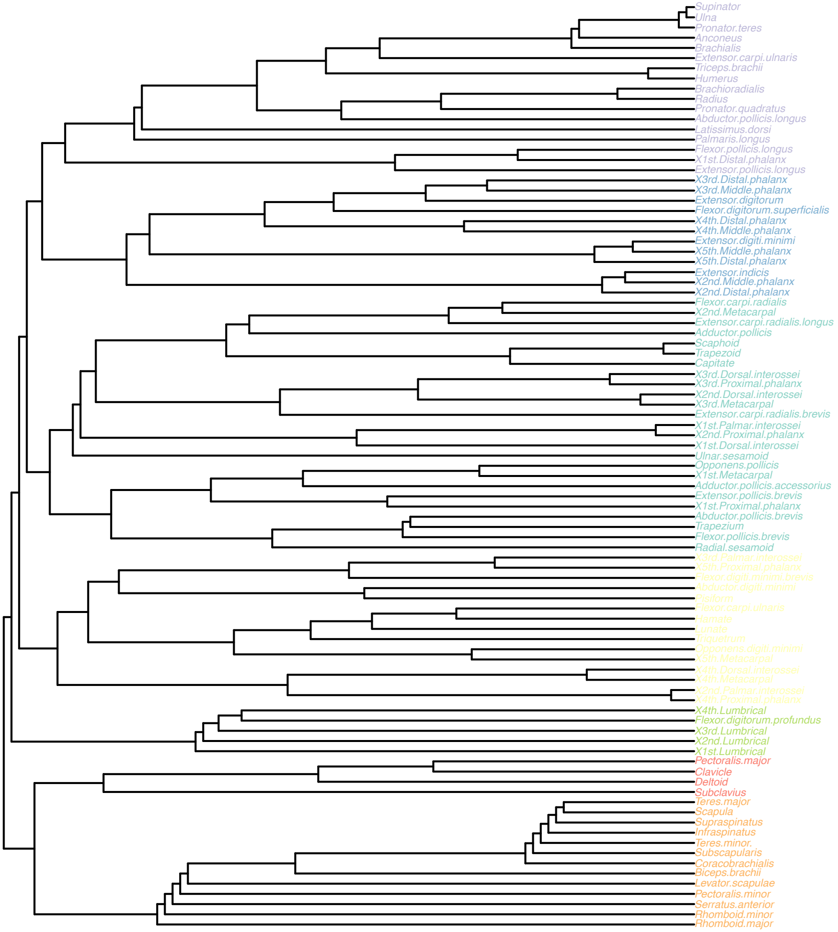

Adult & Newborn Upper Skeletal Limb

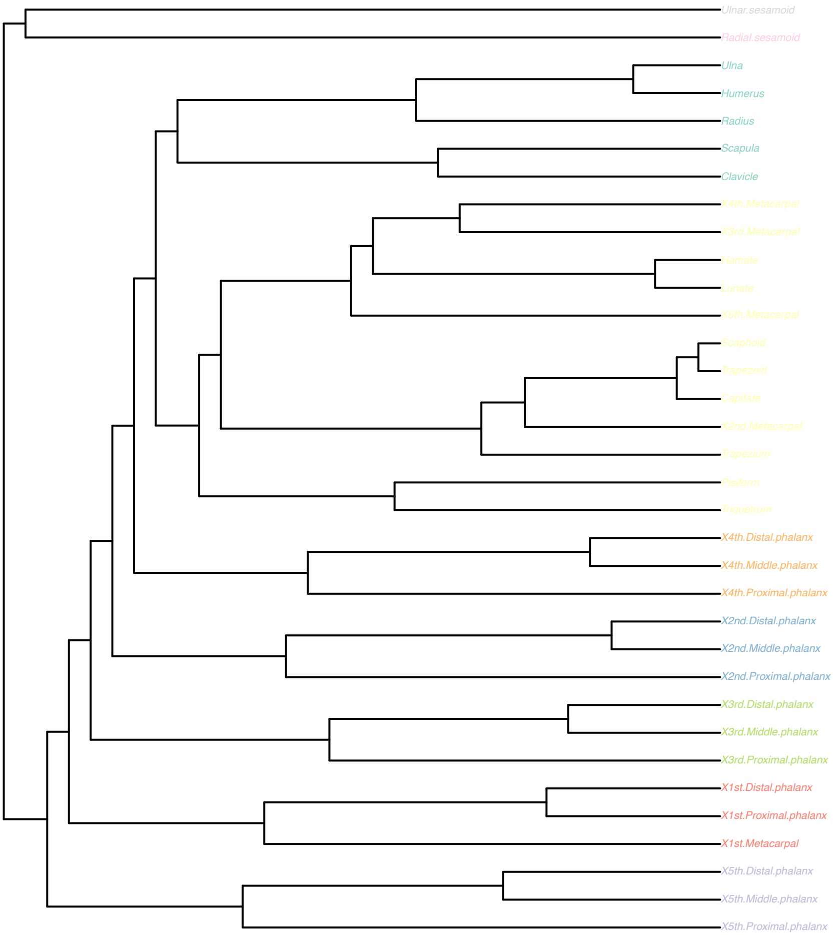

Newborn Lower Muscular Limb

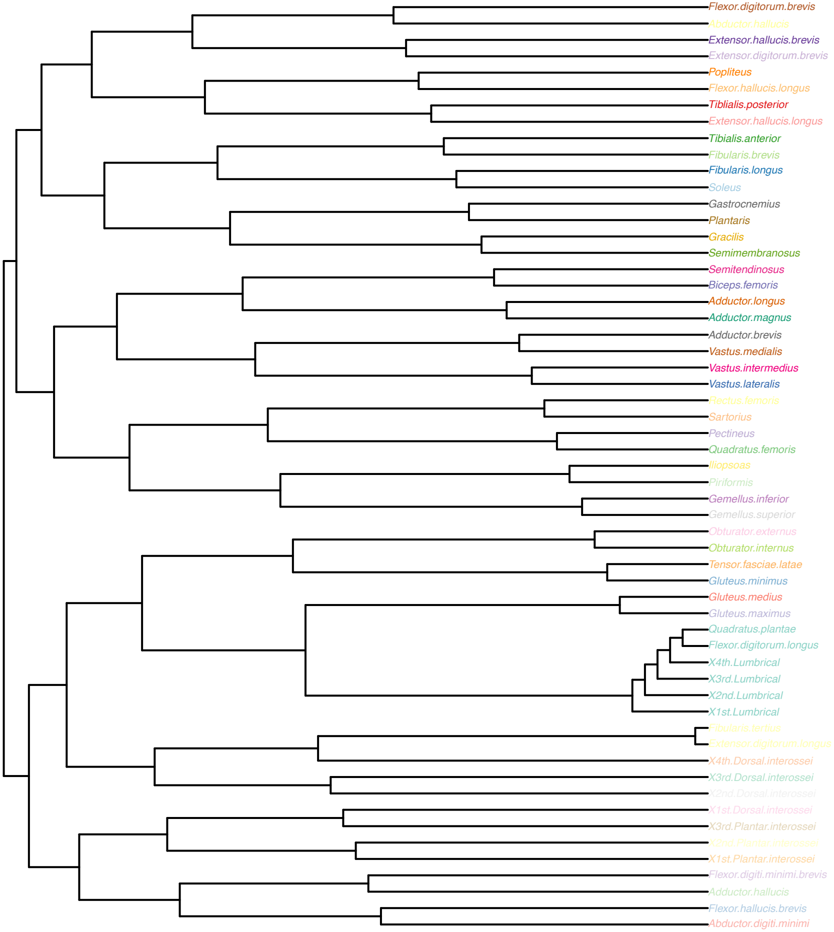

Newborn Lower Musculoskeletal Limb

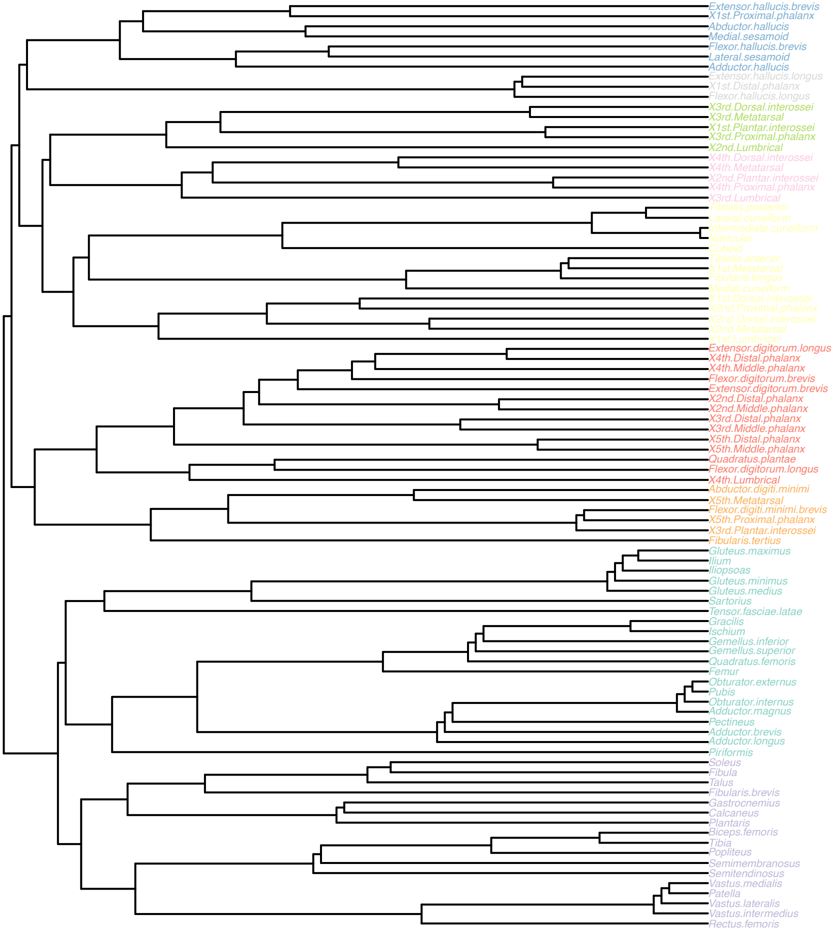

Newborn Lower Skeletal Limb

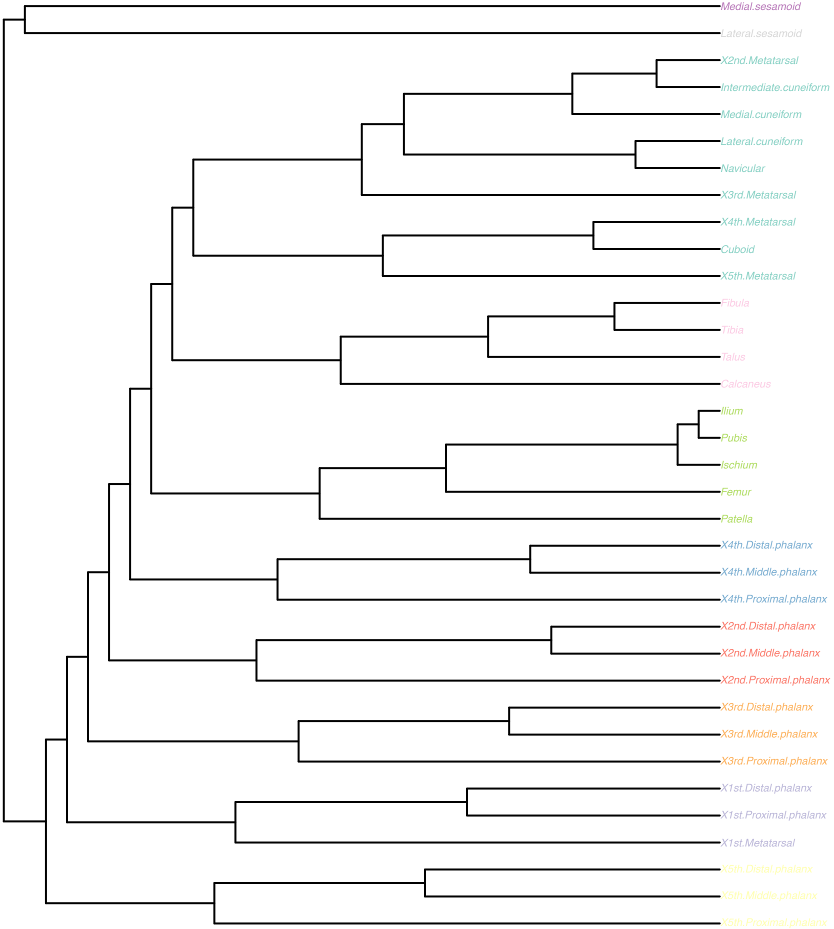

T18 Lower Left Muscular Limb

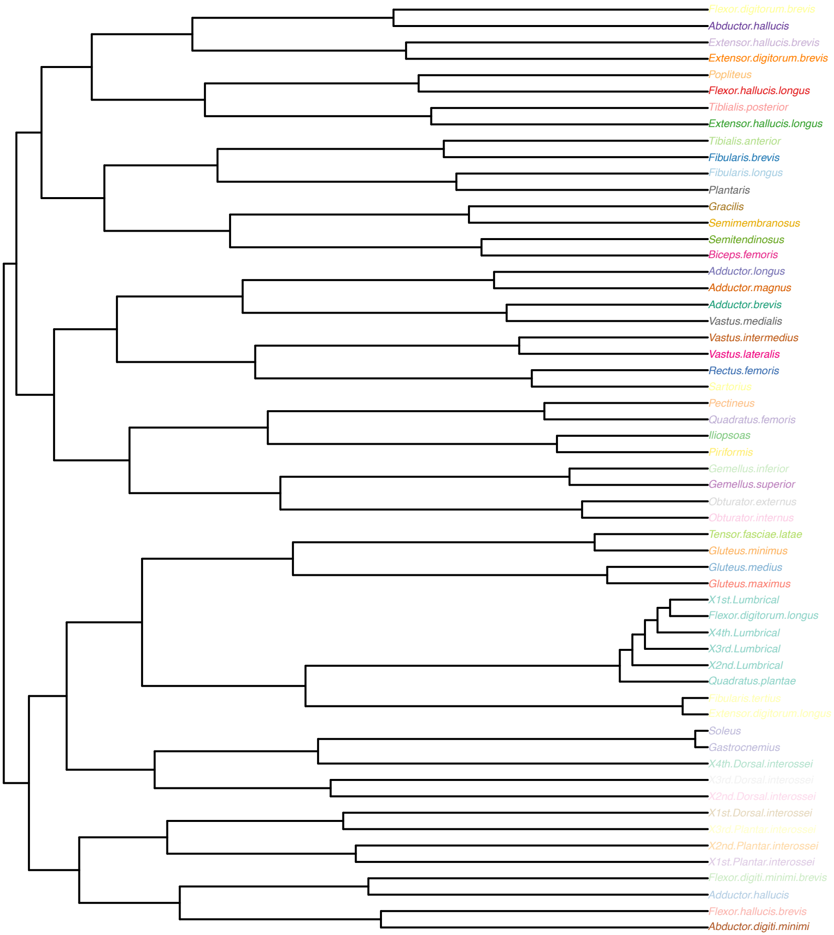

T18 Lower Left Musculoskeletal Limb

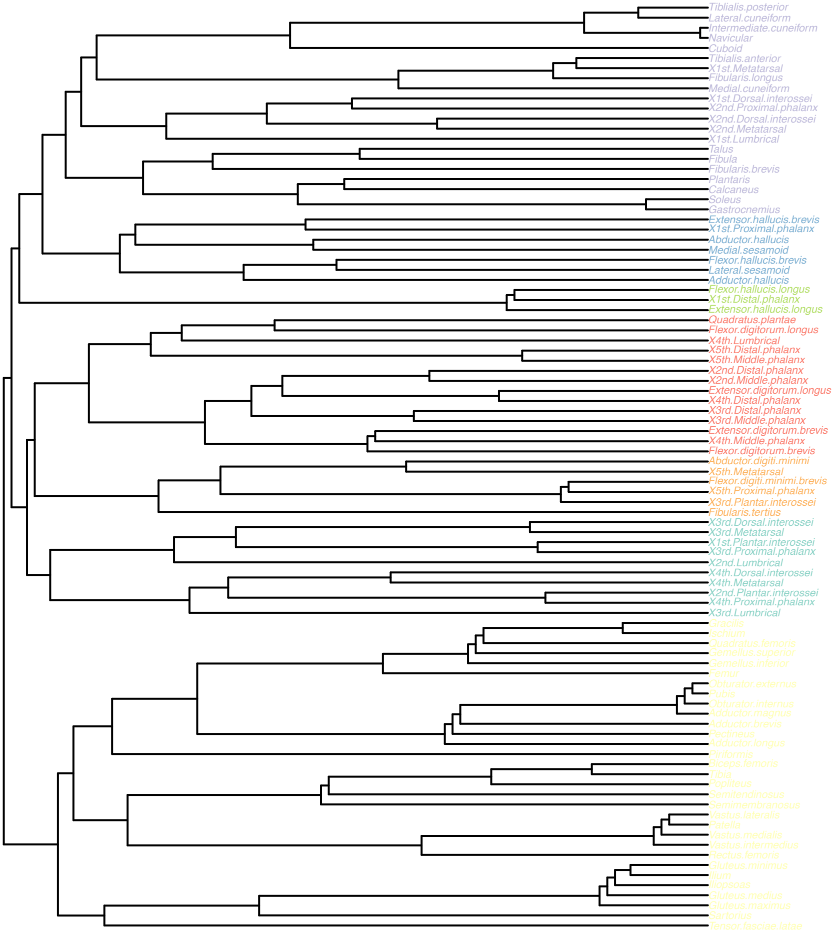

T18 Lower Left Skeletal Limb

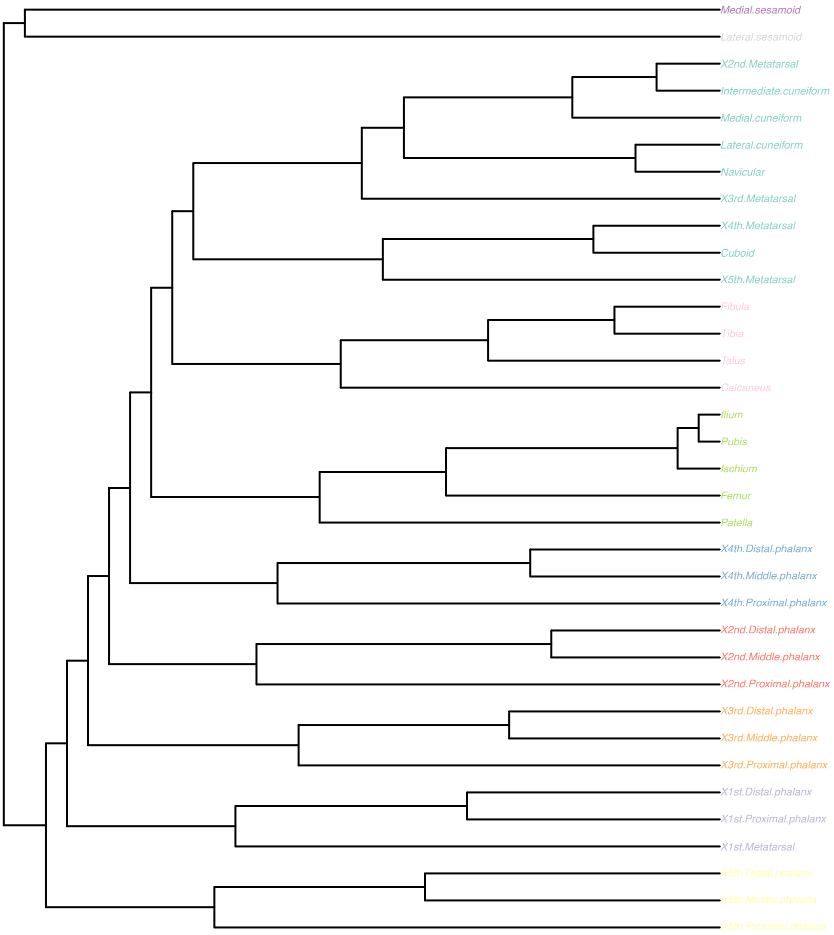

T18 Lower Right Muscular Limb

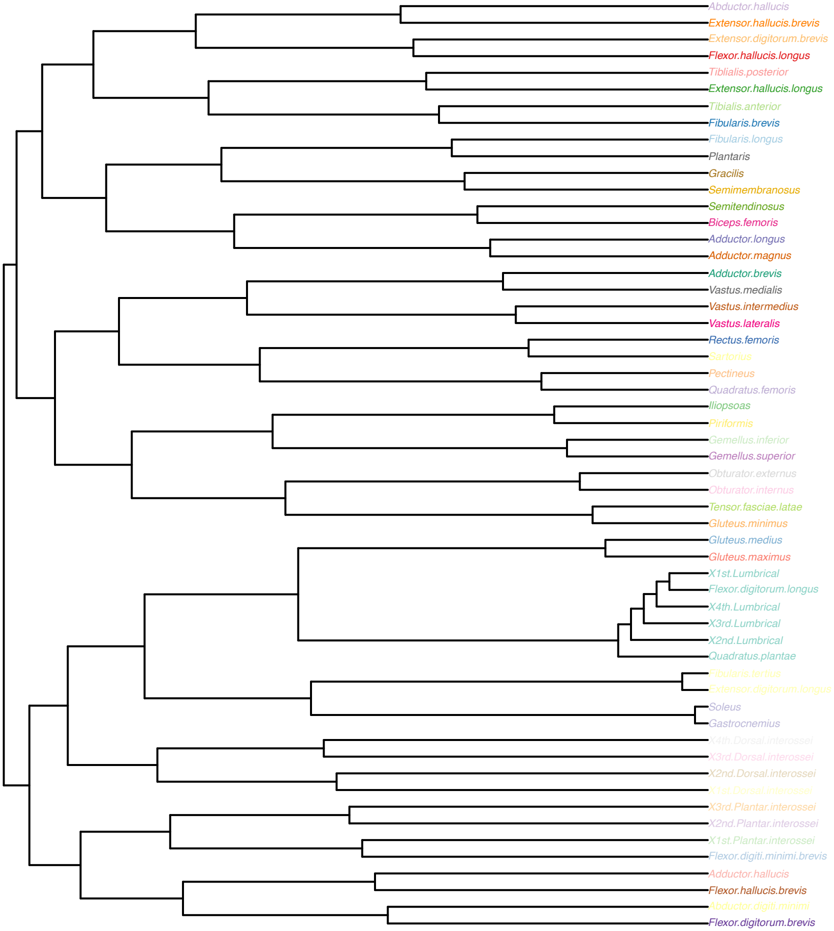

T18 Lower Right Musculoskeletal Limb

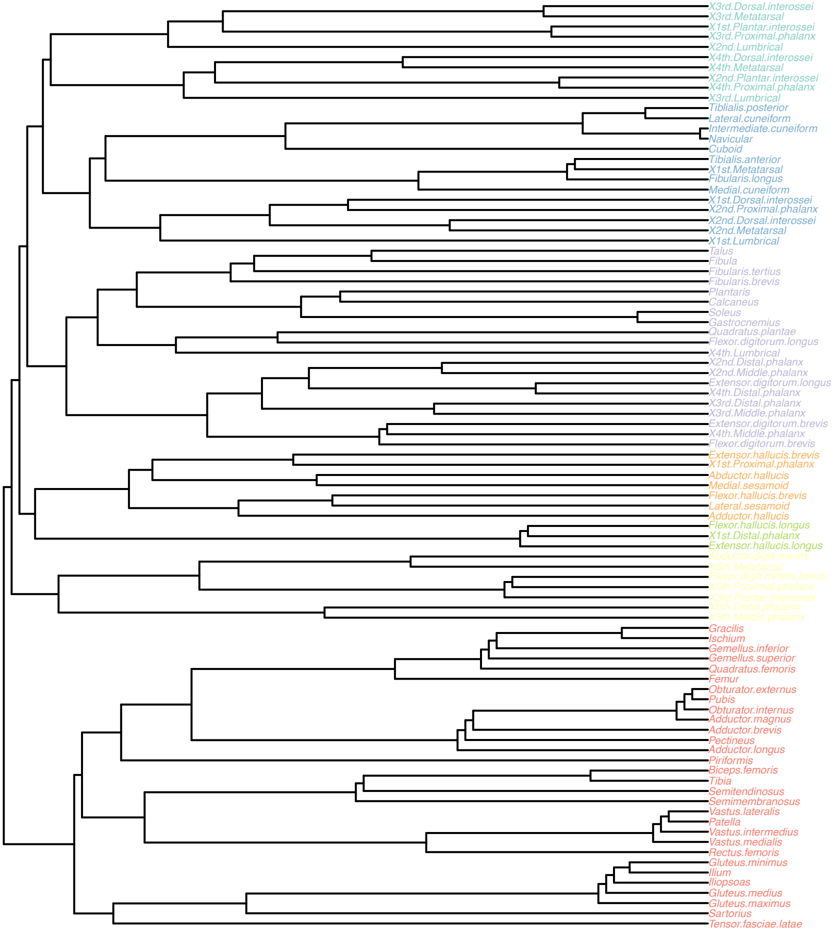

T18 Lower Right Skeletal Limb

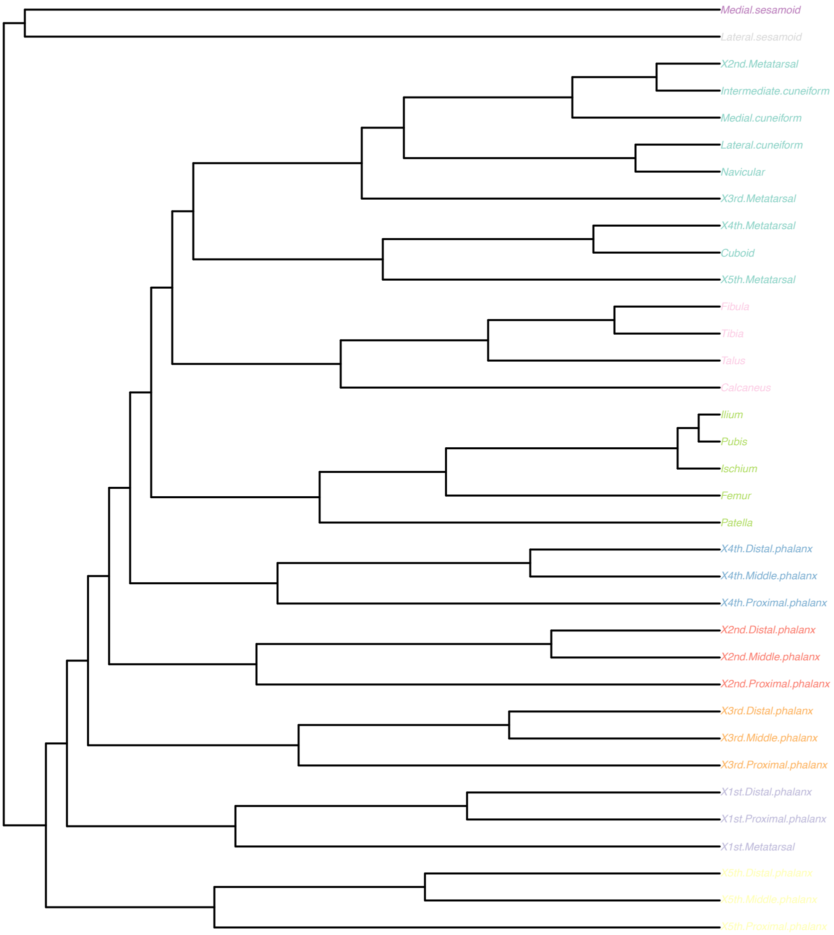

T18 Upper Left Muscular Limb

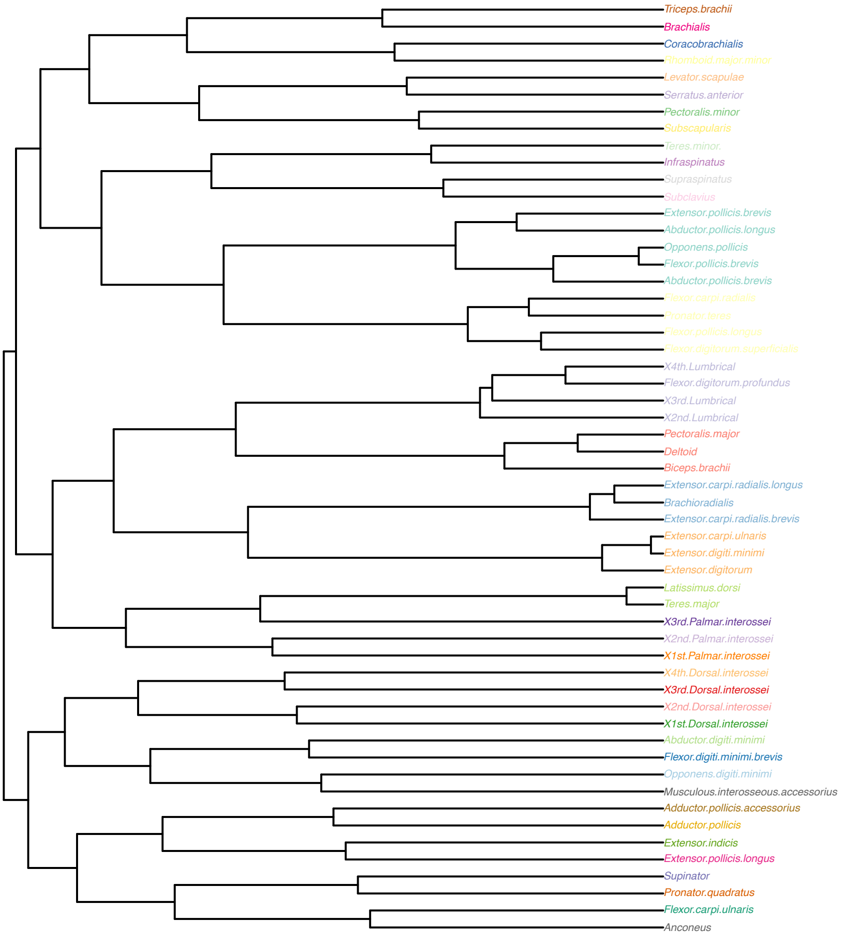

T18 Upper Left Musculoskeletal Limb

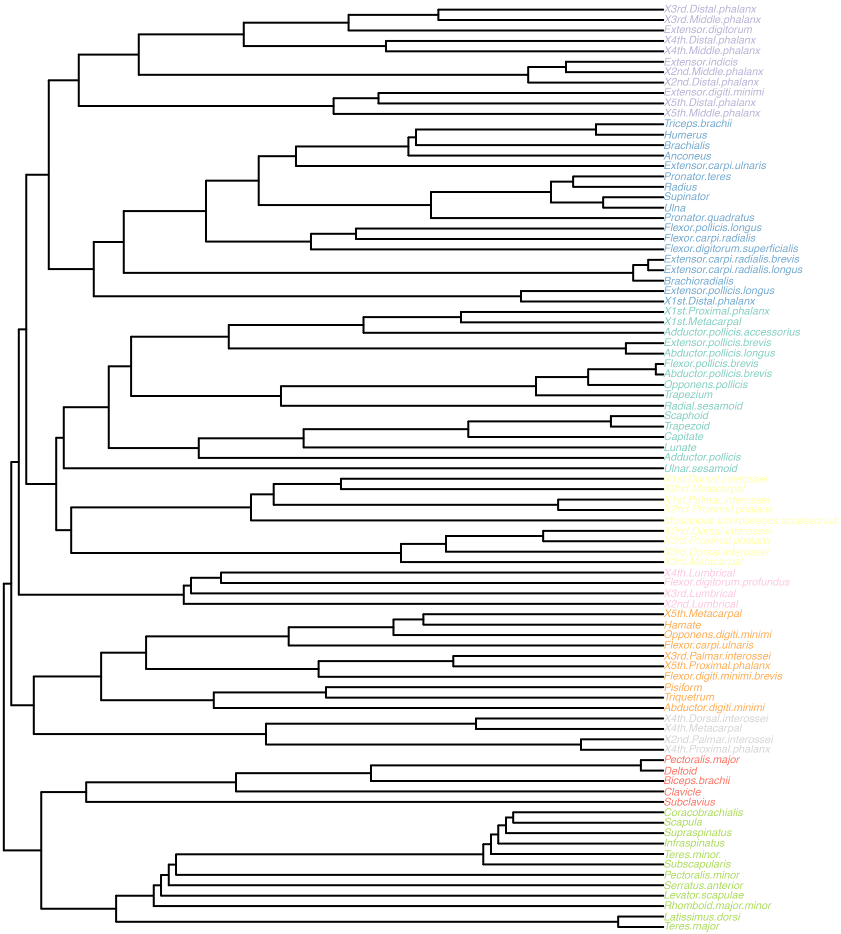

T18 Upper Left Skeletal Limb

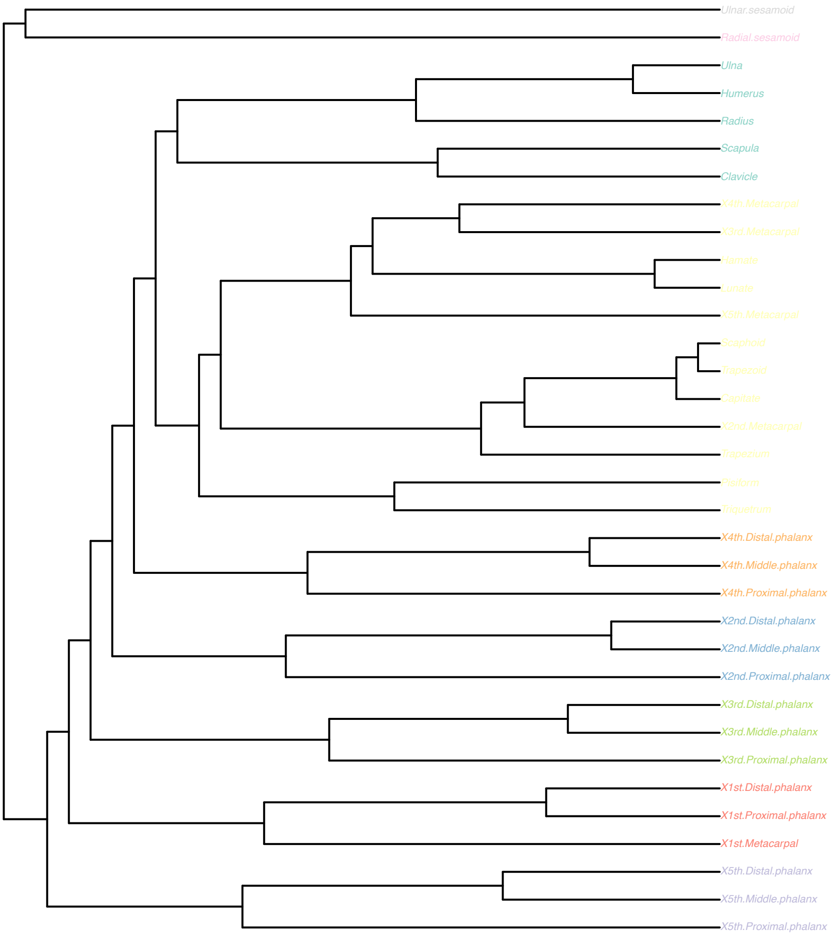

T18 Upper Right Muscular Limb

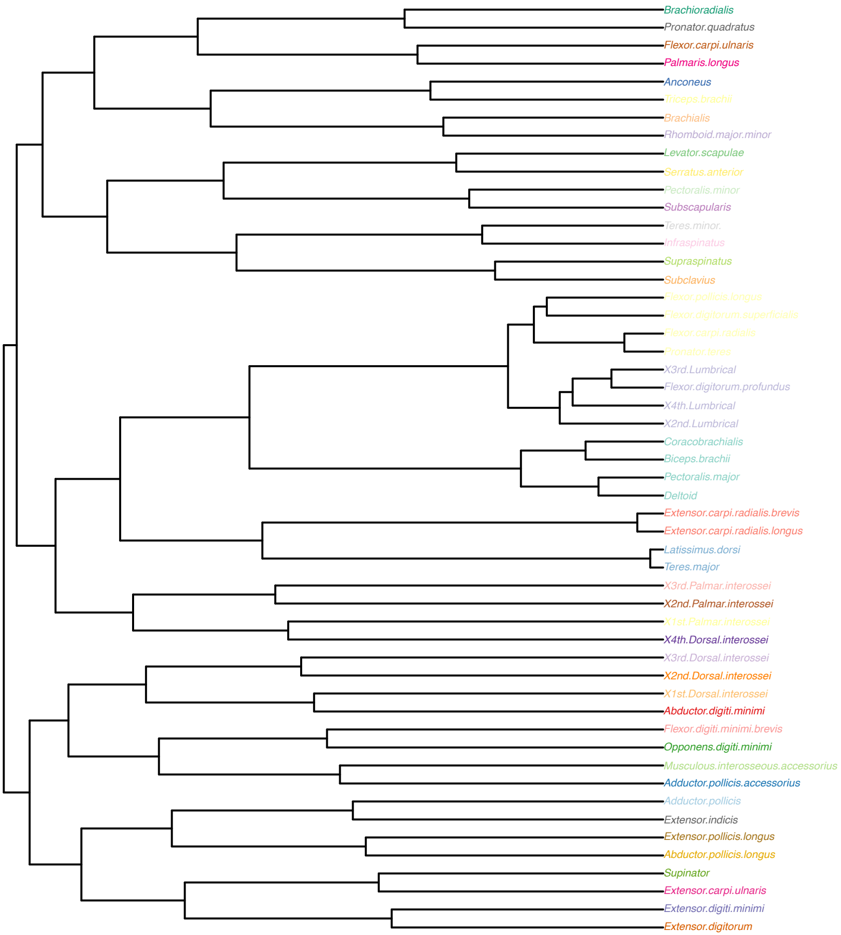

T18 Upper Right Musculoskeletal Limb

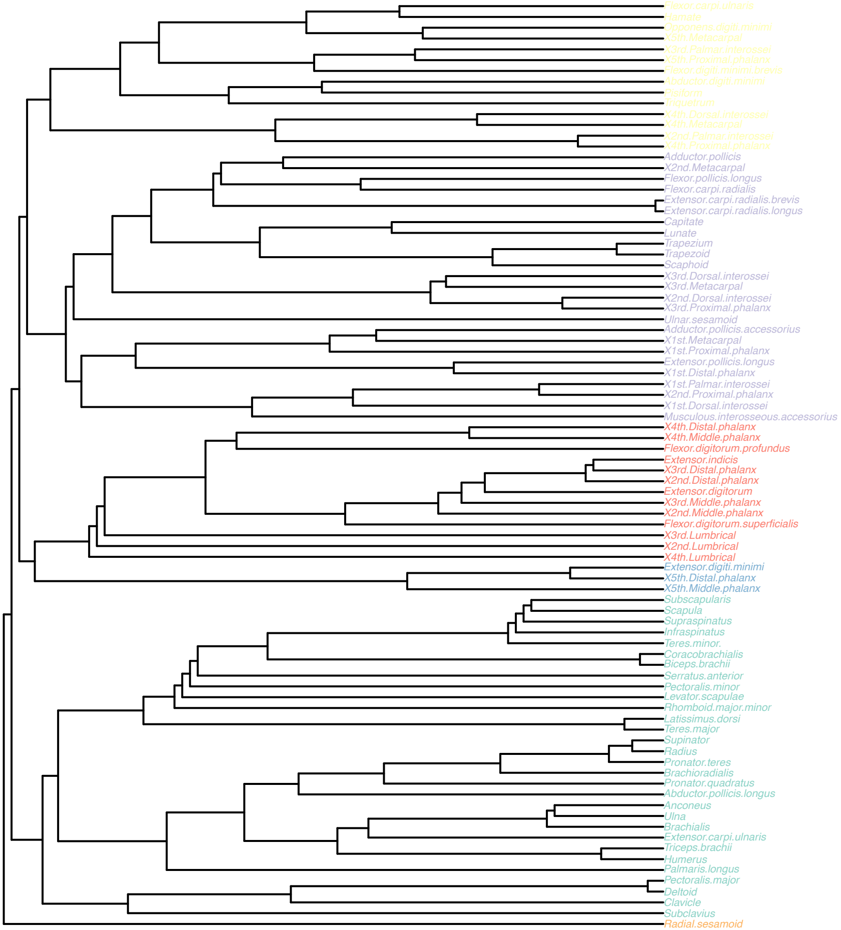

T18 Upper Right Skeletal Limb

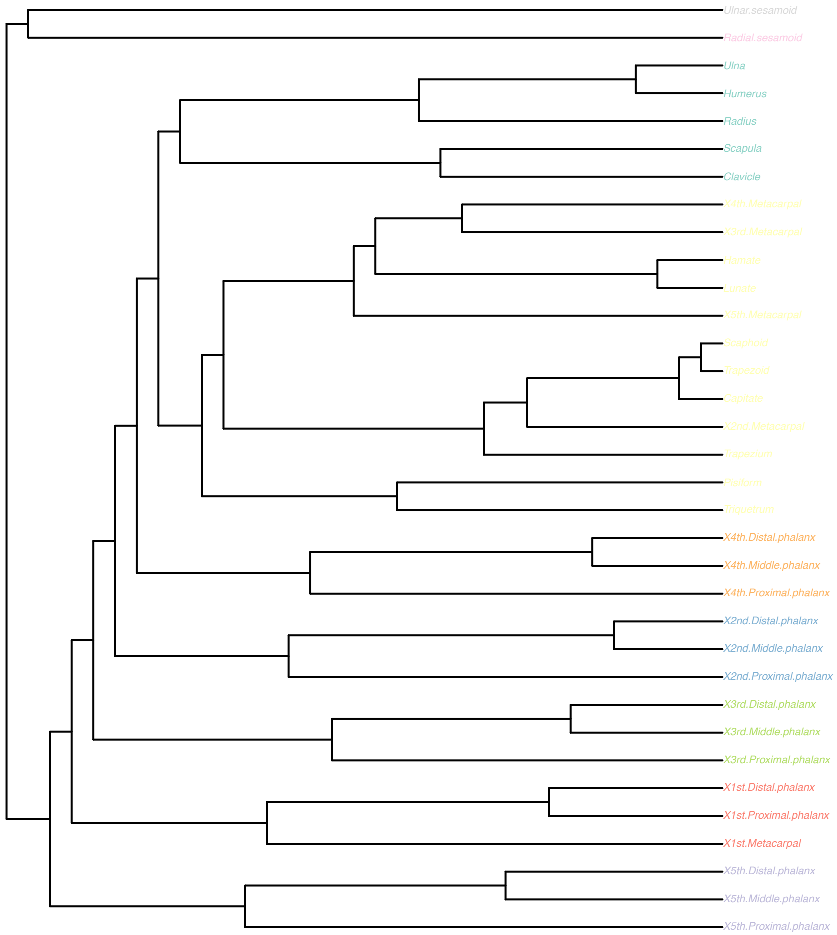

Supplement: S4 Methods — (PDF) [file pone.0140030.s028.pdf]
