# Supplementary material for: Anatomical Network Comparison of Human Upper and Lower, Newborn and Adult, and Normal and Abnormal Limbs, with Notes on Development, Pathology and Limb Serial Homology vs. Homoplasy
Source: PLoS One. 2015 Oct 9;10(10):e0140030. doi: 10.1371/journal.pone.0140030 (PMC4599883; doi:10.1371/journal.pone.0140030)
Supplement: S5 Methods — (DOCX) [file pone.0140030.s029.docx]

**SI.FunDev: Functional and developmental groups for upper and lower limbs (UL, LL)*.**

***For each table, different numbers are used for different groups.**

**Developmental and functional groups of the bones of the upper limb.**

| **Supragroup** | **Group** | **Subgroup** | **Bones** |
| --- | --- | --- | --- |
| Girdle | Girdle |  | **1** Scapula, Clavicle |
| Arm | Stylopod |  | **2** Humerus |
|  | Zeugopod |  | **3** Ulna, Radius |
| Hand | Autopod | Wrist | **4** Trapezoid, Trapezium, Scaphoid, Lunate, Triquetrum, Pisiform, Hamate, Capitate |
|  |  | Digit I | **5** 1st Metacarpal, Proximal phalanx, Distal phalanx, Radial sesamoid, Ulnar sesamoid |
|  |  | Digit II | **6** 2st Metacarpal, Proximal phalanx, Distal phalanx, Middle phalanx |
|  |  | Digit III | **7** 3st Metacarpal, Proximal phalanx, Distal phalanx, Middle phalanx |
|  |  | Digit IV | **8** 4st Metacarpal, Proximal phalanx, Distal phalanx, Middle phalanx |
|  |  | Digit V | **9** 5st Metacarpal, Proximal phalanx, Distal phalanx, Middle phalanx |

**Developmental groups of muscles of the upper limb.**

| **Supragroup** | **Group** | **Subgroup** | **Muscles** |
| --- | --- | --- | --- |
| Girdle | Primaxial musculature |  | **1** Levator scapulae, Rhomboid major, Rhomboid minor |
|  |  |  | **1** Serratus anterior |
|  | Abaxial pectoral musculature |  | **2** Subscapularis, Teres major, Latissimus dorsi |
|  |  |  | **2** Infraspinatus, supraspinatus |
|  |  |  | **2** Deltoid, teres minor |
|  |  |  | **2** Pectoralis major, pectoralis minor |
|  |  |  | **2** Subclavius |
| Arm | Ventral muscles |  | **3** Biceps brachii, Coracobrachialis, Brachialis |
|  | Dorsal muscles |  | **4** Triceps brachii |
| Arm | Ventral muscles | Superficial | **5** Pronator teres, Flexor carpi radialis, Palmaris longus, Flexor digitorum superficialis, Flexor carpi ulnaris |
|  |  | Deep | **6** Pronator quadratus, Flexor pollicis longus, Flexor digitorum profundus |
|  | Dorsal muscles | Superficial | **7** Anconeus, Brachioradialis, Extensor carpi radialis longus, Extensor carpi radialis brevis, Extensor digitorum, Extensor digiti minimi, Extensor carpi ulnaris |
|  |  | Deep | **8** Supinator, Abductor pollicis longus, Extensor pollicis brevis, Extensor pollicis longus, Extensor indicis |
| Hand | Hand muscles |  | **9** Lumbricals 1, 2, 3, 4 |
|  |  |  | **10** Abductor pollicis brevis |
|  |  |  | **11** Adductor pollicis, Adductor pollicis accessorius |
|  |  | Flexores breves profundi group | **12** Flexor pollicis brevis, opponens pollicis, opponens digiti minimi, palmar interossei, dorsal interossei, flexor digiti minimi brevis |
|  |  |  | **13** Abductor digit minimi |

**Functional groups of muscles of the upper limb.**

| **Supragroup** | **Group** | **Subgroup** | **Muscles** |
| --- | --- | --- | --- |
| Girdle | Girdle movers |  | **1** Levator scapulae, Rhomboid major, Rhomboid minor, Pectoralis minor, Serratus anterior, Subclavius |
| Arm | Humerus movers | Flexors | **2** Pectoralis major, Coracobrachialis |
|  |  | Extensors | **2** Latissimus dorsi |
|  |  | Abductors | **2** Deltoid, Supraspinatus |
|  |  | Rotators | **2** Subscapularis, Teres major, Teres minor, Infraspinatus |
|  | Forearm movers | Flexors | **3** Biceps brachii, Brachialis, Brachioradialis |
|  |  | Extensors | **3** Triceps brachii, Anconeus, |
|  |  | Pronators | **3** Pronator teres, Pronator quadratus |
|  |  | Supinators | **3** Supinator |
| Hand | Whole hand movers | Flexion | **4** Flexor carpi radialis, Palmaris longus, Flexor carpi ulnaris |
|  |  | Extension | **5** Extensor carpi radialis longus, Extensor carpi radialis brevis, Extensor carpi ulnaris |
|  | Digit II, III, IV movers | Extension- Flexion | **6** Extensor digitorum, Extensor digiti minimi, Extensor indicis, Flexor digitorum profundus, Flexor digitorum superficialis, Lumbricals 1, 2, 3 |
|  |  | Abduction-Adduction | **7** Palmar interossei 1 and 2, Dorsal interossei 1, 2, 3 and 4 |
|  | Digit I movers |  | **8** Abductor pollicis longus, Extensor pollicis brevis, Extensor pollicis longus, Flexor pollicis brevis, Flexor pollicis longus, Opponens pollicis, Opponens digiti minimi, Abductor pollicis brevis, Adductor pollicis, Adductor pollicis accessorius |
|  | Digit V movers |  | **9** Flexor digiti minimi brevis, Abductor digit minimi, Lumbrical 4, Palmar interossei 3 |

**Developmental and functional groups of the bones of the lower limb.**

| **Supragroup** | **Group** | **Subgroup** | **Bones** |
| --- | --- | --- | --- |
| Girdle | Girdle |  | **1** Hip bone (ischium, pubis, ilium in newborn/fetus) |
| Leg | Stylopod |  | **2** Femur, Patella |
|  | Zeugopod |  | **3** Tibia, Fibula |
| Foot | Autopod | Ankle | **4** Calcaneus, Talus, Navicular, Cuboid, Lateral cuneiform, Intermediate cuneiform, Medial cuneiform |
|  |  | Toe I | **5** 1st Metatarsal, Proximal phalanx, Distal phalanx, Lateral sesamoid, Medial sesamoid |
|  |  | Toe II | **6** 2st Metatarsal, Proximal phalanx, Distal phalanx, Middle phalanx |
|  |  | Toe III | **7** 3st Metatarsal, Proximal phalanx, Distal phalanx, Middle phalanx |
|  |  | Toe IV | **8** 4st Metatarsal, Proximal phalanx, Distal phalanx, Middle phalanx |
|  |  | Toe V | **9** 5st Metatarsal, Proximal phalanx, Distal phalanx, Middle phalanx |

**Developmental groups of muscles of the lower limb.**

| **Supragroup** | **Group** | **Subgroup** | **Muscles** |
| --- | --- | --- | --- |
| Girdle | Gluteal region | Medial rotator gluteal group | **1** Tensor fasciae latae, Gluteus minimus, Gluteus medius |
|  |  |  | **2** Gluteus maximus |
|  |  |  | **3** Piriformis |
|  |  | Ischiotrochanteric group | **4** Gemellus superior, Obturator internus, Gemellus inferior, Quadratus femoris |
| Leg | Thigh | Posterior compartment | **5** Biceps femoris, Semitendinosus, Semimembranosus |
|  |  | Anterior compartment | **6** Iliopsoas, Sartorius, Rectus femoris, Vastus lateralis, Vastus intermedius, Vastus medialis |
|  |  | Medial compartment | **7** Pectineus, Adductor magnus, Adductor longus, Adductor brevis, Gracilis, Obturator externus |
|  | Lower Leg | Lateral compartment | **8** Fibularis longus, Fibularis brevis |
|  |  | Anterior compartment | **9** Fibularis tertius, Extensor digitorum longus, Extensor hallucis longus, Tibialis anterior, Extensor digitorum brevis, Extensor halllucis brevis |
|  |  | Posterior compartment | **10** Popliteus, Gastrocnemius, Plantaris, Soleus, Flexor digitorum longus, Flexor hallucis longus, Tibialis posterior |
| Foot | Foot |  | **11** Lumbricals 1, 2, 3 and 4 |
|  |  |  | **11** Abductor hallucis |
|  |  |  | **11** Adductor hallucis |
|  |  | Flexores breves profundi group | **11** Flexor hallucis brevis, Plantar interossei, Dorsal interossei, Flexor digiti minimi brevis |
|  |  |  | **11** Quadratus plantae |
|  |  |  | **11** Abductor digit minimi |
|  |  |  | **11** Flexor digitorum brevis |

**Functional groups of muscles of the lower limb.**

| **Supragroup** | **Group** | **Subgroup** | **Muscles** |
| --- | --- | --- | --- |
| Leg | Femur movers | Flexors | **I** liopsoas |
|  |  | Extensors | **2** Gluteus maximus |
|  |  | Lateral rotators | **3** Gemellus superior, Obturator internus, Gemellus inferior, Quadratus femoris, Piriformis, Oburator externus |
|  |  | Medial rotators | **4** Tensor fasciae latae, Gluteus minimus, Gluteus medius |
|  |  | Adductors | **5** Pectineus, Adductor magnus, Adductor longus, Adductor brevis |
|  | Leg movers | Flexors | **6** Biceps femoris, Semitendinosus, Semimembranosus, Sartorius, Gracilis, Popliteus |
|  |  | Extensors | **7** Rectus femoris, Vastus lateralis, Vastus intermedius, Vastus medialis |
| Foot | Whole foot movers | Plantarflexion | **8** Fibularis longus, Fibularis brevis, Gastrocnemius, Plantaris, Soleus, Tibialis posterior |
|  |  | Dorsiflexion | **9** Fibularis tertius, Tibialis anterior |
|  | Digits II, III, IV movers | Extensors-Flexors | **10** Lumbricals 1, 2, 3, Flexor digitorum longus, Extensor digitorum longus, Extensor digitorum brevis, Flexor digitorum brevis |
|  |  | Abductors-Adductors | **11** Plantar interossei 1 and 2, Dorsal interossei 1, 2, 3 and 4 |
|  | Digit I movers |  | **12** Abductor hallucis, Extensor hallucis longus, Flexor hallucis brevis, Adductor hallucis, Flexor hallucis longus, Quadratus plantae, Extensor hallucis longus, Extensor halllucis brevis |
|  | Digit V movers |  | **13** Flexor digiti minimi brevis, Abductor digiti minimi, Lumbrical 4, Plantar interossei 3 |
