# Supplementary material for: Anatomical Network Comparison of Human Upper and Lower, Newborn and Adult, and Normal and Abnormal Limbs, with Notes on Development, Pathology and Limb Serial Homology vs. Homoplasy
Source: PLoS One. 2015 Oct 9;10(10):e0140030. doi: 10.1371/journal.pone.0140030 (PMC4599883; doi:10.1371/journal.pone.0140030)
Supplement: S1 Results — (DOCX) [file pone.0140030.s031.docx]

**SI.Para: Parameters of the upper and lower limbs (UL, LL)**

**Parameters of the adult/newborn upper skeletal**

| Nodes | 34 |
| --- | --- |
| Links | 44 |
| Density | 0.07843137 |
| Clustering coefficient | 0.1705882 |
| Shortest path length | 4.237903 |
| Heterogeneity | 0.6459336 |
| Modules | 9 |
| Q-value | 0.4702996 |
| Is connected? | FALSE |
| Is small-world? (largest cluster) | TRUE |
| Is hierarchical? | FALSE |
| P(k) fit | Alpha = 1.822; R square = 0.777 |
| C(k) fit | Alpha = -1.018; R square = 0.547 |

**Parameters of the adult/newborn upper muscular**

| Nodes | 57 |
| --- | --- |
| Links | 4 |
| Density | 0.002506266 |
| Clustering coefficient | 0 |
| Shortest path length | 1.6 |
| Heterogeneity | 4.136753 |
| Modules | 57 |
| Q-value | 0 |
| Is connected? | FALSE |
| Is small-world? (largest cluster) | NA |
| Is hierarchical? | FALSE |
| P(k) fit | Alpha = 1.179 / R square = 0.776 |
| C(k) fit | NA |

**Parameters for the adult/newborn upper musculoskeletal**

| Nodes | 91 |
| --- | --- |
| Links | 184 |
| Density | 0.04493284 |
| Clustering coefficient | 0.3451401 |
| Shortest path length | 3.319414 |
| Heterogeneity | 0.9583683 |
| Modules | 7 |
| Q-value | 0.5540525 |
| Is connected? | TRUE |
| Is small-world? (largest cluster) | TRUE |
| Is hierarchical? | TRUE |
| P(k) fit | Alpha = 1.753 / R square = 0.937 |
| C(k) fit | Alpha = 0.842 / R square = 0.899 |

**Parameters of the adult lower skeletal**

| Nodes | 33 |
| --- | --- |
| Links | 41 |
| Density | 0.07765152 |
| Clustering coefficient | 0.1444444 |
| Shortest path length | 4.354839 |
| Heterogeneity | 0.6292834 |
| Modules | 11 |
| Q-value | 0.5252825 |
| Is connected? | FALSE |
| Is small-world? (largest cluster) | TRUE |
| Is hierarchical? | TRUE |
| P(k) fit | Alpha = 1.631 / R square = 0.755 |
| C(k) fit | Alpha = -1.349 / R square = 0.838 |

**Parameters of the adult lower muscular**

| Nodes | 57 |
| --- | --- |
| Links | 6 |
| Density | 0.003759398 |
| Clustering coefficient | 0 |
| Shortest path length | 1.625 |
| Heterogeneity | 3.446012 |
| Modules | 51 |
| Q-value | 0.2777778 |
| Is connected? | FALSE |
| Is small-world? (largest cluster) | FALSE |
| Is hierarchical? | FALSE |
| P(k) fit | Alpha = 1.232 / R square = 0.709 |
| C(k) fit | NA |

**Parameters of the adult lower musculoskeletal**

| Nodes | 90 |
| --- | --- |
| Links | 197 |
| Density | 0.04918851 |
| Clustering coefficient | 0.3522471 |
| Shortest path length | 3.261423 |
| Heterogeneity | 0.9011572 |
| Modules | 9 |
| Q-value | 0.5579634 |
| Is connected? | TRUE |
| Is small-world? (largest cluster) | TRUE |
| Is hierarchical? | FALSE |
| P(k) fit | Alpha = 1.636 / R square = 0.931 |
| C(k) fit | Alpha = 0.789 / R square = 0.666 |

**Parameters of the newborn lower skeletal**

| Nodes | 35 |
| --- | --- |
| Links | 46 |
| Density | 0.07731092 |
| Clustering coefficient | 0.2304762 |
| Shortest path length | 4.522727 |
| Heterogeneity | 0.591427 |
| Modules | 10 |
| Q-value | 0.5718336 |
| Is connected? | FALSE |
| Is small-world? (largest cluster) | TRUE |
| Is hierarchical? | FALSE |
| P(k) fit | Alpha = 1.627 / R square = 0.715 |
| C(k) fit | Alpha = -1.005 / R square = 0.296 |

**Parameters of the newborn lower muscular**

| Nodes | 57 |
| --- | --- |
| Links | 6 |
| Density | 0.003759398 |
| Clustering coefficient | 0 |
| Shortest path length | 1.625 |
| Heterogeneity | 3.446012 |
| Modules | 51 |
| Q-value | 0.2777778 |
| Is connected? | FALSE |
| Is small-world? (largest cluster) | NA |
| Is hierarchical? | FALSE |
| P(k) fit | Alpha = 1.232 / R square = 0.709 |
| C(k) fit | NA |

**Parameters of the newborn lower musculoskeletal**

| Nodes | 92 |
| --- | --- |
| Links | 206 |
| Density | 0.04921166 |
| Clustering coefficient | 0.35434 |
| Shortest path length | 3.285953 |
| Heterogeneity | 0.8481938 |
| Modules | 9 |
| Q-value | 0.5450796 |
| Is connected? | TRUE |
| Is small-world? (largest cluster) | TRUE |
| Is hierarchical? | FALSE |
| P(k) fit | Alpha = 1.822 / R square = 0.916 |
| C(k) fit | Alpha = 0.675 / R square = 0.593 |

**Parameters of the T18 upper left skeletal**

| Nodes | 34 |
| --- | --- |
| Links | 44 |
| Density | 0.07843137 |
| Clustering coefficient | 0.1705882 |
| Shortest path length | 4.237903 |
| Heterogeneity | 0.6459336 |
| Modules | 9 |
| Q-value | 0.4702996 |
| Is connected? | FALSE |
| Is small-world? (largest cluster) | TRUE |
| Is hierarchical? | FALSE |
| P(k) fit | Alpha = 1.822 / R square = 0.777 |
| C(k) fit | Alpha = -1.018 / R square = 0.547 |

**Parameters of the T18 upper left muscular**

| Nodes | 55 |
| --- | --- |
| Links | 20 |
| Density | 0.01346801 |
| Clustering coefficient | 0.1515152 |
| Shortest path length | 1.46875 |
| Heterogeneity | 1.281546 |
| Modules | 38 |
| Q-value | 0.835 |
| Is connected? | FALSE |
| Is small-world? (largest cluster) | TRUE |
| Is hierarchical? | TRUE |
| P(k) fit | Alpha = 2.102 / R square = 0.798 |
| C(k) fit | Alpha = 3.419 / R square = 1 |

**Parameters of the T18 upper left musculoskeletal**

| Nodes | 89 |
| --- | --- |
| Links | 186 |
| Density | 0.05005107 |
| Clustering coefficient | 0.3866731 |
| Shortest path length | 3.297753 |
| Heterogeneity | 0.8375084 |
| Modules | 9 |
| Q-value | 0.5213844 |
| Is connected? | TRUE |
| Is small-world? (largest cluster) | TRUE |
| Is hierarchical? | TRUE |
| P(k) fit | Alpha = 1.82 / R square = 0.923 |
| C(k) fit | Alpha = 0.838 / R square = 0.902 |

**Parameters of the T18 upper right skeletal**

| Nodes | 34 |
| --- | --- |
| Links | 44 |
| Density | 0.07843137 |
| Clustering coefficient | 0.1705882 |
| Shortest path length | 4.237903 |
| Heterogeneity | 0.6459336 |
| Modules | 9 |
| Q-value | 0.4702996 |
| Is connected? | FALSE |
| Is small-world? (largest cluster) | TRUE |
| Is hierarchical? | FALSE |
| P(k) fit | Alpha = 1.822 / R square = 0.777 |
| C(k) fit | Alpha = -1.018 / R square = 0.547 |

**Parameters of the T18 upper right muscular**

| Nodes | 52 |
| --- | --- |
| Links | 12 |
| Density | 0.009049774 |
| Clustering coefficient | 0 |
| Shortest path length | 2.305556 |
| Heterogeneity | 1.79293 |
| Modules | 41 |
| Q-value | 0.6701388 |
| Is connected? | FALSE |
| Is small-world? (largest cluster) | FALSE |
| Is hierarchical? | FALSE |
| P(k) fit | Alpha = 2.199 / R square = 0.922 |
| C(k) fit | NA |

**Parameters of the T18 upper right musculoskeletal**

| Nodes | 86 |
| --- | --- |
| Links | 180 |
| Density | 0.04924761 |
| Clustering coefficient | 0.3740819 |
| Shortest path length | 3.252661 |
| Heterogeneity | 0.9017014 |
| Modules | 6 |
| Q-value | 0.5630401 |
| Is connected? | FALSE |
| Is small-world? (largest cluster) | TRUE |
| Is hierarchical? | TRUE |
| P(k) fit | Alpha = 1.763 / R square = 0.933 |
| C(k) fit | Alpha = 0.88 / R square = 0.826 |

**Parameters of the T18 lower left skeletal**

| Nodes | 35 |
| --- | --- |
| Links | 46 |
| Density | 0.07731092 |
| Clustering coefficient | 0.2304762 |
| Shortest path length | 4.522727 |
| Heterogeneity | 0.591427 |
| Modules | 10 |
| Q-value | 0.5718336 |
| Is connected? | FALSE |
| Is small-world? (largest cluster) | TRUE |
| Is hierarchical? | FALSE |
| P(k) fit | Alpha = 1.627 / R square = 0.715 |
| C(k) fit | Alpha = -1.005 / R square = 0.296 |

**Parameters T18 lower left muscular**

| Nodes | 57 |
| --- | --- |
| Links | 7 |
| Density | 0.004385965 |
| Clustering coefficient | 0 |
| Shortest path length | 1.588235 |
| Heterogeneity | 3.007734 |
| Modules | 50 |
| Q-value | 0.4489796 |
| Is connected? | FALSE |
| Is small-world? (largest cluster) | FALSE |
| Is hierarchical? | FALSE |
| P(k) fit | Alpha = 1.365 / R square = 0.709 |
| C(k) fit | NA |

**Parameters of the T18 lower left musculoskeletal**

| Nodes | 92 |
| --- | --- |
| Links | 206 |
| Density | 0.04921166 |
| Clustering coefficient | 0.3599724 |
| Shortest path length | 3.287387 |
| Heterogeneity | 0.8469008 |
| Modules | 7 |
| Q-value | 0.550099 |
| Is connected? | TRUE |
| Is small-world? (largest cluster) | TRUE |
| Is hierarchical? | FALSE |
| P(k) fit | Alpha = 1.822 / R square = 0.916 |
| C(k) fit | Alpha = 0.825 / R square = 0.395 |

**Parameters of the T18 lower right skeletal**

| Nodes | 35 |
| --- | --- |
| Links | 46 |
| Density | 0.07731092 |
| Clustering coefficient | 0.2304762 |
| Shortest path length | 4.522727 |
| Heterogeneity | 0.591427 |
| Modules | 10 |
| Q-value | 0.5718336 |
| Is connected? | FALSE |
| Is small-world? (largest cluster) | TRUE |
| Is hierarchical? | FALSE |
| P(k) fit | Alpha = 1.627 / R square = 0.715 |
| C(k) fit | Alpha = -1.005 / R square = 0.296 |

**Parameters of the T18 lower right muscular**

| Nodes | 56 |
| --- | --- |
| Links | 7 |
| Density | 0.004545455 |
| Clustering coefficient | 0 |
| Shortest path length | 1.588235 |
| Heterogeneity | 2.978712 |
| Modules | 49 |
| Q-value | 0.4489796 |
| Is connected? | FALSE |
| Is small-world? (largest cluster) | FALSE |
| Is hierarchical? | FALSE |
| P(k) fit | Alpha = 1.365 / R square = 0.709 |
| C(k) fit | NA |

**Parameters of the T18 lower right musculoskeletal**

| Nodes | 91 |
| --- | --- |
| Links | 203 |
| Density | 0.04957265 |
| Clustering coefficient | 0.349939 |
| Shortest path length | 3.298413 |
| Heterogeneity | 0.8301564 |
| Modules | 7 |
| Q-value | 0.5559708 |
| Is connected? | TRUE |
| Is small-world? (largest cluster) | TRUE |
| Is hierarchical? | FALSE |
| P(k) fit | Alpha = 1.843 / R square = 0.911 |
| C(k) fit | Alpha = 0.736 / R square = 0.559 |
